# Copper-catalyzed one-pot domino reactions *via* C–H bond activation: synthesis of 3-aroylquinolines from 2-aminobenzylalcohols and propiophenones under metal–organic framework catalysis†

**DOI:** 10.1039/c8ra05459b

**Published:** 2018-09-07

**Authors:** Ha V. Dang, Hoang T. B. Le, Loan T. B. Tran, Hiep Q. Ha, Ha V. Le, Nam T. S. Phan

**Affiliations:** Faculty of Chemical Engineering, HCMC University of Technology, VNU-HCM 268 Ly Thuong Kiet, District 10 Ho Chi Minh City Viet Nam ptsnam@hcmut.edu.vn +84 8 38637504 +84 8 38647256 ext. 5681

## Abstract

A Cu_2_(OBA)_2_(BPY) metal–organic framework was utilized as a productive heterogeneous catalyst for the synthesis of 3-aroylquinolines *via* one-pot domino reactions of 2-aminobenzylalcohols with propiophenones. This Cu-MOF was considerably more active towards the one-pot domino reaction than a series of transition metal salts, as well as nano oxide and MOF-based catalysts. The MOF-based catalyst was reusable without a significant decline in catalytic efficiency. To the best of our knowledge, the transformation of 2-aminobenzylalcohols to 3-aroylquinolines was not previously reported in the literature, and this protocol would be complementary to previous strategies for the synthesis of these valuable heterocycles.

## Abstract 2

Cu_2_(OBA)_2_(BPY) metal–organic framework was utilized as a productive heterogeneous catalyst for the synthesis of 3-aroylquinolines *via* one-pot domino reactions of 2-aminobenzylalcohols with propiophenones.

## Graphical abstract

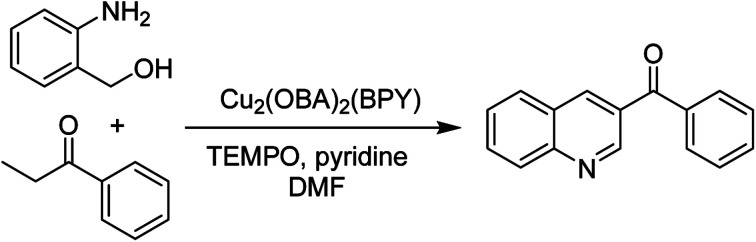

## 1. Introduction

Quinolines have been recognized as valuable scaffolds commonly existing in numerous bioactive natural products and synthetic chemicals with versatile pharmacological and medicinal properties.^1–4^ In particular, 3-acylquinolines have received remarkable interest as many of them are clinically used as drugs and pharmaceutical candidates.^5,6^ Consequently, several strategies have recently been explored for the synthesis of these heterocycles. Jalal *et al.* previously reported a one-pot synthesis of 3-acylquinolines *via* alkyne-carbonyl metathesis/detosylation/aromatization of *N*-propargyl-2-aminobenzaldehyde/acetophenone derivatives using FeCl_3_ catalyst.^7^ Kuriyama *et al.* demonstrated a one-pot synthesis of 3-acylquinolines through palladium-catalyzed 1,2-addition and subsequent oxidation.^8^ Luo *et al.* synthesized 4-substituted 3-aroylquinolines by utilizing ZnCl_2_-catalyzed Friedländer-type reaction of *o*-aminoaryl ketones with enaminones.^9^ Wan *et al.* developed a protocol to obtain 3-acylquinolines from TfOH-catalyzed domino reactions between *N*,*N*-dimethyl enaminones and anilines.^10^ Wang *et al.* obtained 3-acylquinolines from Cu(OAc)_2_-catalyzed one-pot cascade reactions of 2-aminoaryl aldehydes/ketones with saturated ketones.^11^ Wakade *et al.* reported a transition metal-free cascade protocol to produce 3-acylquinolines using acetophenones, anthranils, and DMSO in the presence of K_2_S_2_O_8_.^12^

Metal–organic frameworks (MOFs) constructed by organic linkers and metal ions/clusters, featuring crystalline porous structures, have been considerably studied during the last twenty years, and investigations of their promising applications are in progress.^13–18^ Distinct properties such as a large surface area and high porosity, being accessible *via* continuous and permeable channels, would enable MOFs to be good catalytic materials.^19–24^ The utilization of solid catalysts offers easy catalyst recovery and recycling, minimizing toxic and hazardous wastes, and MOFs as catalysts would be a good option for many organic transformations.^25–27^ In the porous framework, both organic linkers and metal clusters could be useful for catalysis, facilitating the interaction between active sites and reactants.^22,28–30^ Although long lists of MOFs have been published with detailed data on syntheses and characterizations, more efforts should be devoted to their applications in catalysis.^31–34^ During the past decade, a long series of organic reactions have been conducted utilizing MOFs as catalysts with fascinating results being reported.^21,35–39^ In this work, we would like to report the synthesis of 3-aroylquinolines *via* one-pot domino reactions of 2-aminobenzylalcohols with propiophenones under Cu_2_(OBA)_2_(BPY) metal–organic framework catalysis. To our best knowledge, this transformation was not previously reported in the literature. The MOF-based catalyst was recovered and reutilized without noticeable deterioration in catalytic performance.

## 2. Experimental

In a representative experiment, 2-aminobenzyl alcohol (0.0246 g, 0.2 mmol), propiophenone (0.0536 g, 0.4 mmol), and Cu_2_(OBA)_2_(BPY) catalyst were added to a pressurized vial. The catalyst concentration was calculated regarding copper/2′-aminobenzyl alcohol molar ratio. Subsequently, TEMPO (2,2,6,6-tetramethylpiperidine-*N*-oxyl) (0.0624 g, 0.4 mmol) as a oxidant, pyridine (0.00237 g, 0.3 mmol) as a ligand, and DMF (0.5 mL) were added to the reactor. The reaction mixture was magnetically stirred at 120 °C for 16 h. After the reaction was complete, the vial was cooled to ambient temperature, and diphenyl ether as internal standard was added into the mixture. The reaction yield was monitored by withdrawing samples from the reaction mixture, quenching with brine (2 mL). The organic components were consequently extracted into ethyl acetate (2 mL), dried over anhydrous Na_2_SO_4_, and analyzed by GC with diphenyl ether as internal standard. To isolate the desired product, the reaction mixture was diluted with ethyl acetate (30 mL). The ethyl acetate solution was washed with brine solutions (4 × 10 mL). The organic layer was subsequently dried over anhydrous Na_2_SO_4_ and concentrated under reduced pressure. The resulting residue was purified on silica gel by column chromatography (ethyl acetate/hexane = 1 : 4), affording phenyl(quinolin-3-yl)methanone as a light yellow crystalline solid. ^1^H NMR and ^13^C NMR were utilized to confirm product structure.

## 3. Results and discussion

**Scheme 1:**
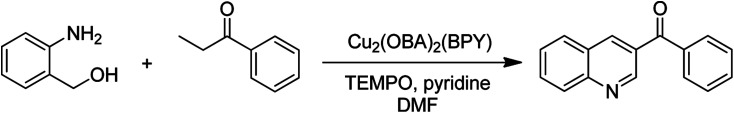
The one-pot domino reaction between 2-aminobenzylalcohol and propiophenone utilizing Cu_2_(OBA)_2_(BPY) catalyst.

The Cu_2_(OBA)_2_(BPY) was synthesized in 71% yield from copper(ii) nitrate trihydrate, 4,4′-oxybis(benzoic) acid, and 4,4′-bipyridine utilizing a literature protocol.^40^ The Cu-MOF was subsequently characterized by conventional analysis techniques (Fig. S1–S7†). The framework was used as a heterogeneous catalyst for the one-pot domino reaction between 2-aminobenzylalcohol and propiophenone to produce phenyl(quinolin-3-yl)methanone as the major product (Scheme 1). Initially, reaction conditions were screened to maximize the yield of the desired product (Tables 1 and 2). The impact of temperature on the yield of the 3-aroylquinoline was then studied (entries 1–6, Table 1). The reaction was conducted in DMF for 16 h at 10 mol% catalyst, with 2 equivalents of propiophenone, in the presence of 2 equivalents of TEMPO and 1.5 equivalents of pyridine, at room temperature, 60 °C, 80 °C, 100 °C, 120 °C, and 140 °C, respectively. The reaction proceeded slowly at 80 °C, generating the desired product in only 3% yield. Boosting the temperature to 100 °C led to 38% yield being recorded. The transformation was remarkably accelerated at 120 °C, and 91% yield of phenyl(quinolin-3-yl)methanone was achieved under this condition (entry 5, Table 1). Nevertheless, it was noted that increasing the temperature to 140 °C did not result in higher yield.

**Table 1:** Screening of reaction conditions, regarding temperature, solvent, reactant concentration, and catalyst amount^a^

Entry	Temperature (°C)	Solvent	Concentration^b^ (M)	Catalyst amount (mol%)	Yield^c^ (%)
1	RT	DMF	0.4	10	2
2	60	DMF	0.4	10	2
3	80	DMF	0.4	10	3
4	100	DMF	0.4	10	38
5	120	DMF	0.4	10	91
6	140	DMF	0.4	10	91
7	120	NMP	0.4	10	16
8	120	Ethylbenzene	0.4	10	40
9	120	Cumene	0.4	10	48
10	120	DMSO	0.4	10	55
11	120	1,2-DCB	0.4	10	64
12	120	*p*-Xylene	0.4	10	70
13	120	Toluene	0.4	10	77
14	120	DMA	0.4	10	78
15	120	DMF	0.4	10	91
16	120	DMF	0.8	10	83
17	120	DMF	0.4	10	91
18	120	DMF	0.2	10	75
19	120	DMF	0.1	10	45
20	120	DMF	0.06	10	23
21	120	DMF	0.05	10	15
22	120	DMF	0.4	0	0
23	120	DMF	0.4	1	10
24	120	DMF	0.4	3	21
25	120	DMF	0.4	5	83
26	120	DMF	0.4	7	87
27	120	DMF	0.4	10	91
28	120	DMF	0.4	20	92

a Reaction conditions: 2-aminobenzylalcohol (0.2 mmol); propiophenone (0.4 mmol); pyridine (0.3 mmol); TEMPO (0.4 mmol); 16 h.

b 2-aminobenzylalcohol concentration.

c GC yield.

**Table 2:** Screening of reaction conditions, regarding oxidant, oxidant amount, ligand, and ligand amount^a^

Entry	Oxidant	Oxidant amount (equiv.)	Ligand	Ligand amount (equiv.)	Yield^b^ (%)
1	K_2_S_2_O_8_	2	Pyridine	1.5	2
2	Oxygen	2	Pyridine	1.5	3
3	H_2_O_2_	2	Pyridine	1.5	3
4	CHP	2	Pyridine	1.5	3
5	TBHP/decane	2	Pyridine	1.5	3
6	TBHP/water	2	Pyridine	1.5	3
7	DTBP	2	Pyridine	1.5	3
8	TEMPO	2	Pyridine	1.5	91
9	TEMPO	0	Pyridine	1.5	0
10	TEMPO	1	Pyridine	1.5	60
11	TEMPO	1.5	Pyridine	1.5	62
12	TEMPO	2	Pyridine	1.5	91
13	TEMPO	2.5	Pyridine	1.5	93
14	TEMPO	3	Pyridine	1.5	93
15	TEMPO	2	PDCA	1.5	0
16	TEMPO	2	HMTA	1.5	0
17	TEMPO	2	Ph_3_P	1.5	0
18	TEMPO	2	4,4′-Dipyridine	1.5	46
19	TEMPO	2	2-Aminopyridine	1.5	47
20	TEMPO	2	PHEN	1.5	56
21	TEMPO	2	Triethylamine	1.5	63
22	TEMPO	2	TMEDA	1.5	72
23	TEMPO	2	Pyridine	1.5	91
24	TEMPO	2	Pyridine	0	32
25	TEMPO	2	Pyridine	1	62
26	TEMPO	2	Pyridine	1.5	91
27	TEMPO	2	Pyridine	2	91
28	TEMPO	2	Pyridine	2.5	91

a Reaction conditions: 2-aminobenzylalcohol (0.2 mmol); propiophenone (0.4 mmol); Cu_2_(OBA)_2_(BPY) (10 mol%); DMF (0.5 mL); 16 h; CHP: cumyl hydroperoxide; TBHP/decane: *tert*-butyl hydroperoxide in decane; TBHP/water: *tert*-butyl hydroperoxide in water; DTBP: di-*tert*-butylperoxide; PDCA: 2,5-pyridinedicarboxylic acid; HMTA: hexanmethylene tetraamine; PHEN: 1,10-phenanthroline; TMEDA: tetramethylethylenediamine.

b GC yield.

As the one-pot domino reaction between 2-aminobenzylalcohol and propiophenone to produce phenyl(quinolin-3-yl)methanone progressed under heterogeneous catalysis conditions, the solvent would play an important role. Wang *et al.* previously screened several solvents for the synthesis of 3-acylquinolines using the Cu(OAc)_2_-catalyzed one-pot cascade reactions of 2-aminoaryl aldehydes/ketones with saturated ketones, and pointed out that toluene was the best solvent for this reaction.^11^ A variety of solvents were then tested for the Cu_2_(OBA)_2_(BPY)-catalyzed reaction to improve the yield of phenyl(quinolin-3-yl)methanone (entries 7–15, Table 1). The reaction was carried out at 120 °C for 16 h with 10 mol% catalyst, using 2 equivalents of propiophenone, in the presence of 2 equivalents of TEMPO and 1.5 equivalents of pyridine. NMP was not suitable for the reaction, affording the desired product in only 16% yield. Ethylbenzene and cumene displayed better performance, with 40% and 40% yields being noticed, respectively. Average yields were observed for the reaction conducted in DMSO, and 1,2-dichlorobenzene. Performing the one-pot domino reaction in non-polar solvents such as *p*-xylene, and toluene offered higher yields. Similarly, the reaction conducted in DMA progressed to 78% yield. Among these solvents, DMF emerged as the best candidate for the reaction, producing the desired product in 91% yield (entry 15, Table 1).

One more issue to be considered is influence of 2-aminobenzylalcohol concentration (*i.e.* the solvent volume) on the yield of the 3-aroylquinoline product (entries 16–21, Table 1). The reaction was conducted in DMF at 120 °C for 16 h with 10 mol% catalyst, using 2 equivalents of propiophenone, in the presence of 2 equivalents of TEMPO and 1.5 equivalents of pyridine. At 2-aminobenzylalcohol concentration of 0.8 M, the reaction afforded 83% yield of phenyl(quinolin-3-yl)methanone. Lowering its concentration to 0.4 M led to an enhancement in the reaction yield (entry 17, Table 1). However, decreasing this concentration to lower than 0.4 M resulted in a noticeable decline in the yield of the major product. Additionally, the catalyst amount displayed a considerable influence on the yield of phenyl(quinolin-3-yl)methanone, having utilized 1 mol%, 3 mol%, 5 mol%, 7 mol%, 10 mol%, and 20 mol% Cu_2_(OBA)_2_(BPY) for the reaction (entries 22–28, Table 1). It was noted that no phenyl(quinolin-3-yl)methanone was detected in the absence of the catalyst, verifying that using the Cu-MOF was necessary for the one-pot domino reaction. As anticipated, increasing the catalyst amount accelerated the transformation noticeably. Best result was observed in the presence of 10 mol% catalyst, with 91% yield of the major product being recorded (entry 27, Table 1). Nevertheless, utilizing more than 10 mol% catalyst did not led to higher yield.

Comparable to other reactions progressed *via* C–H bond activation, utilizing an oxidant was required for the one-pot domino reaction between 2-aminobenzylalcohol and propiophenone to produce phenyl(quinolin-3-yl)methanone. We therefore tested the performance of different oxidants, including K_2_S_2_O_8_, oxygen, H_2_O_2_, cumyl hydroperoxide, *tert*-butyl hydroperoxide in decane, *tert*-butyl hydroperoxide in water, di-*tert*-butylperoxide, and TEMPO, respectively (entries 1–8, Table 2). The reaction was conducted in DMF at 120 °C for 16 h with 10 mol% catalyst, using 2 equivalents of propiophenone, in the presence of 1.5 equivalents of pyridine and 2 equivalents of an oxidant. Interestingly, it was noticed that among these oxidants, TEMPO was the only candidate for the one-pot domino reaction, producing the expected 3-aroylquinoline in 91% yield (entry 8, Table 2). Other oxidants were almost ineffective for the reaction, with 2–3% yields being detected. Additionally, the transformation was also controlled by the amount of the TEMPO, having investigated the impact of different TEMPO amounts on the yield of phenyl(quinolin-3-yl)methanone (entries 9–14, Table 2). It was noticed that no product was observed in the absence of TEMPO. Utilizing 1 equivalent of TEMPO led to 60% yield being recorded. Extending the amount of TEMPO to 2 equivalents offered 91% yield (entry 12, Table 2). It was noticed that utilizing more than 2 equivalents of TEMPO did not enhance the yield of phenyl(quinolin-3-yl)methanone remarkably.

Experimental results indicated that the one-pot domino reaction between 2-aminobenzylalcohol and propiophenone proceeded slowly in the absence of ligand, suggesting that utilizing the ligand was important for the reaction. A series of ligands were subsequently utilized for the reaction, including 2,5-pyridinedicarboxylic acid, hexanmethylene tetraamine, triphenylphosphine, 4,4′-dipyridine, 2-aminopyridine, 1,10-phenanthroline, triethylamine, tetramethylethylenediamine, and pyridine, respectively (entries 15–23, Table 2). The reaction was conducted in DMF at 120 °C for 16 h with 10 mol% catalyst, using 2 equivalents of propiophenone, in the presence of 2 equivalents of TEMPO and 1.5 equivalents of a ligand. 2,5-Pyridinedicarboxylic acid, hexanmethylene tetraamine, and triphenylphosphine were completely ineffective for the reaction. Using 4,4′-dipyridine, and 2-aminopyridine, the reaction afforded 46% and 47% yields, respectively. 1,10-Phenanthroline, triethylamine, and tetramethylethylenediamine displayed better performance for the transformation. Among these ligands, pyridine emerged as the most appropriate candidate for the formation of phenyl(quinolin-3-yl)methanone, with 91% yield being recorded (entry 23, Table 2). Moreover, the amount of pyridine displayed a significant impact on the one-pot domino reaction (entries 24–28, Table 2). Without pyridine, only 32% yield of phenyl(quinolin-3-yl)methanone was detected. Best result was achieved for the reaction utilizing 1.5 equivalents of pyridine (entry 26, Table 2), while extending the amount of pyridine to 2 equivalents or more did not increase the yield of the desired product.

**Fig. 1:**
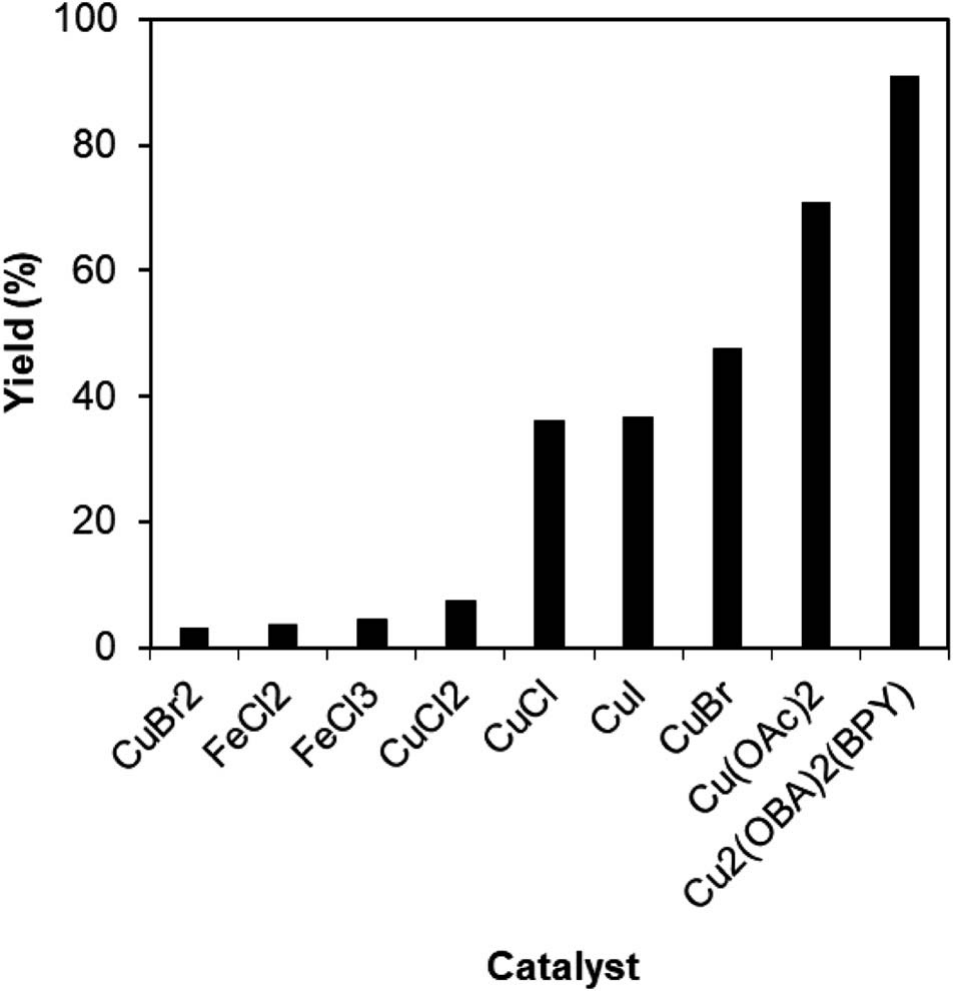
Yields of phenyl(quinolin-3-yl)methanone *vs.* homogeneous catalysts.

**Fig. 2:**
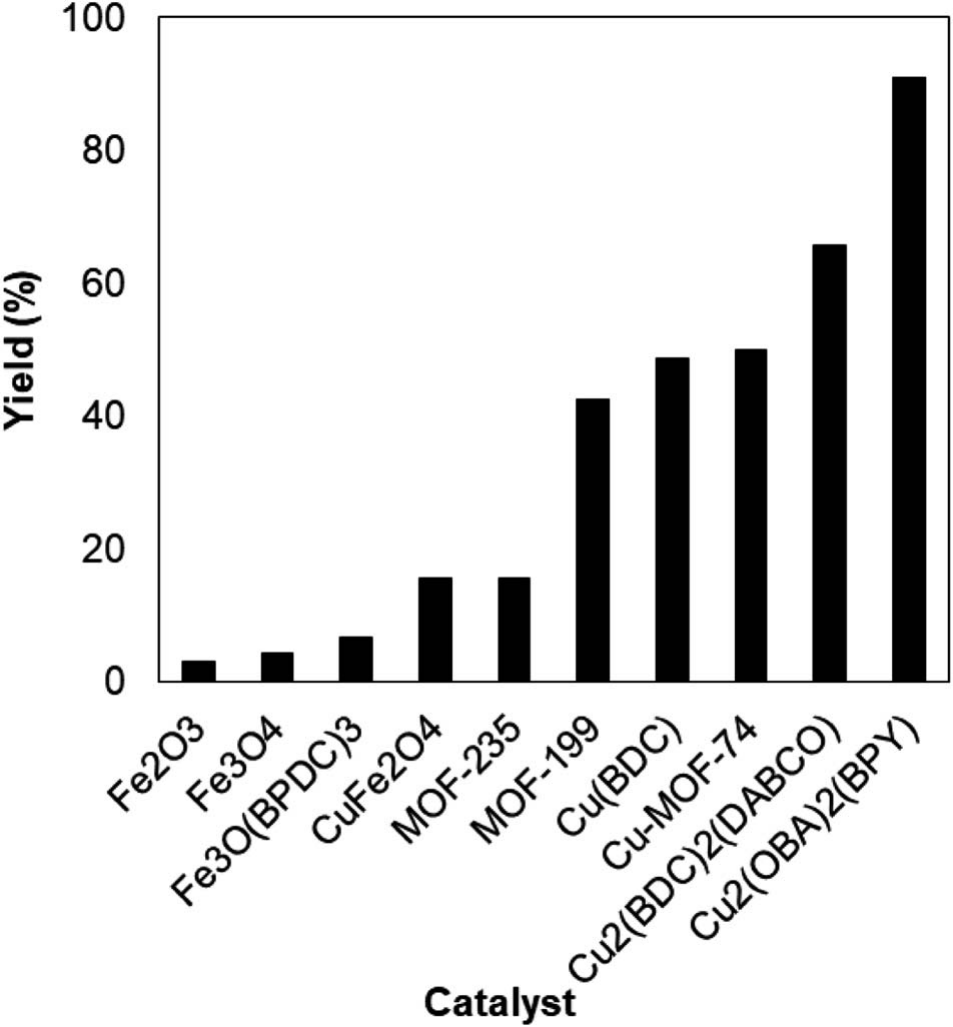
Yields of phenyl(quinolin-3-yl)methanone *vs.* heterogeneous catalysts.

**Scheme 2:**
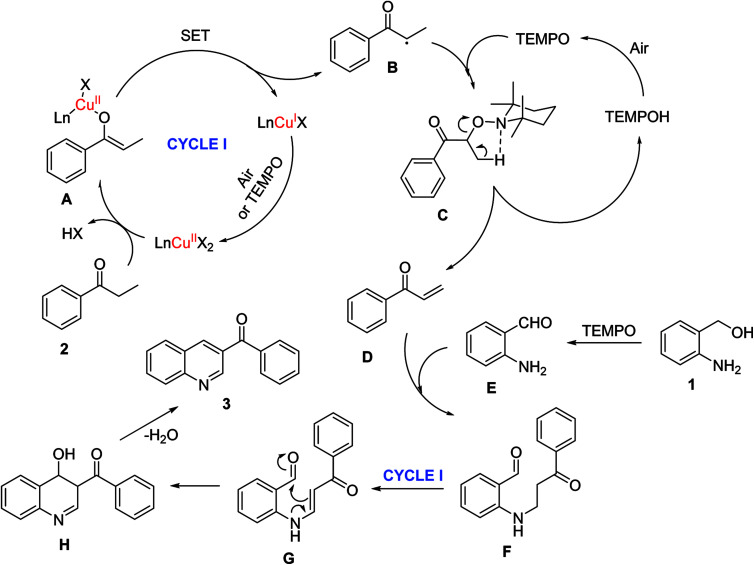
Proposed reaction mechanism.

To verify the superiority of the Cu_2_(OBA)_2_(BPY) catalyst, many homogeneous and heterogeneous catalysts were then utilized for the one-pot domino reaction between 2-aminobenzylalcohol and propiophenone to produce phenyl(quinolin-3-yl)methanone. The reaction was carried out in DMF at 120 °C for 16 h with 10 mol% catalyst, using 2 equivalents of propiophenone, in the presence of 2 equivalents of TEMPO and 1.5 equivalents of pyridine. First, homogeneous catalysis was investigated for the transformation (Fig. 1). CuBr_2_ and CuCl_2_ exhibited very low activity for the reaction, affording only 3% and 7% yields, respectively. Similarly, FeCl_2_ and FeCl_3_ were almost inactive for the one-pot domino reaction. Copper(i) salts were more active towards the formation of phenyl(quinolin-3-yl)methanone, with 36%, 37%, and 47% yields being noticed for the case of CuCl, CuI , and CuBr, respectively. Cu(OAc)_2_ displayed higher catalytic activity, generating the desired product in 71% yield. The Cu_2_(OBA)_2_(BPY)-catalyzed reaction progressed to 91% yield. In the second experiment series, several heterogeneous catalysts were used for the reaction (Fig. 2). Nano Fe_2_O_3_, nano Fe_3_O_4_, and nano CuFe_2_O_4_ should not be used as catalyst for this reaction. Fe-MOFs such as MOF-235 and Fe_3_O(BPDC)_3_ also offered low catalytic activity for this transformation. This might be rationalized based on the fact that 2-aminobenzylalcohol was oxidized to 2-aminobenzoic acid in the presence of an iron-based catalyst, while 2-aminobenzaldehyde should be needed in the catalytic cycle (Scheme 2). MOF-199, Cu(BDC), Cu-MOF-74, and Cu_2_(BDC)_2_(DABCO) exhibited reasonable efficiency, producing the expected product in 43%, 49%, 50%, and 65% yields, respectively. Among these heterogeneous catalysts, the Cu_2_(OBA)_2_(BPY) displayed the best catalytic efficiency. In Cu_2_(BDC)_2_(DABCO) and Cu_2_(OBA)_2_(BPY), the basic nitrogen atoms in DABCO and BPY ligands would facilitate the dehydrogenation on the alpha carbon in propiophenone in the catalytic cycle (Scheme 2). Accordingly, these two catalysts were more active towards the transformation than other Cu-MOFs. Moreover, the longer OBA and BPY linkers in Cu_2_(OBA)_2_(BPY) would make the active sites more accessible to reactants as compared to the case of Cu_2_(BDC)_2_(DABCO).

**Fig. 3:**
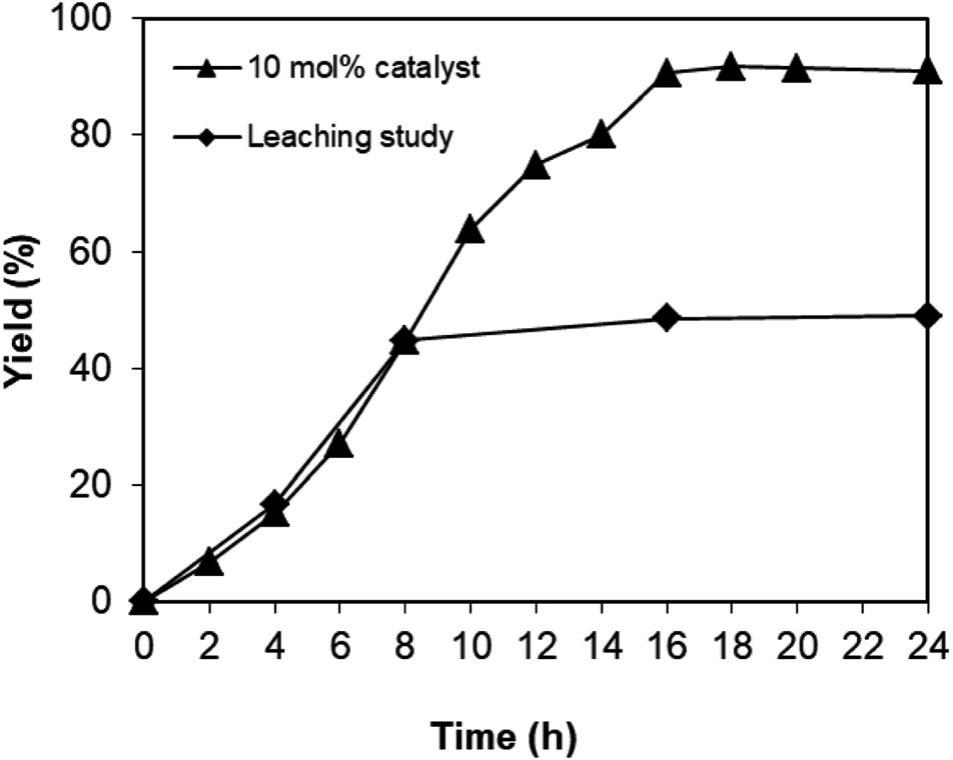
Leaching test confirmed that no additional phenyl(quinolin-3-yl)methanone was produced after catalyst removal.

As the one-pot domino reaction between 2-aminobenzylalcohol and propiophenone to produce phenyl(quinolin-3-yl)methanone utilizing the Cu_2_(OBA)_2_(BPY) catalyst proceeded in liquid phase, it was necessary to verify if soluble copper species contributed to the transformation or not. In some cases, the leaching process might be serious, and the reaction did not proceed *via* truly heterogeneous catalysis conditions. The reaction was conducted in DMF at 120 °C for 24 h with 10 mol% catalyst, using 2 equivalents of propiophenone, in the presence of 2 equivalents of TEMPO and 1.5 equivalents of pyridine. After 8 h reaction time, the Cu-MOF catalyst was removed, and the reaction mixture was transferred to a new vial. The reactor was subsequently heated at 120 °C for additional 16 h, and the yield of phenyl(quinolin-3-yl)methanone was monitored by GC. It was noted that the one-pot domino reaction did not progress noticeably after Cu-MOF catalyst removal, while 91% yield of the 3-aroylquinoline product was obtained in the presence of the catalyst (Fig. 3). These data verified that the one-pot domino reaction utilizing the Cu_2_(OBA)_2_(BPY) catalyst proceeded under heterogeneous catalysis conditions.

**Fig. 4:**
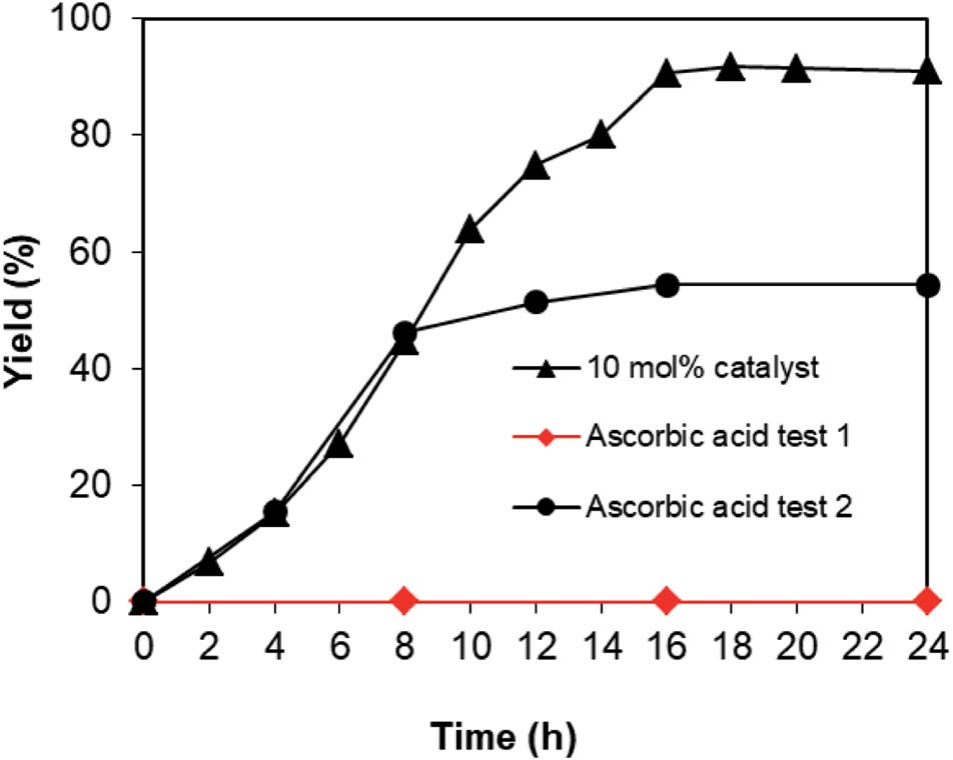
Yields of phenyl(quinolin-3-yl)methanone in the presence of ascorbic acid.

To acquire more data for the one-pot domino reaction between 2-aminobenzylalcohol and propiophenone to produce phenyl(quinolin-3-yl)methanone utilizing the Cu_2_(OBA)_2_(BPY) catalyst, some control experiments were executed. The reaction was conducted in DMF at 120 °C for 24 h with 10 mol% catalyst, using 2 equivalents of propiophenone, in the presence of 2 equivalents of TEMPO and 1.5 equivalents of pyridine. In the first experiment, ascorbic acid as antioxidant was added to the reactor at the beginning of the experiment. Under these conditions, no evidence of phenyl(quinolin-3-yl)methanone was detected in the reaction mixture after 16 h (ascorbic acid test 1, Fig. 4). In the second experiment, the reaction was allowed to proceed under standard conditions for 8 h with 46% yield being recorded. Consequently, ascorbic acid was added to the reactor, and the mixture was heated at 120 °C for further 16 h. GC analysis indicated that 54% yield of the 3-aroylquinoline product was observed. These data disclosed that ascorbic acid interacted with TEMPO or with other radical species generated during the catalytic cycle, and this interaction stopped the one-pot domino transformation. To additionally confirm the generation of radical species in the catalytic cycle, two bulky radical scavengers including 1,1-diphenylethylene (DPE) and 2,6 di-*tert*-butyl-4-methylphenol (BHT) were tested, respectively. Due to the steric effect, these two bulky radical scavengers would not interact with TEMPO. It was observed that only 5% yield was recorded in the presence of DPE, while no trace amount of product was detected in the presence of BHT. These observations verified that the domino reaction would proceed *via* radical pathway. Indeed, Guo *et al.* previously synthesized pyrimidine derivatives in the presence of TEMPO as oxidant, and also utilized DPE and BHT to verify the radical pathway for the transformation.^41^

A plausible reaction pathway was proposed for the one-pot domino reaction between 2-aminobenzylalcohol and propiophenone to produce phenyl(quinolin-3-yl)methanone (Scheme 2). Initially, copper species coordinated with ketone 2 to form the Ln-Cu(ii)-enolate complex A. Under reaction conditions, A underwent single electron transfer (SET) process to generate Cu(i) species, and α-C-centered radical B. This radical consequently interacted with TEMPO to form α-TEMPO-substituted ketone C.^42,43^ Through Cope-like elimination, the intermediates C released 4-OH-TEMPO *via* five centered cyclic transition state to give α,β-unsaturated ketone D.^44^ Indeed, GC-MS analysis indicated the presence of D in the reaction mixture. The TEMPO as an oxidant converted the alcohol group of 1 to aldehyde E, which subsequently underwent a conjugate addition with D to afford β-aminoketone F.^45^ The presence of E in the reaction mixture was also confirmed by GC-MS. Through another Cu(ii)/TEMPO-catalyzed dehydrogenation, F was transformed into enaminone G. Finally, G underwent an intramolecular enamine–ketone condensation to generate product 3*via* the formation of H as an intermediate.^11^

**Fig. 5:**
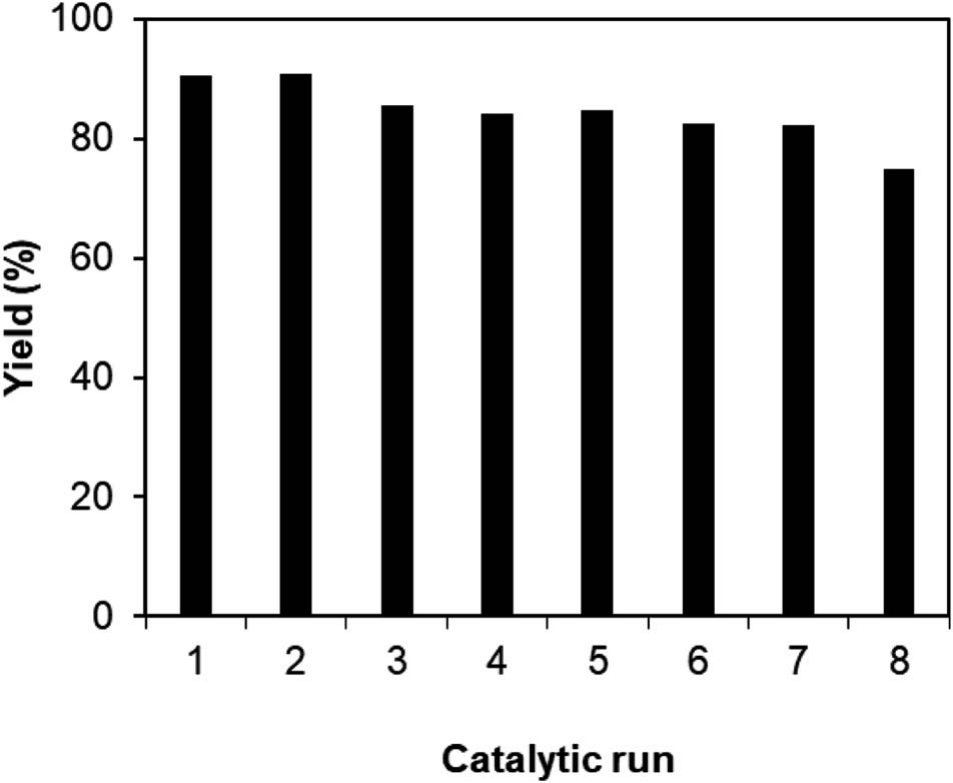
Catalyst reutilizing investigation.

**Fig. 6:**
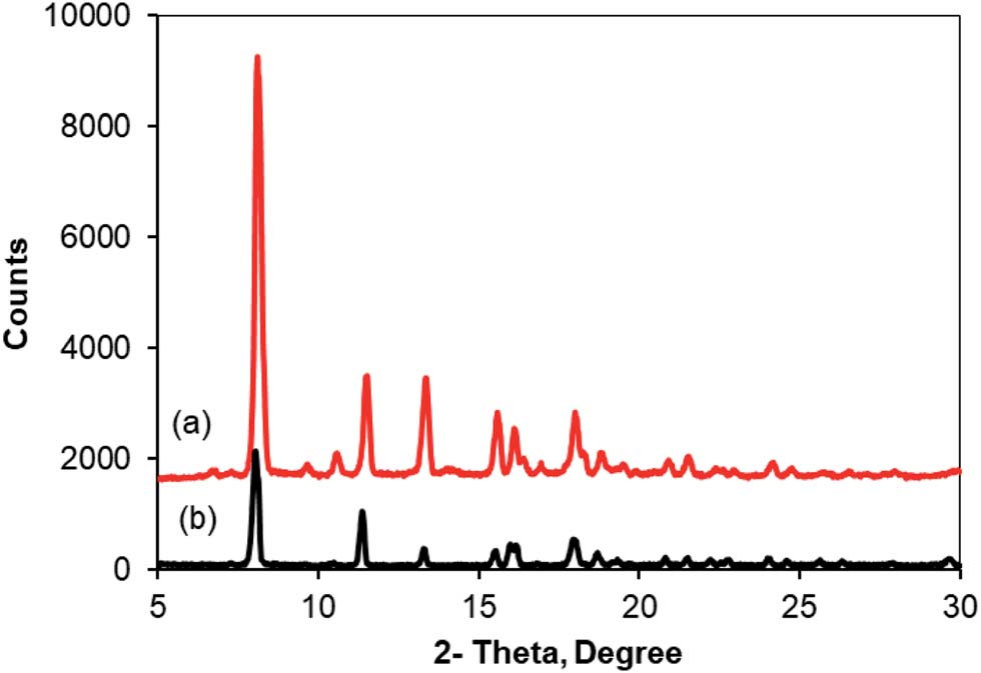
X-ray powder diffractograms of the new (a) and recovered (b) Cu_2_(OBA)_2_(BPY).

**Fig. 7:**
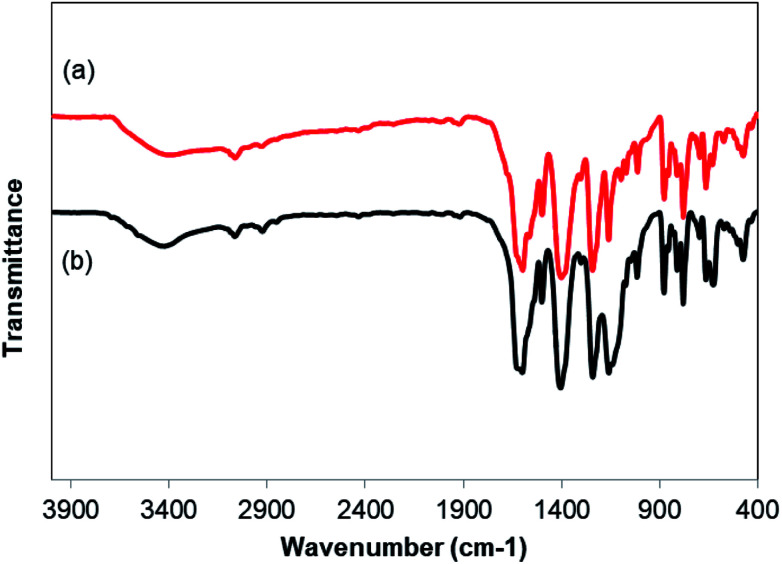
FT-IR results of the new (a) and recovered (b) Cu_2_(OBA)_2_(BPY).

As noted before, the Cu_2_(OBA)_2_(BPY) was more active towards the one-pot domino reaction than a series of conventional transition metal salts, as well as nano oxide and MOF-based catalysts. To additionally highlight the advantage of utilizing this Cu-MOF for the synthesis of phenyl(quinolin-3-yl)methanone *via* the reaction between 2-aminobenzylalcohol and propiophenone, one critical concern should be its reusability in the synthesis of 3-aroylquinolines. The copper–organic framework was consequently investigated for reusability in 8 successive cycles. The reaction was performed in DMF at 120 °C for 16 h with 10 mol% catalyst, using 2 equivalents of propiophenone, in the presence of 2 equivalents of TEMPO and 1.5 equivalents of pyridine. At the end of each run, the Cu-MOF catalyst was isolated by using centrifugation, washed thoroughly with anhydrous DMF and methanol, and subsequently evacuated under vacuum on a Shlenkline at 150 °C for 6 h. After that, the catalyst was reutilized for the next catalytic experiment under standard reaction conditions. Experimental data showed that it was possible to reuse the copper-based framework catalyst for the synthesis of phenyl(quinolin-3-yl)methanone (Fig. 5). Nevertheless, a decline in catalytic activity was observed in the 8^th^ run, with 75% yield being recorded. In addition, the structure of the Cu_2_(OBA)_2_(BPY) was preserved during the catalytic experiment, as verified by XRD (Fig. 6), FT-IR (Fig. 7), and SEM (Fig. S40†) studies.

The scope of this work was subsequently expanded to the Cu_2_(OBA)_2_(BPY)-catalyzed one-pot domino reactions of different 2-aminobenzylalcohols and propiophenones (Table 3). The reaction was conducted in DMF at 120 °C for 16 h with 10 mol% catalyst, using 2 equivalents of propiophenone, in the presence of 2 equivalents of TEMPO and 1.5 equivalents of pyridine. 3-Aroylquinolines were then purified by column chromatography. In the first experiment series, different propiophenones were employed for the reaction with 2-aminobenzylalcohol (entries 1–8, Table 3). Phenyl(quinolin-3-yl)methanone was achieved in 89% yield *via* the reaction between 2-aminobenzylalcohol and propiophenone (entry 1). Halogen-containing propiophenones were reactive towards the reaction, producing (3-chlorophenyl)(quinolin-3-yl)methanone (entry 2), (4-fluorophenyl)(quinolin-3-yl)methanone (entry 3), and (4-bromophenyl)(quinolin-3-yl)methanone (entry 4) in 75%, and 80% yields, respectively. Similarly, (3-nitrophenyl)(quinolin-3-yl)methanone (entry 5), and quinolin-3-yl(2-(trifluoromethyl)phenyl)methanone (entry 6) were obtained in 77% and 92% yields, respectively. Propiophenones containing an electron-donating substituent were good starting materials for the synthesis of 3-aroylquinolines (entries 7 and 8). In the second experiment series, 2-aminobenzylalcohols possessing substituents were utilized for the one-pot domino reactions, affording corresponding 3-aroylquinolines in high yields (entries 9–15, Table 3). Additionally, (2-aminophenyl)(phenyl)methanol was also reactive, producing (4-phenylquinolin-3-yl)(*p*-tolyl)methanone in 63% yield (entry 16).

**Table 3:** Synthesis of 3-aroylquinolines *via* Cu_2_(OBA)_2_(BPY)-catalyzed one-pot domino reactions^a^

Entry	Reactant 1	Reactant 2	Product	Yield^b^ (%)
1	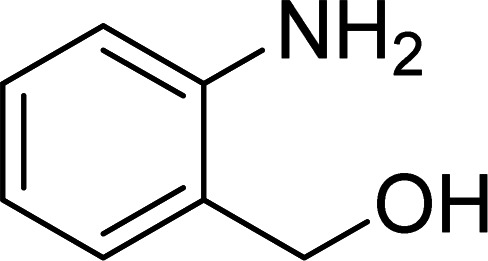	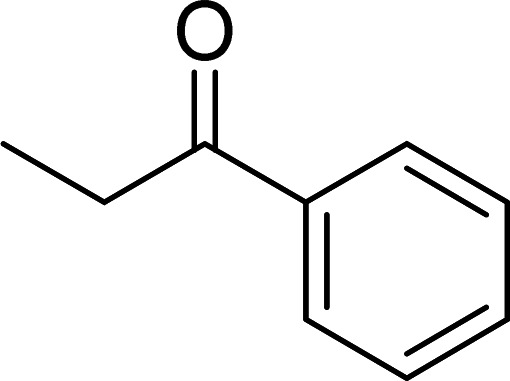	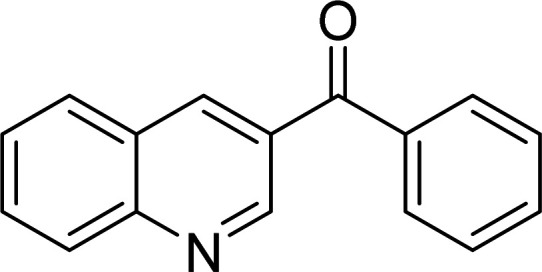	89
2	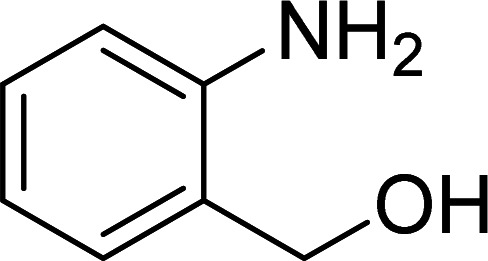	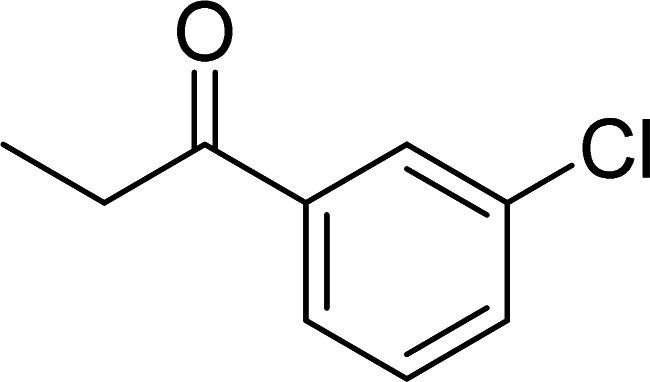	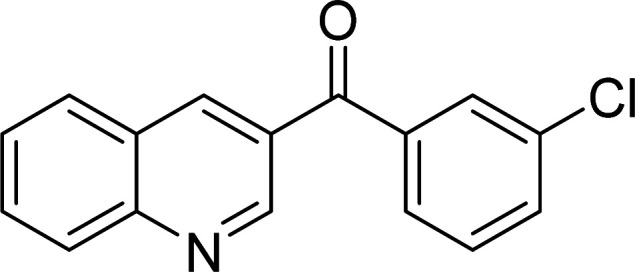	75
3	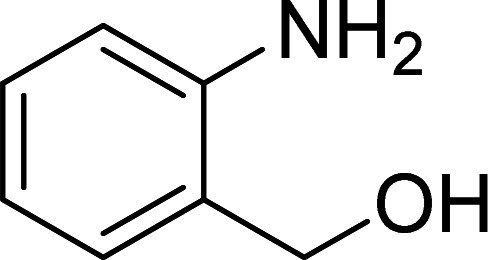	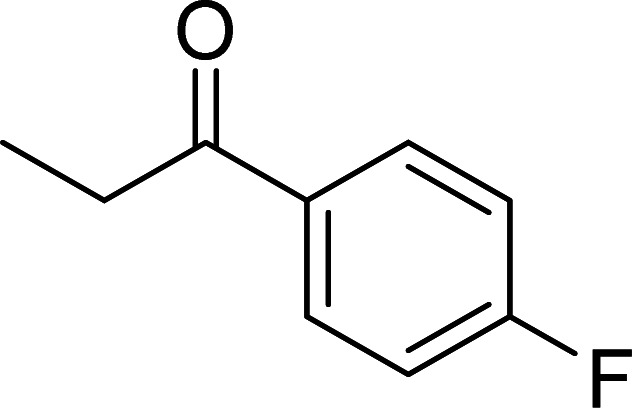	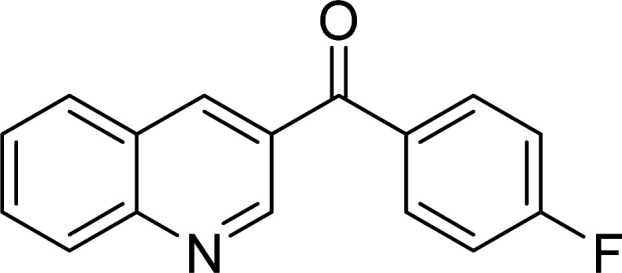	80
4	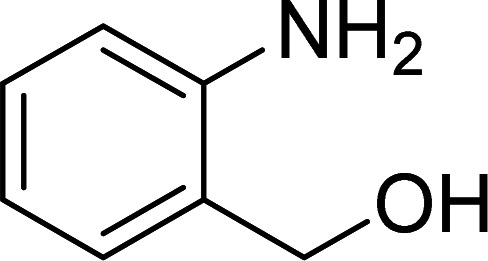	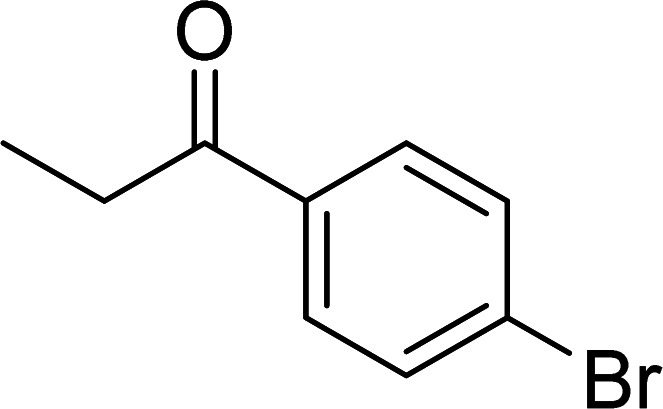	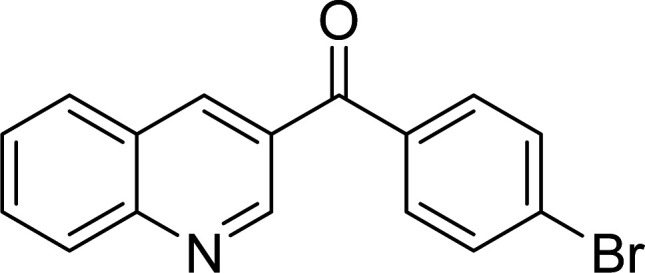	80
5	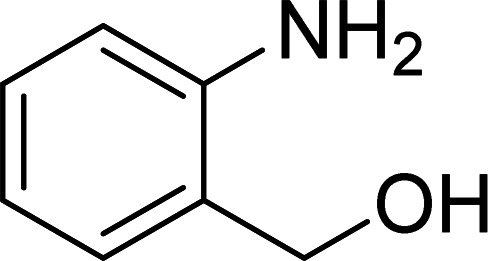	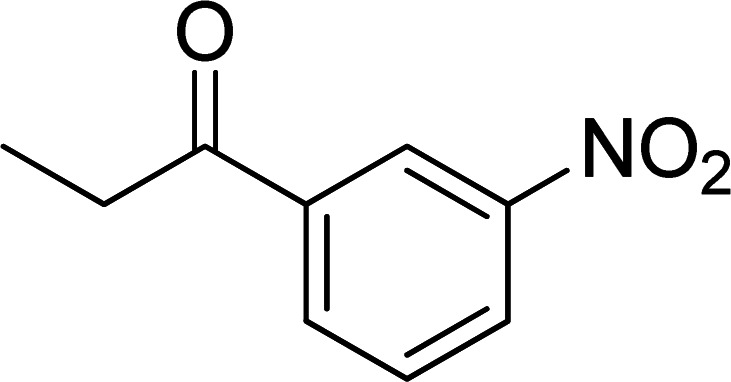	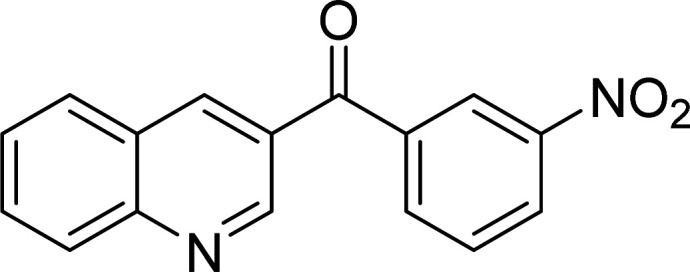	77
6	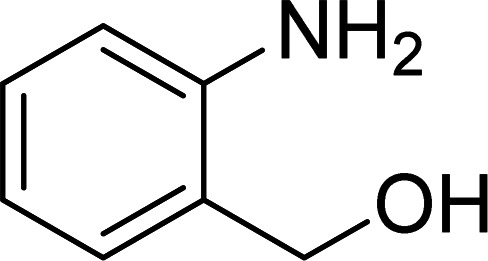	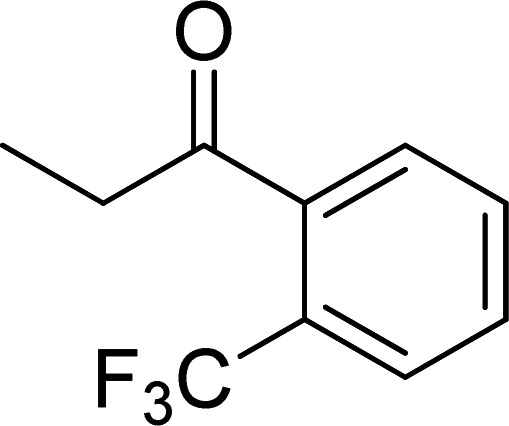	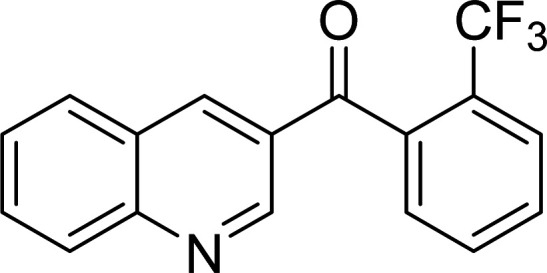	92
7	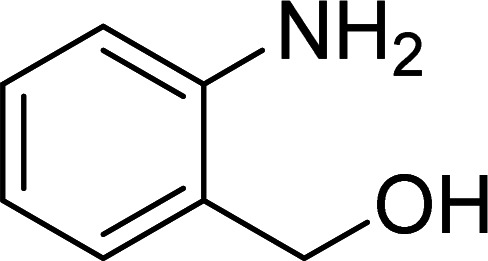	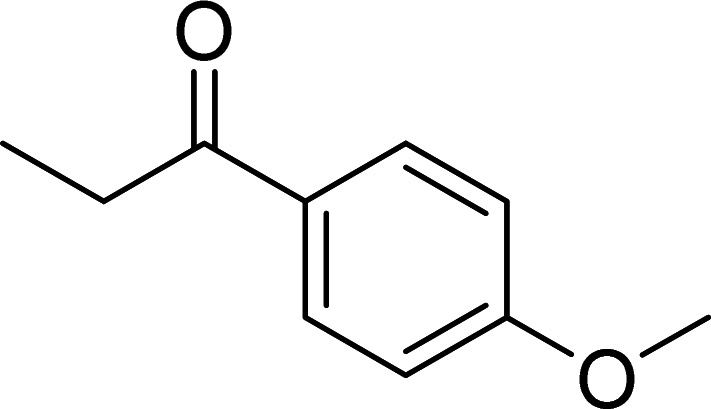	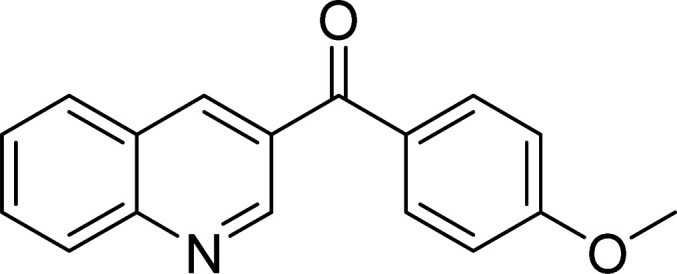	90
8	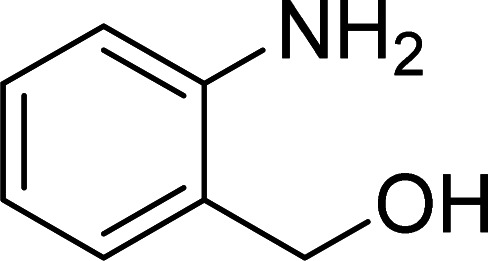	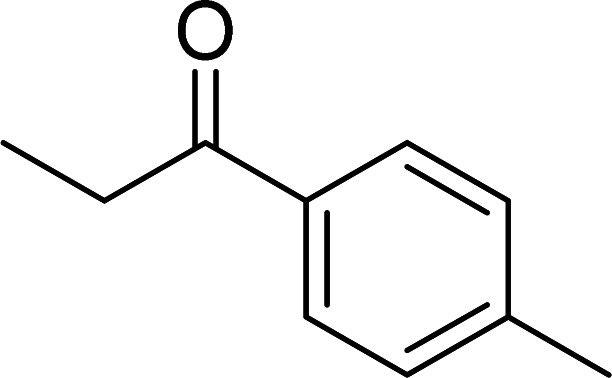	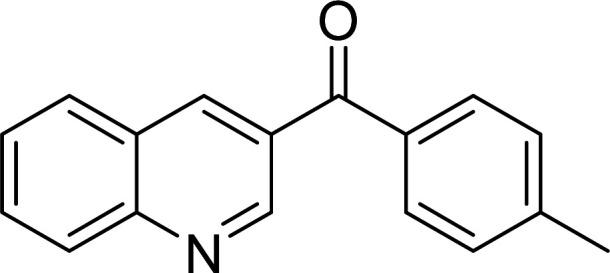	85
9	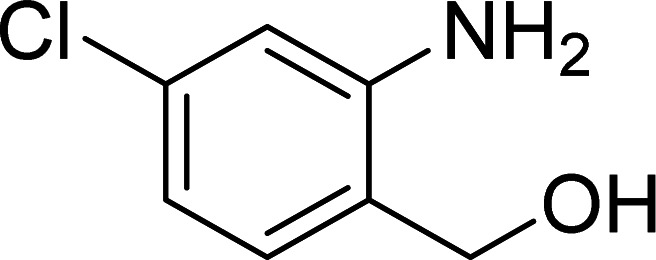	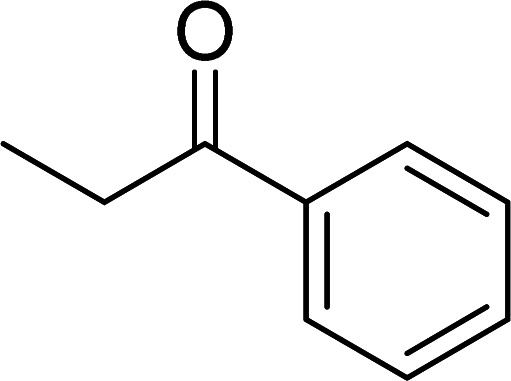	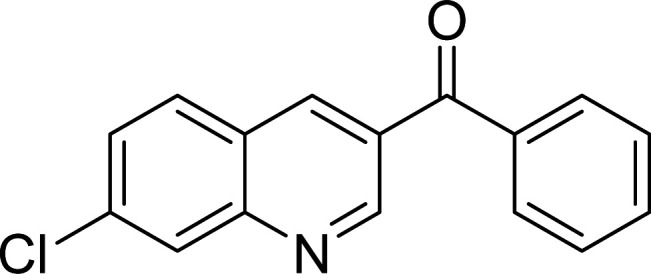	81
10	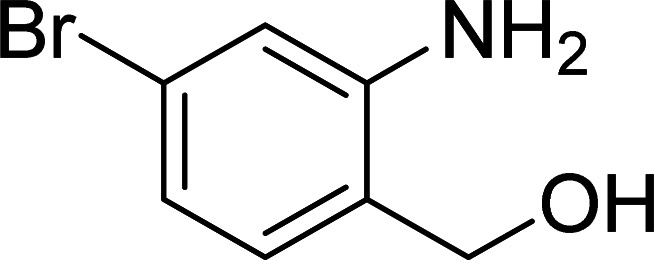	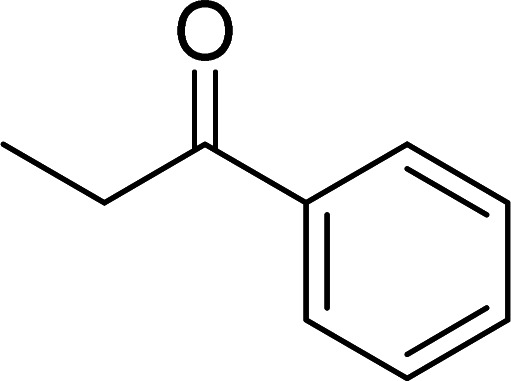	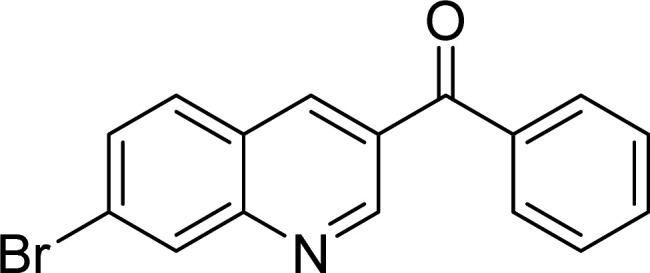	87
11	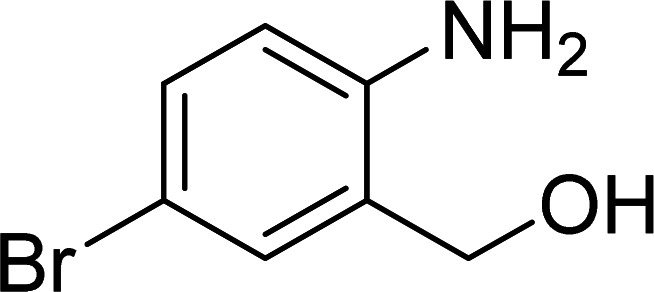	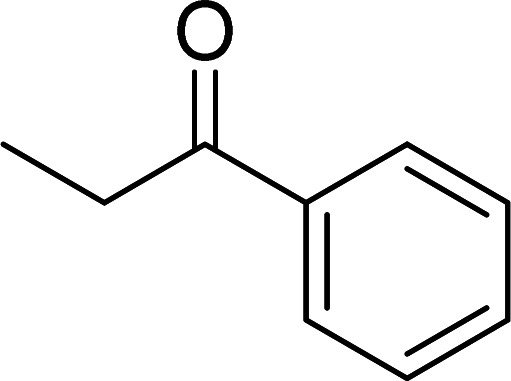	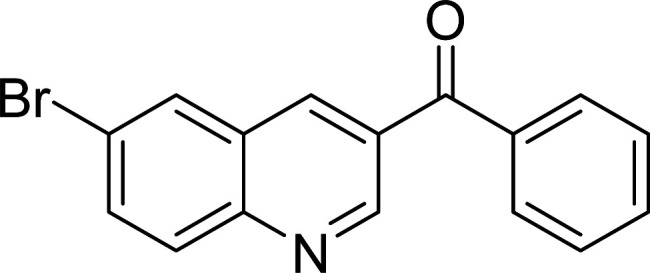	85
12	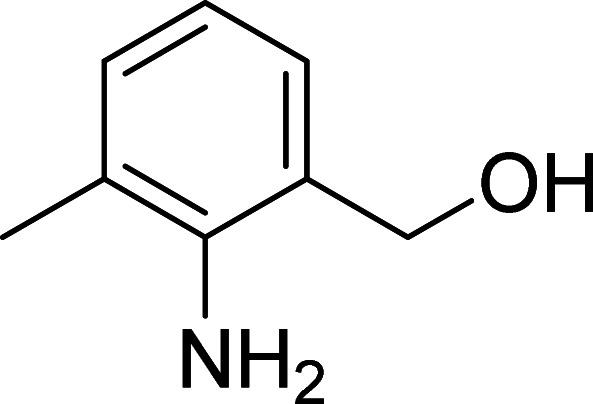	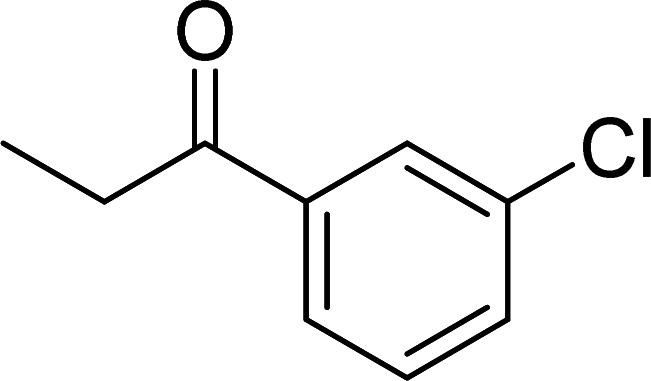	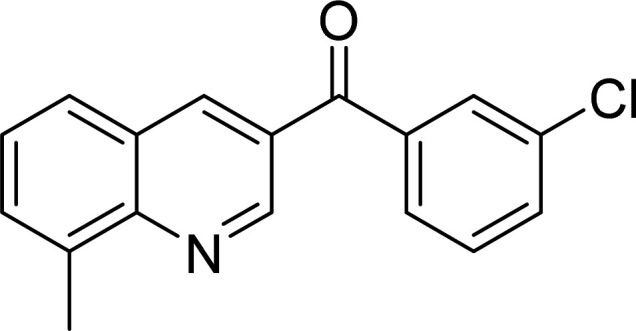	65
13	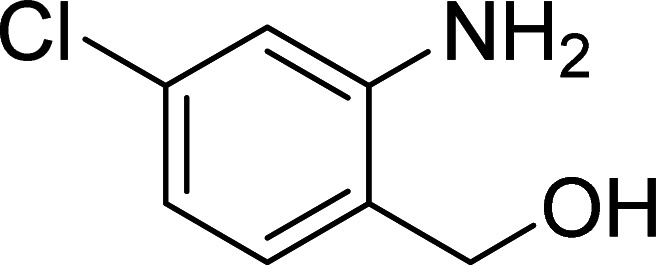	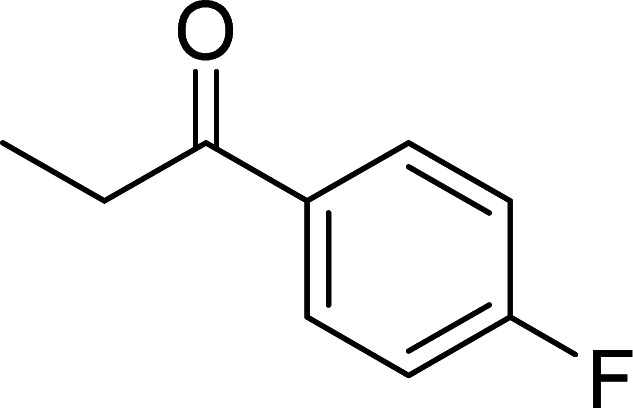	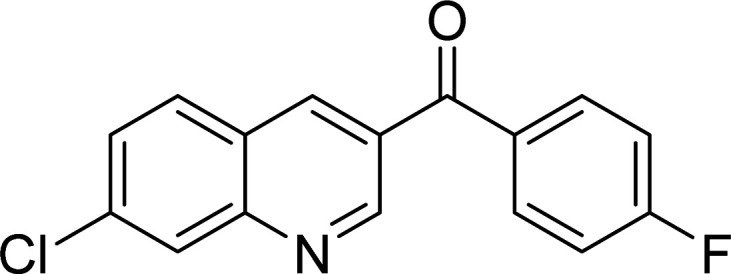	92
14	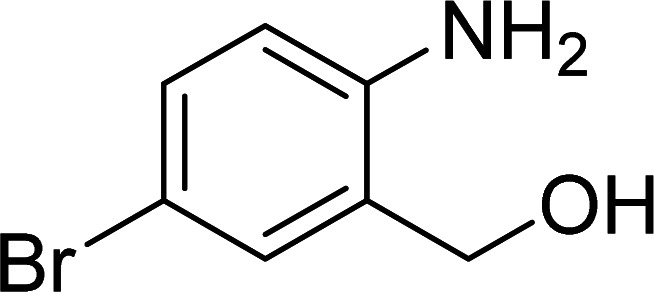	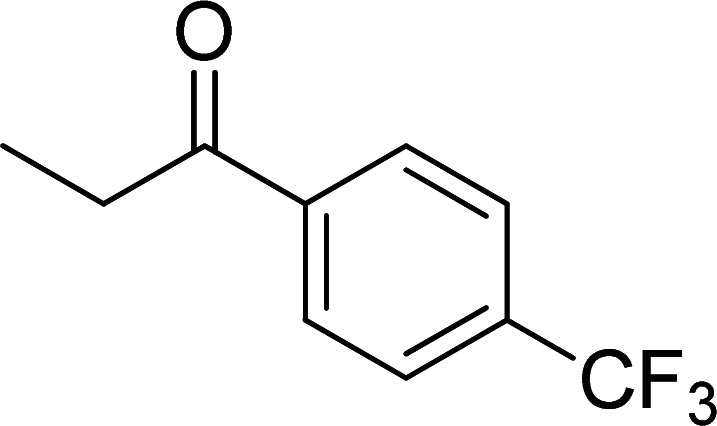	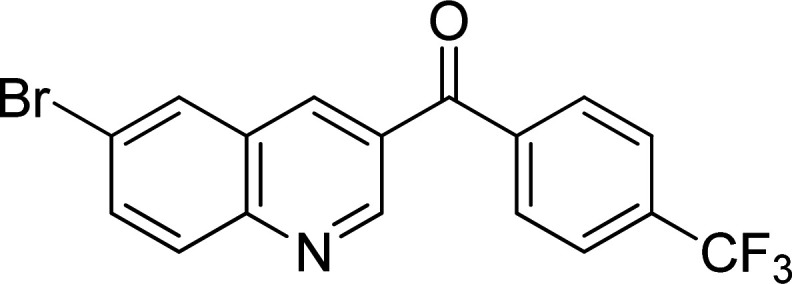	83
15	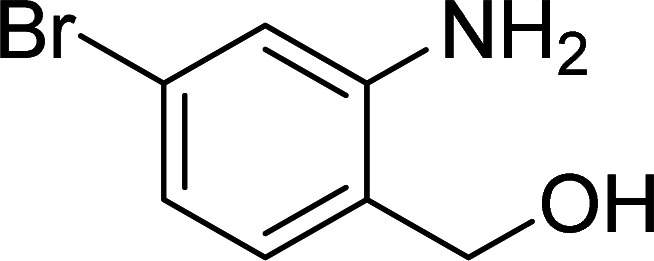	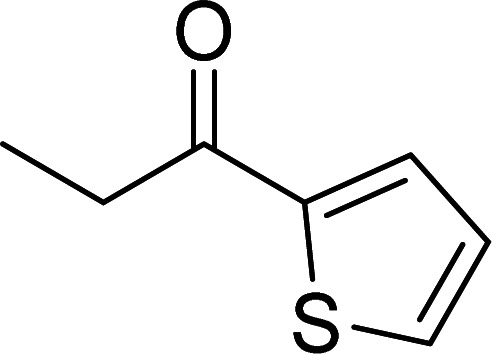	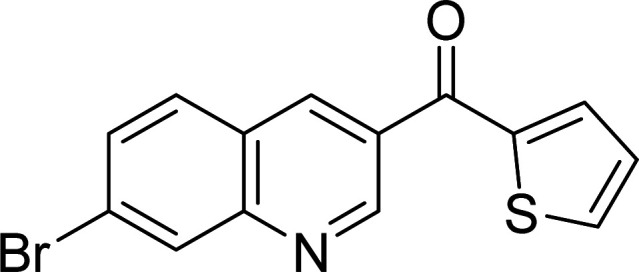	87
16	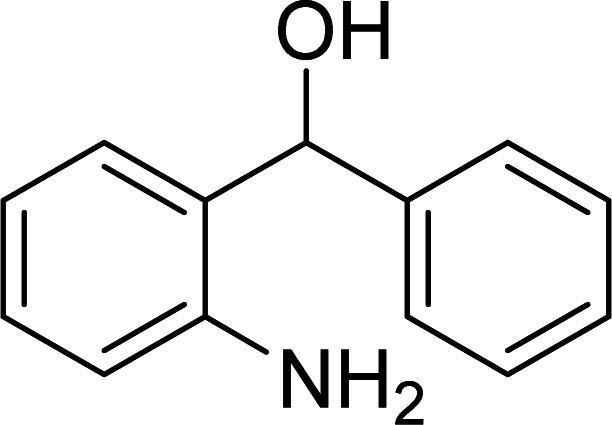	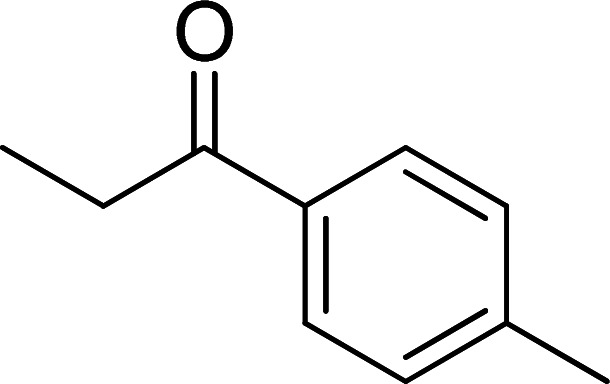	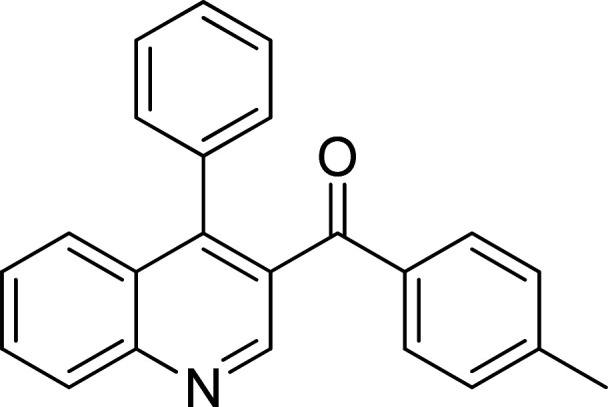	63

a Reaction conditions: 2-aminobenzylalcohol (0.2 mmol); propiophenone (0.4 mmol); Cu_2_(OBA)_2_(BPY) (10 mol%); pyridine (0.3 mmol); TEMPO (0.4 mmol); DMF (0.5 mL); 16 h.

b Isolated yield.

## 4. Conclusions

Metal–organic framework Cu_2_(OBA)_2_(BPY) was synthesized from copper(ii) nitrate trihydrate, 4,4′-oxybis(benzoic) acid, and 4,4′-bipyridine. The Cu-MOF was utilized as a heterogeneous catalyst for the synthesis of 3-aroylquinolines *via* one-pot domino reactions of 2-aminobenzylalcohols with propiophenones. The transformation was significantly controlled by the nature of oxidant, and TEMPO emerged as the only effective oxidant for the formation of 3-aroylquinolines. The ligand also displayed a noticeable impact on the reaction, and pyridine should be the best candidate. The Cu_2_(OBA)_2_(BPY) was more active towards the one-pot domino reaction than a series of conventional transition metal salts, as well as nano oxide and MOF-based catalysts. Leaching studies verified that the one-pot domino reaction utilizing the Cu_2_(OBA)_2_(BPY) catalyst proceeded under heterogeneous catalysis conditions. It was possible to reuse the copper-based framework catalyst for the synthesis of 3-aroylquinolines without an appreciable decline in catalytic performance. Utilizing 2-aminobenzylalcohols for the synthesis of 3-acylquinolines *via* one-pot domino reactions was not previously mentioned in the literature, and this protocol would be complementary to previous strategies for the synthesis of these valuable heterocycles.

## Conflicts of interest

There are no conflicts to declare.

## Supplementary Material

RA-008-C8RA05459B-s001
