# Supplementary material for: Copper-catalyzed one-pot domino reactions via C–H bond activation: synthesis of 3-aroylquinolines from 2-aminobenzylalcohols and propiophenones under metal–organic framework catalysis
Source: RSC Adv. 2018 Sep 7;8(55):31455–64. doi: 10.1039/c8ra05459b (PMC9085622; doi:10.1039/c8ra05459b)
Supplement: RA-008-C8RA05459B-s001 [file RA-008-C8RA05459B-s001.pdf]

Electronic Supplementary Material (ESI) for RSC Advances.
This journal is © The Royal Society of Chemistry 2018

# Copper-catalyzed one-pot domino reactions via C-H bond activation: Synthesis of 3-aroylquinolines from 2-aminobenzylalcohols and propiophenones under metal-organic framework catalysis

Ha V. Dang, Hoang T. B. Le, Loan T. B. Tran, Hiep Q. Ha, Ha V. Le, Nam T. S. Phan*

Faculty of Chemical Engineering, HCMC University of Technology, VNU-HCM,

268 Ly Thuong Kiet, District 10, Ho Chi Minh City, Viet Nam

*Email: ptsnam@hcmut.edu.vn

Ph: (+84 8) 38647256 ext. 5681 Fx: (+84 8) 38637504

*Materials and instrumentation*

All reagents and starting materials were obtained commercially from Sigma-Aldrich and Merck, and were used as received without any further purification unless otherwise noted. Nitrogen physisorption measurements were conducted using a Micromeritics 2020 volumetric adsorption analyzer system. Samples were pretreated by heating under vacuum at 150 $^{o}$C for 3 h. A Netzsch Thermoanalyzer STA 409 was used for thermogravimetric analysis (TGA) with a heating rate of 10 $^{o}$C/min under a nitrogen atmosphere. X-ray powder diffraction (XRD) patterns were recorded using a Cu Kα radiation source on a D8 Advance Bruker powder diffractometer. Scanning electron microscopy studies were conducted on a S4800 Scanning Electron Microscope (SEM). Transmission electron microscopy studies were performed using a JEOL JEM 1400 Transmission Electron Microscope (TEM) at 80 kV. The $Fe_3O(BPDC)_3$ sample was dispersed on holey carbon grids for TEM observation. Elemental analysis with atomic absorption spectrophotometry

(AAS) was performed on an AA-6800 Shimadzu. Fourier transform infrared (FT-IR) spectra were obtained on a Nicolet 6700 instrument, with samples being dispersed on potassium bromide pallets.

Gas chromatographic (GC) analyses were performed using a Shimadzu GC 2010-Plus equipped with a flame ionization detector (FID) and an SPB-5 column (length = 30 m, inner diameter = 0.25 mm, and film thickness = 0.25 μm). The temperature program for GC analysis held samples at 120 $^{o}$C for 0.5 min; heated them from 120 to 130 $^{o}$C at 40 $^{o}$C/min; held them at 130 $^{o}$C for 1 min; heated them from 130 to 280 $^{o}$C at 40 $^{o}$C/min; and finally held them at 280 $^{o}$C for 1.5 min.. Inlet and detector temperatures were set constant at 280 $^{o}$C. The GC yield was calculated using diphenyl ether as the internal standard. GC-MS analyses were analyzed on a Shimadzu GCMS-QP2010Ultra with a ZB-5MS column (length = 30 m, inner diameter = 0.25 mm, and film thickness = 0.25 μm). The temperature program for GC-MS analysis held samples at 50 $^{o}$C for 2 min; heated samples from 50 to 280$^{o}$C at 10 $^{o}$C/min and held them at 280 $^{o}$C for 10 min. Inlet temperature was set constant at 280 $^{o}$C. MS spectra were compared with the spectra gathered in the NIST library. The $^{1}$H NMR and $^{13}$C NMR were recorded on Bruker AV 500 spectrometers using residual solvent peak as a reference.

*Synthesis of $Cu_2(OBA)_2(BPY)$ metal-organic framework*

In a typical synthesis, a solution of 4,4′-oxybis(benzoic) acid ($H_2OBA$) (0.258 g, 1 mmol) in DMF (DMF = N,N'-dimethylformamide; 3 mL) and water (1 mL) was prepared. Simultaneously, a solution of copper (II) nitrate trihydrate ($Cu(NO_3)_2.3H_2O$) (0.242 g, 1

mmol) in DMF (5 mL) and water (2 mL), and a solution of 4,4′-bipyridine (BPY) (0.078 g, 0.5 mmol) in DMF (3 mL), respectively, were prepared. These three solutions were added to a round-bottomed flash, and the mixture was stirred for 30 min to achieve a clear solution. Consequently, the solution was equally distributed to three 10-mL pressurized vials. The vials were tightly covered and heated at 85$^{o}$C in an isothermal oven for 48 h. Green crystals were produced on the wall of the vials during the experiment. After cooling the vials to ambient temperature, the crystals were collected by decantation, and washed with in DMF (3 x 20 mL). Subsequently, the product was dried under vacuum on a Schlenk line at 150 $^{o}$C for 6h, obtaining 0.284 g of $Cu_2(OBA)_2(BPY)$ as green light crystals (71% yield, with regard to copper (II) nitrate trihydrate).

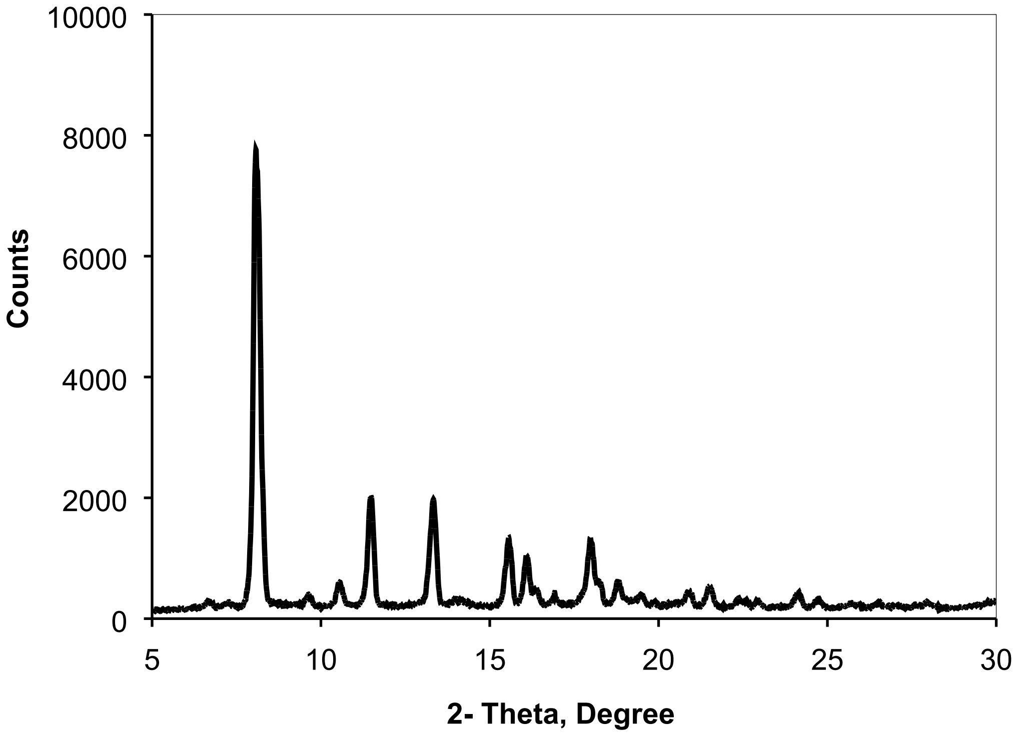


Fig. S1. X-ray powder diffractograms of $Cu_2(OBA)_2(BPY)$.

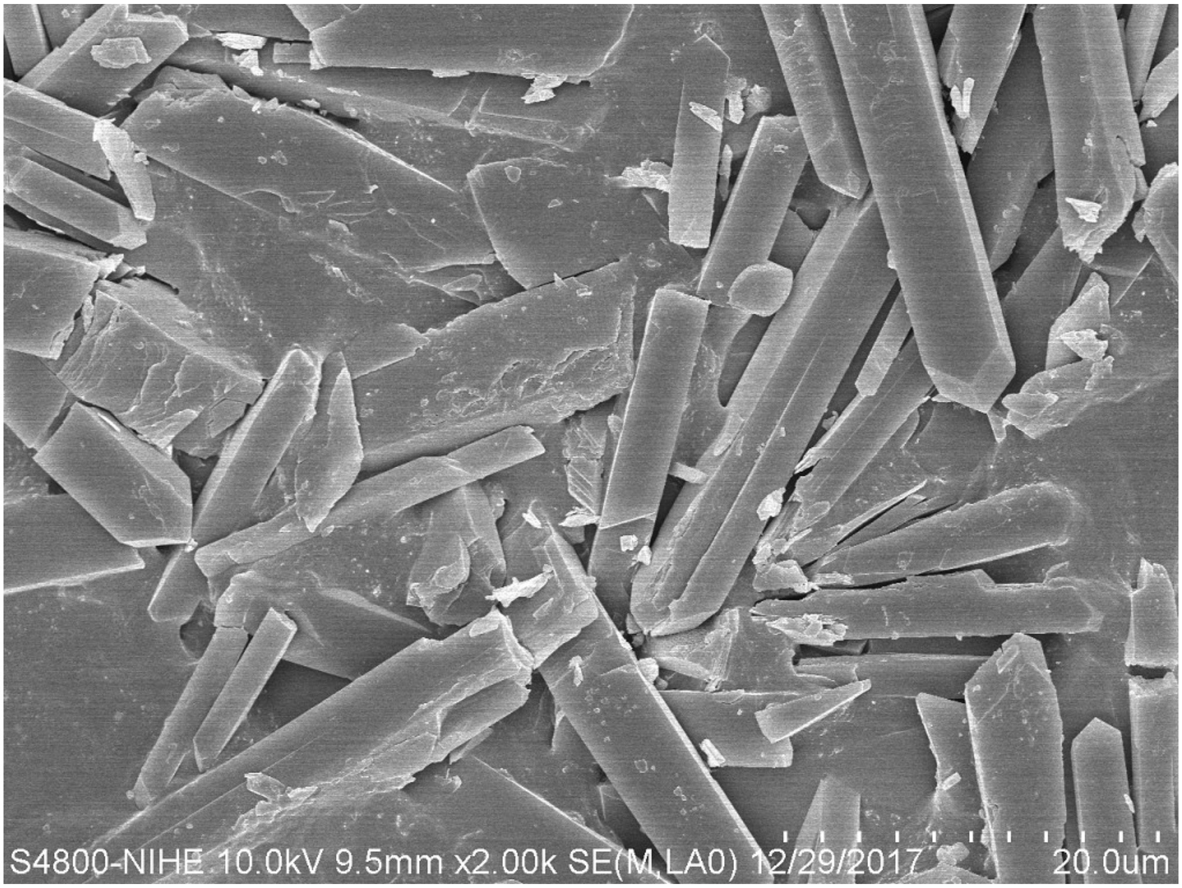


Fig. S2. SEM micrograph of $Cu_2(OBA)_2(BPY)$.

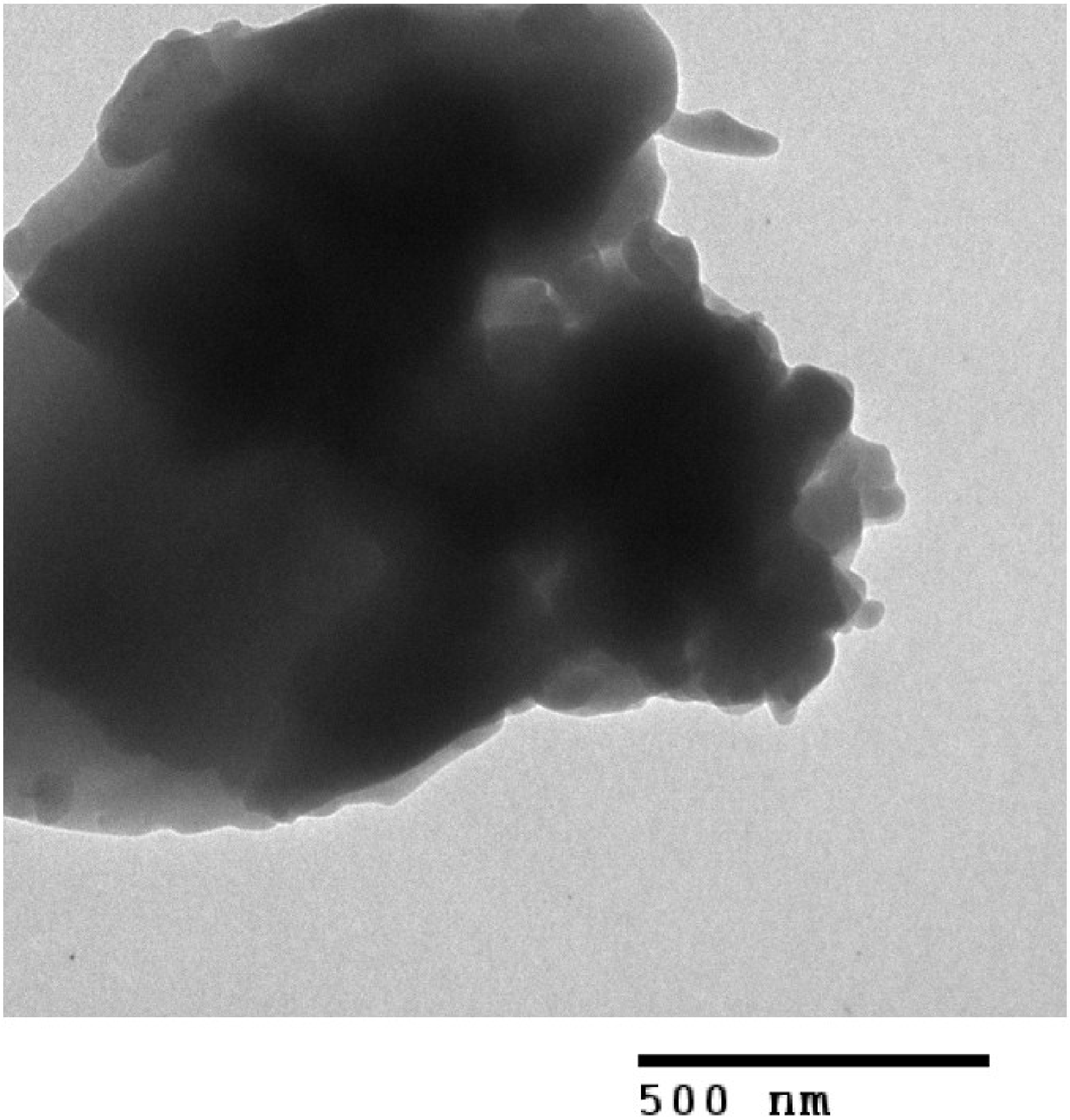


Fig. S3. TEM micrograph of $Cu_2(OBA)_2(BPY)$.

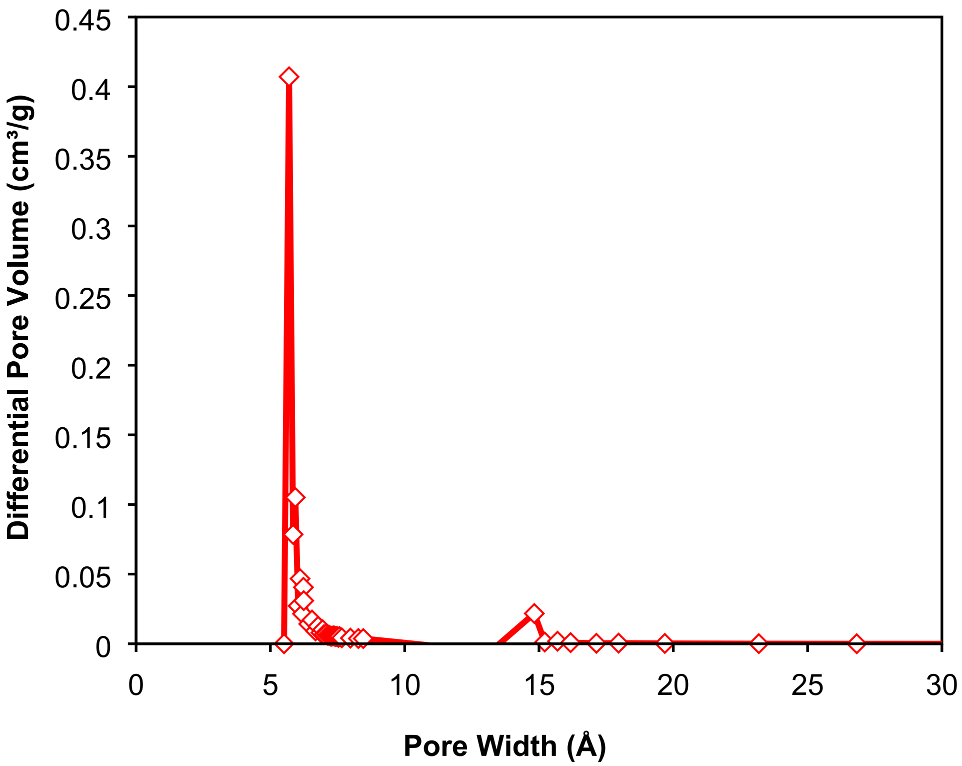


Fig. S4. Pore size distribution of $Cu_2(OBA)_2(BPY)$.

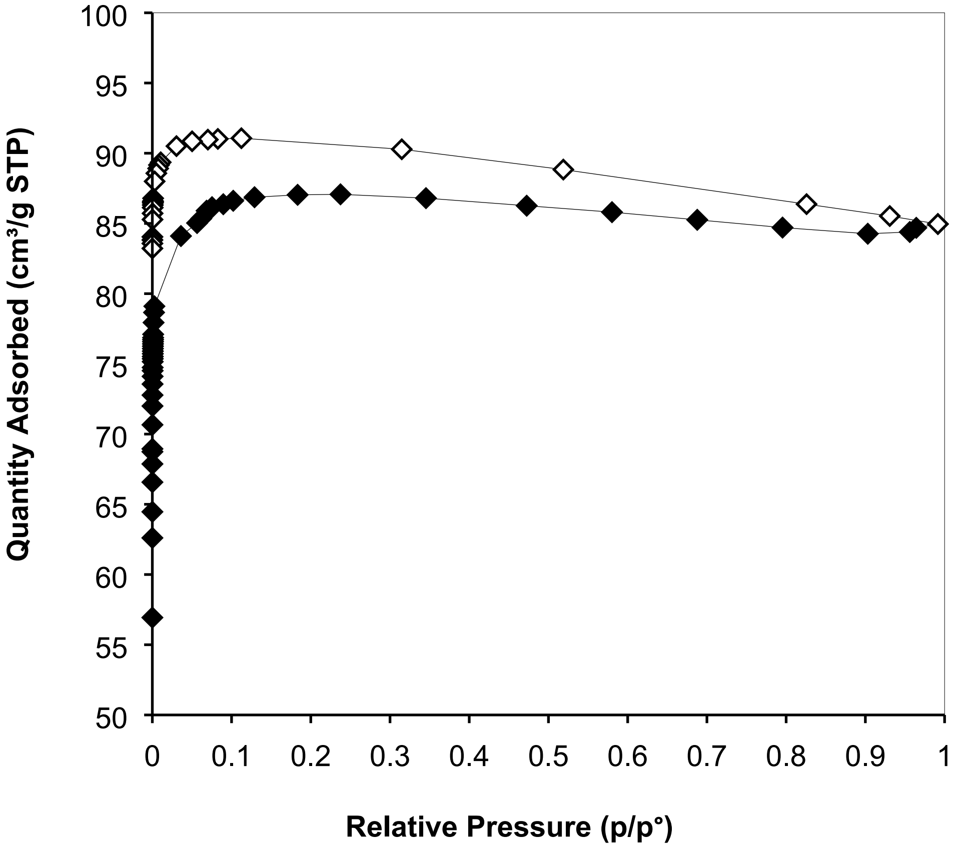


Fig. S5. Nitrogen adsorption/desorption isotherm of the $Cu_2(OBA)_2(BPY)$. Adsorption data are shown as closed triangles and desorption data as open triangles.

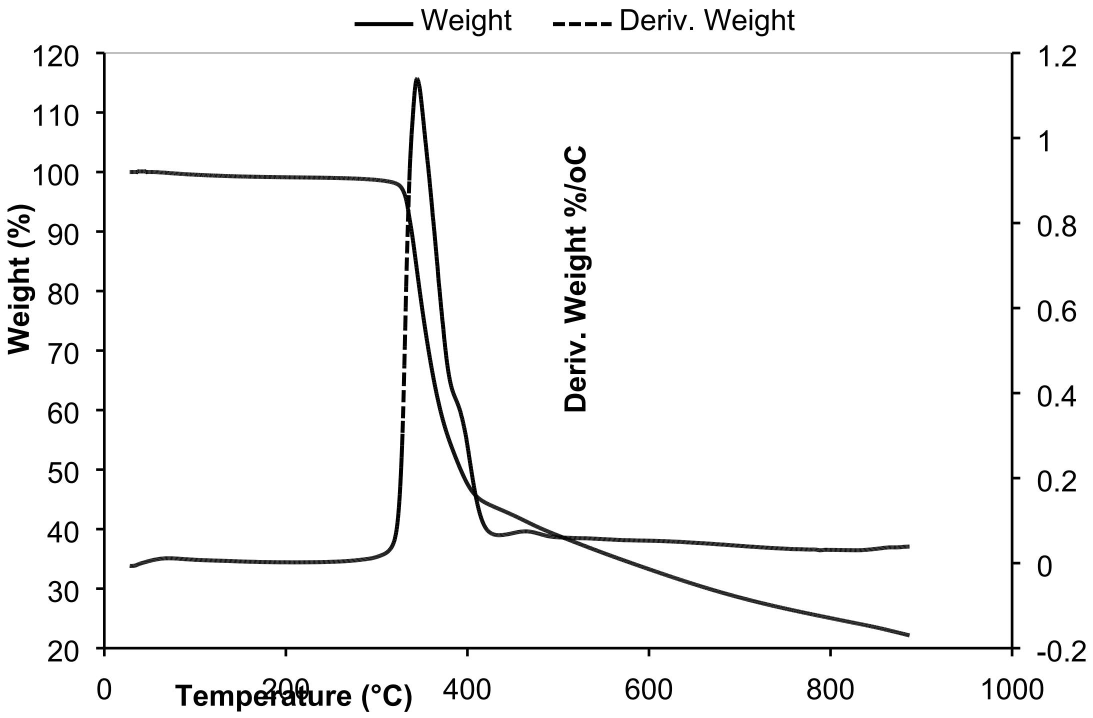


Fig. S6. TGA analysis of $Cu_2(OBA)_2(BPY)$.

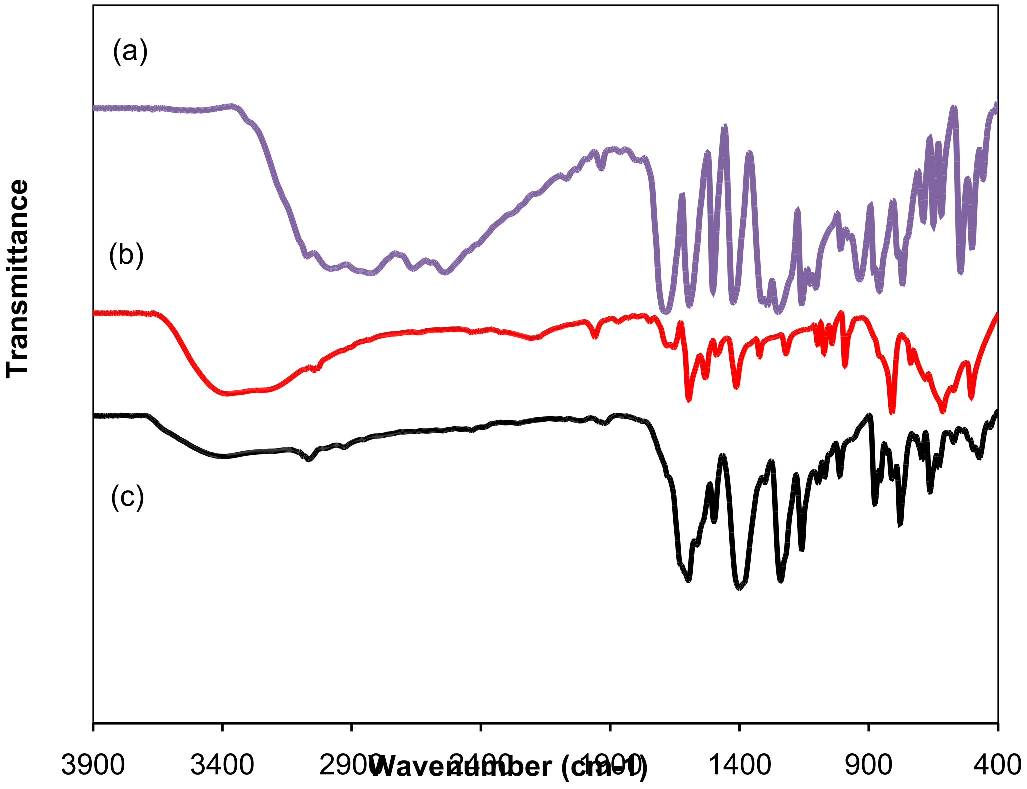


Fig. S7. FT-IR spectra of 4,4′-oxybis(benzoic) acid (a), 4,4′-bipyridine (b), and $Cu_2(OBA)_2(BPY)$ (c).

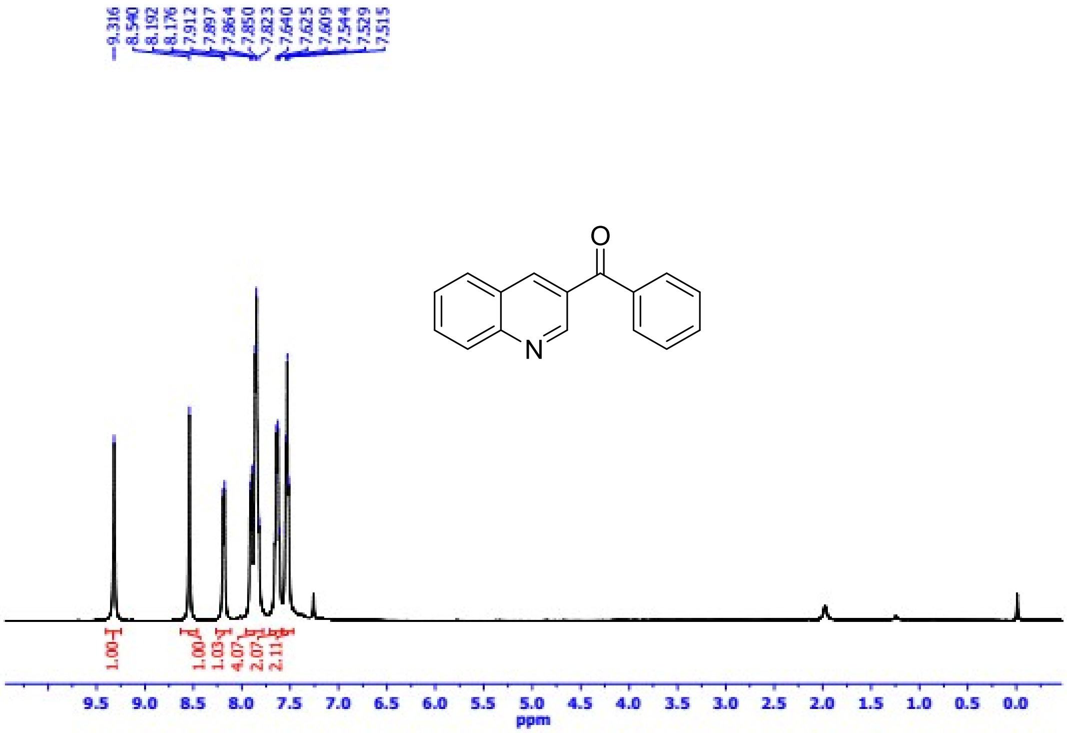


Fig. S8. $^{1}$H-NMR spectra of phenyl(quinolin-3-yl)methanone.

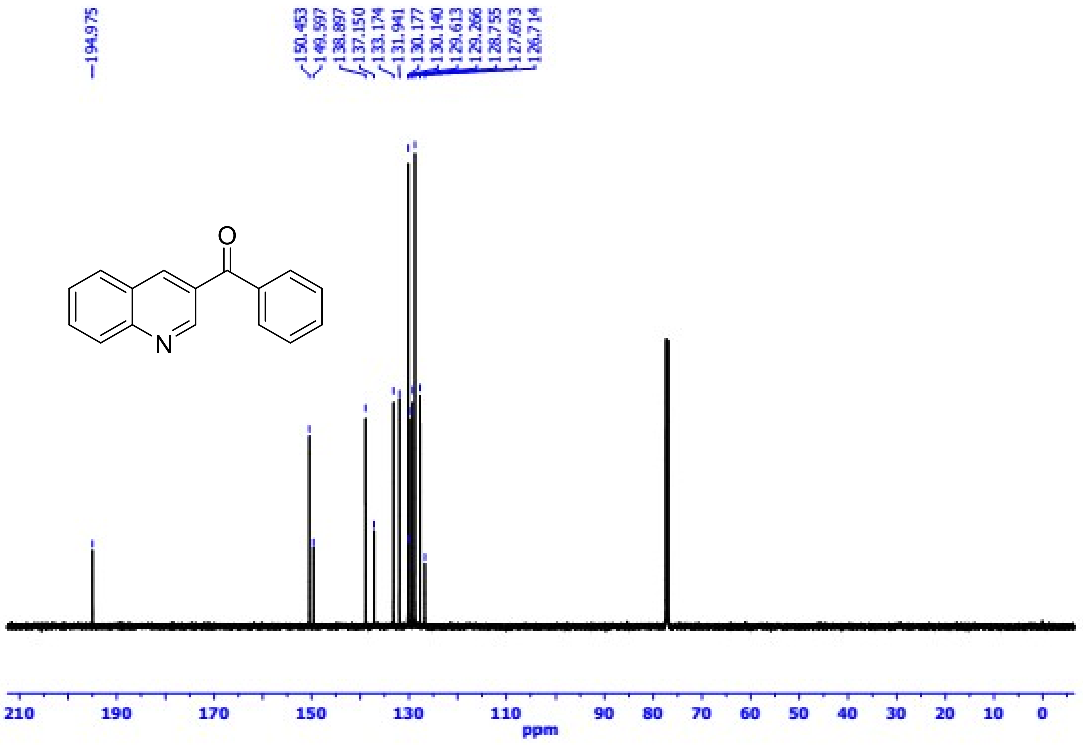


Fig. S9. $^{13}$C-NMR spectra of phenyl(quinolin-3-yl)methanone.

**Characterization data for phenyl(quinolin-3-yl)methanone.**

Prepared as shown in the general experimental procedure and purified on silica gel (hexane/ ethyl acetate =4 /1): white solid, 89% yield. $^{1}$H NMR (500 MHz, $CDCl_3$) δ 9.32 (s, 1H), 8.54 (s, 1H), 8.18 (d, *J* = 8.3 Hz, 1H), 7.96 – 7.78 (m, 4H), 7.71 – 7.59 (m, 2H), 7.53 (t, *J* = 7.2 Hz, 2H). $^{13}$C NMR (126 MHz, $CDCl_3$) δ 195.0, 150.5, 149.6, 138.9, 137.2, 133.2, 131.9, 130.2, 130.1, 129.6, 129.3, 128.8, 127.7, 126.7.

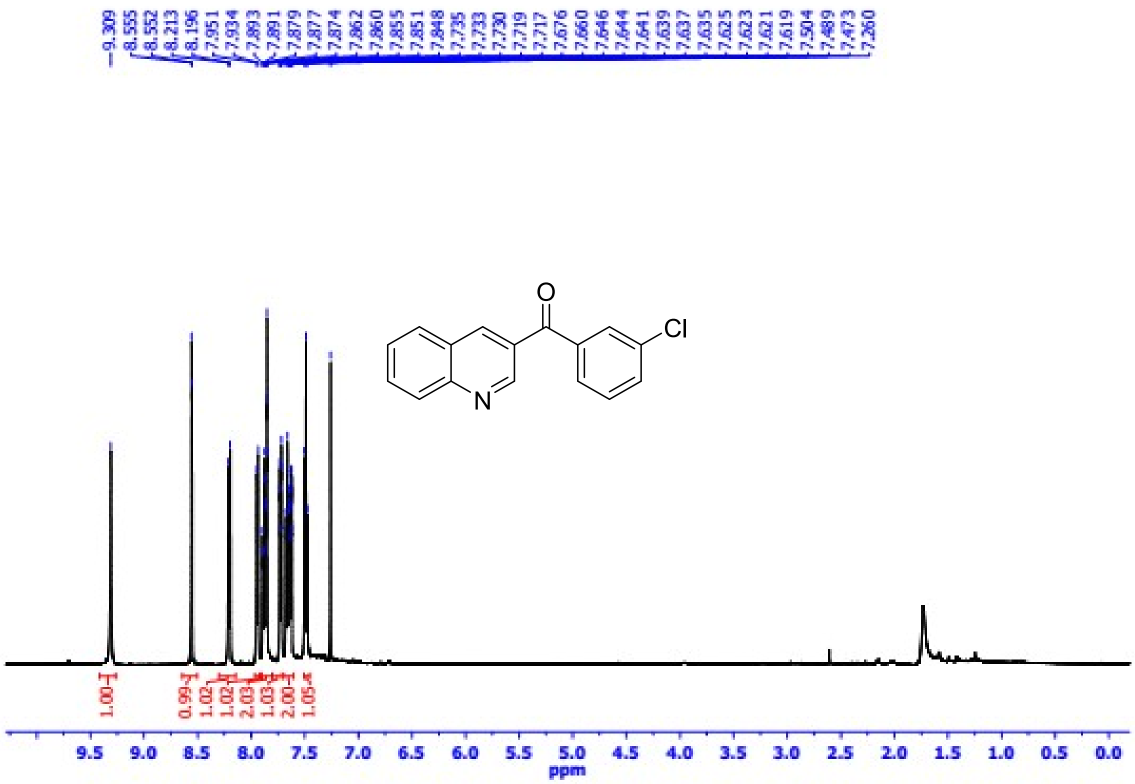


Fig. S10. $^{1}$H-NMR spectra of (3-chlorophenyl)(quinolin-3-yl)methanone.

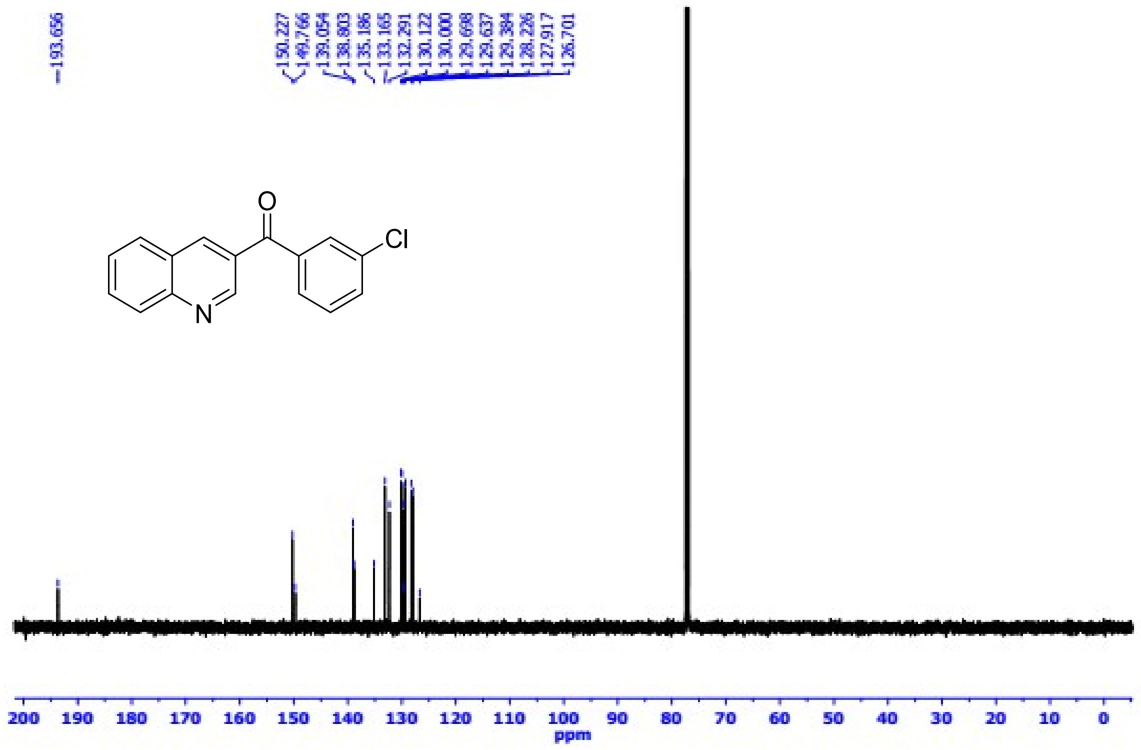

Fig. S11. $^{13}$C-NMR spectra of (3-chlorophenyl)(quinolin-3-yl)methanone.

**Characterization data for (3-chlorophenyl)(quinolin-3-yl)methanone.**

Prepared as shown in the general experimental procedure and purified on silica gel (hexane/ ethyl acetate =4 /1): yellow solid, 75% yield. $^{1}$H NMR (500 MHz, $CDCl_3$) δ 9.31 (s, 1H), 8.55 (d, *J* = 1.7 Hz, 1H), 8.20 (d, *J* = 8.5 Hz, 1H), 7.94 (d, *J* = 8.1 Hz, 1H), 7.92 – 7.80 (m, 2H), 7.80 – 7.71 (m, 1H), 7.70 – 7.61 (m, 2H), 7.49 (t, *J* = 7.9 Hz, 1H). $^{13}$C NMR (126 MHz, $CDCl_3$) δ 193.7, 150.2, 149.8, 139.1, 138.8, 135.2, 133.2, 132.3, 130.1, 130.0, 129.7, 129.6, 129.4, 128.2, 127.9, 126.7.

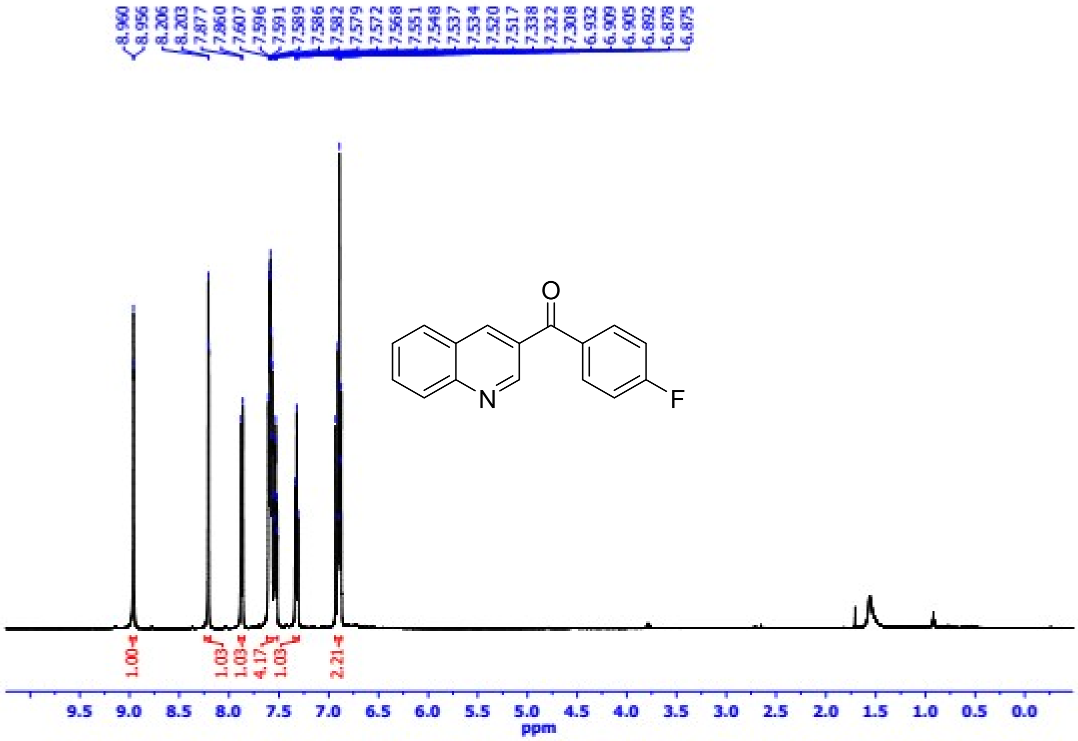


Fig. S12. $^{1}$H-NMR spectra of (4-fluorophenyl)(quinolin-3-yl)methanone.

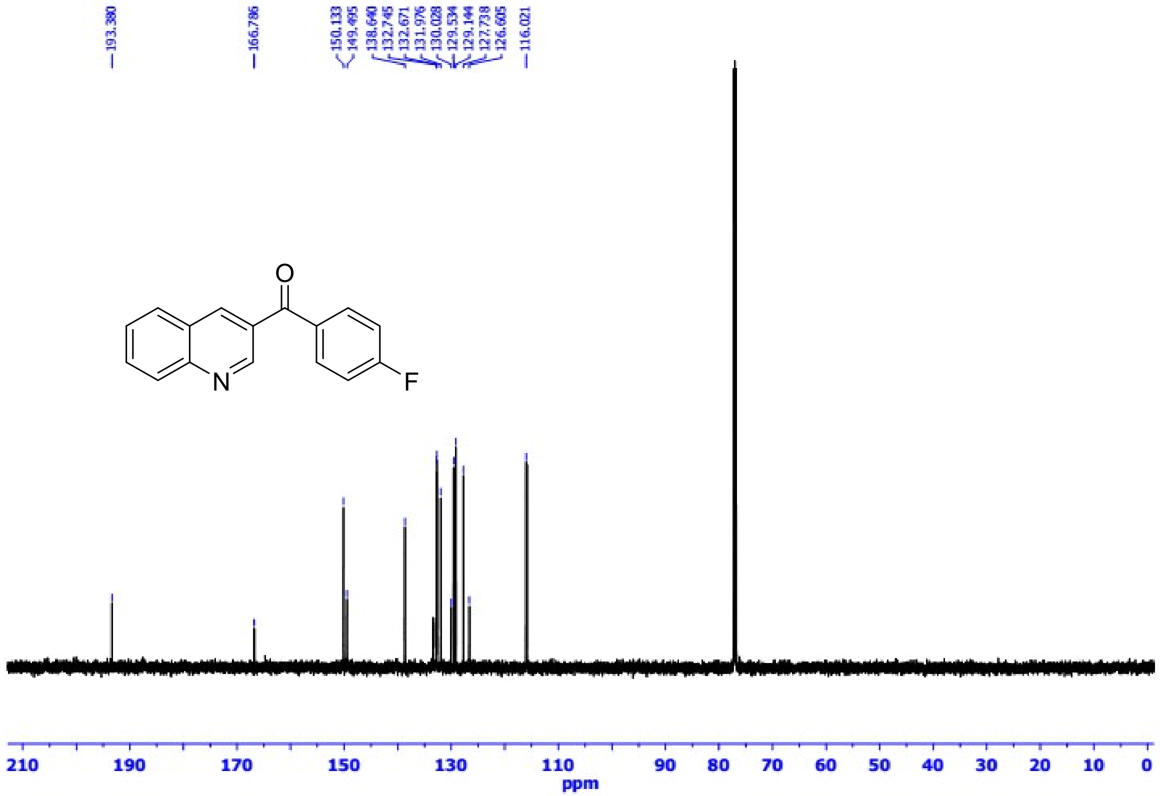


Fig. S13. $^{13}$C-NMR spectra of (4-fluorophenyl)(quinolin-3-yl)methanone.

**Characterization data for (4-fluorophenyl)(quinolin-3-yl)methanone**

Prepared as shown in the general experimental procedure and purified on silica gel (hexane/ ethyl acetate = 4/1): yellow solid, 80% yield. $^1$H NMR (500 MHz, $CDCl_3$) δ 8.96 (d, $J$ = 1.8 Hz, 1H), 8.20 (d, $J$ = 1.7 Hz, 1H), 7.87 (d, $J$ = 8.5 Hz, 1H), 7.62 – 7.51 (m, 4H), 7.32 (t, $J$ = 7.5 Hz, 1H), 6.93 – 6.87 (m, 2H). $^{13}$C NMR (126 MHz, $CDCl_3$) δ 193.4, 166.8, 150.1, 149.5, 138.6, 132.7, 132.7, 132.0, 130.0, 129.5, 129.1, 127.7, 126.6, 116.0.

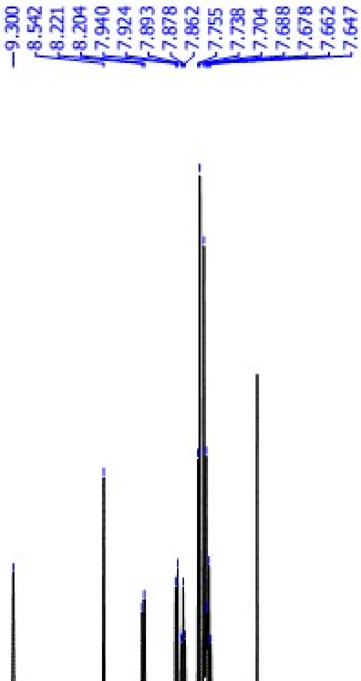

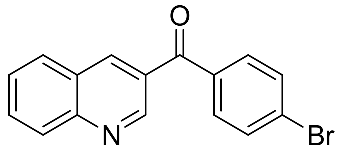


Fig. S14. $^{1}$H-NMR spectra of (4-bromophenyl)(quinolin-3-yl)methanone.

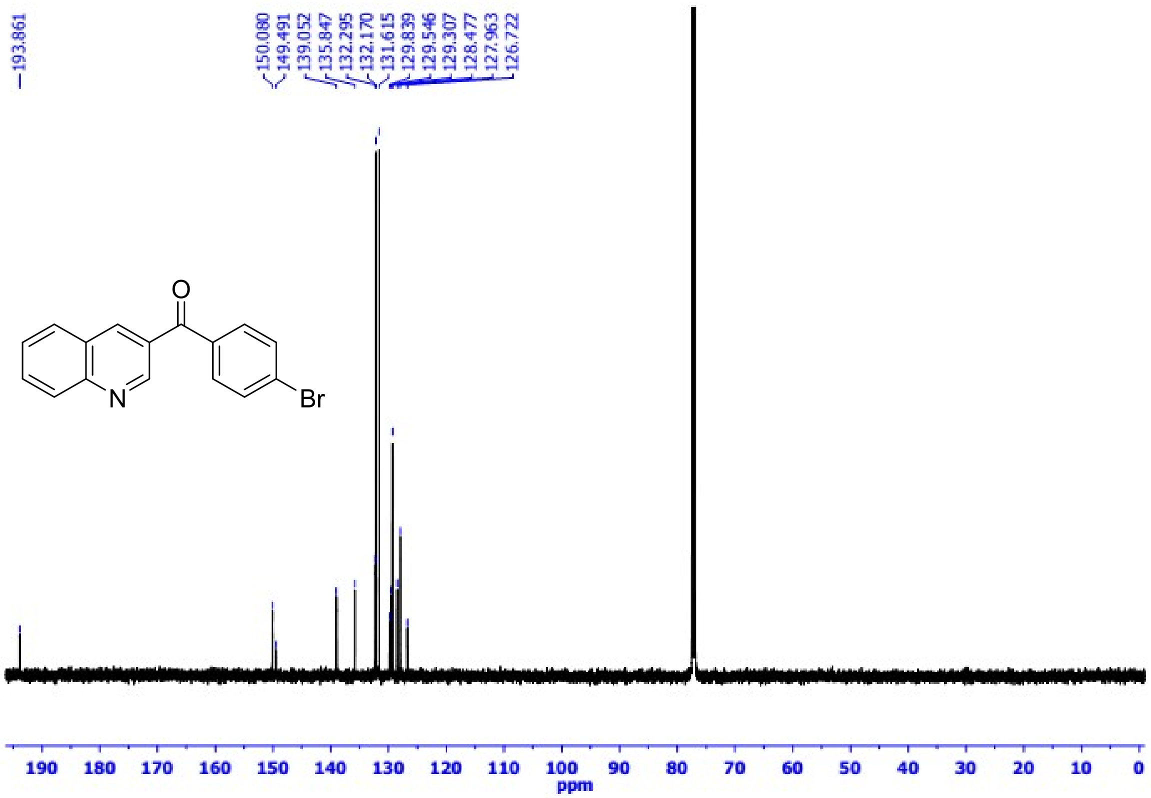


Fig. S15. $^{13}$C-NMR spectra of (4-bromophenyl)(quinolin-3-yl)methanone.

**Characterization data for (4-bromophenyl)(quinolin-3-yl)methanone.**

Prepared as shown in the general experimental procedure and purified on silica gel (hexane/ ethyl acetate =3 /2): white solid, 80% yield. $^{1}$H NMR (500 MHz, $CDCl_3$) δ 9.30 (s, 1H), 8.54 (s, 1H), 8.21 (d, $J$ = 8.4 Hz, 1H), 7.93 (d, $J$ = 8.1 Hz, 1H), 7.88 (t, $J$ = 7.7 Hz, 1H), 7.75 (d, $J$ = 8.4 Hz, 2H), 7.72 – 7.65 (m, 3H). $^{13}$C NMR (126 MHz, $CDCl_3$) δ 193.9, 150.1, 149.5, 139.0, 135.8, 132.3, 132.2, 131.6, 129.8, 129.5, 129.3, 128.5, 127.96, 126.7.

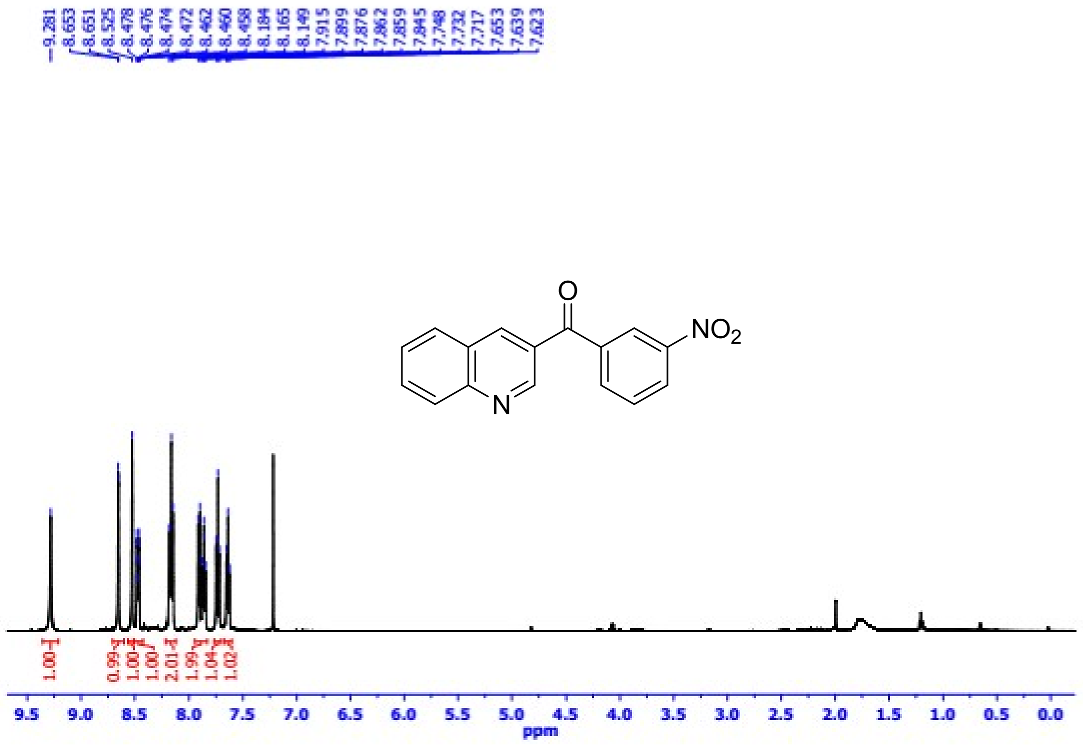

Fig. S16. $^{1}$H-NMR spectra of (3-nitrophenyl)(quinolin-3-yl)methanone

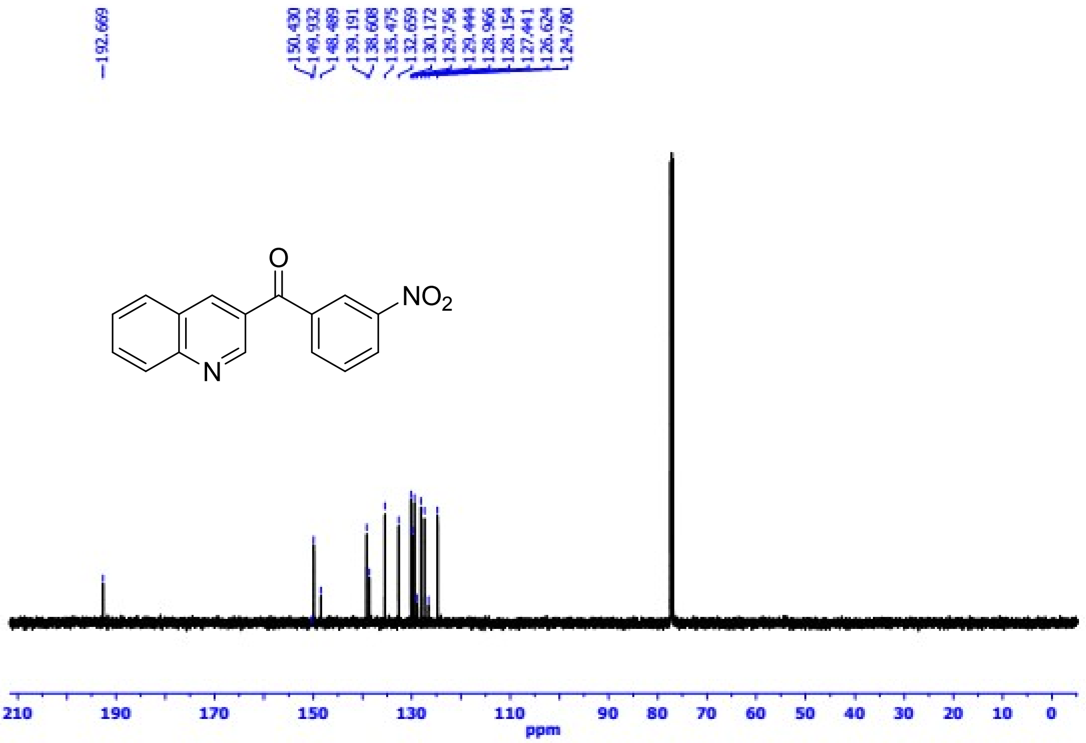


Fig. S17. $^{13}$C-NMR spectra of (3-nitrophenyl)(quinolin-3-yl)methanone.

**Characterization data for (3-nitrophenyl)(quinolin-3-yl)methanone**

Prepared as shown in the general experimental procedure and purified on silica gel (hexane/ ethyl acetate =3 /2): yellow solid, 77% yield. $^{1}$H NMR (500 MHz, $CDCl_3$) δ 9.28 (s, 1H), 8.65 (d, $J$ = 1.2 Hz, 1H), 8.52 (s, 1H), 8.50 – 8.42 (m, 1H), 8.17 (t, $J$ = 8.7 Hz, 2H), 7.95 – 7.83 (m, 2H), 7.73 (t, $J$ = 7.9 Hz, 1H), 7.64 (t, $J$ = 7.5 Hz, 1H). $^{13}$C NMR (126 MHz, $CDCl_3$) δ 192.7, 150.4, 149.9, 148.5, 139.2, 138.6, 135.5, 132.7, 130.2, 129.8, 129.4, 129.0, 128.2, 127.4, 126.6, 124.8.

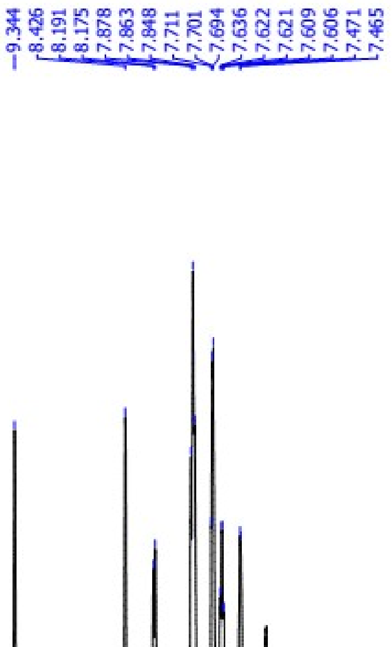

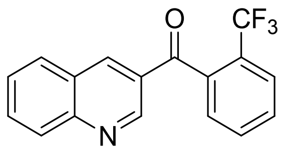


Fig. S18. $^{1}$H-NMR spectra of quinolin-3-yl(2(trifluoromethyl)phenyl)methanone.

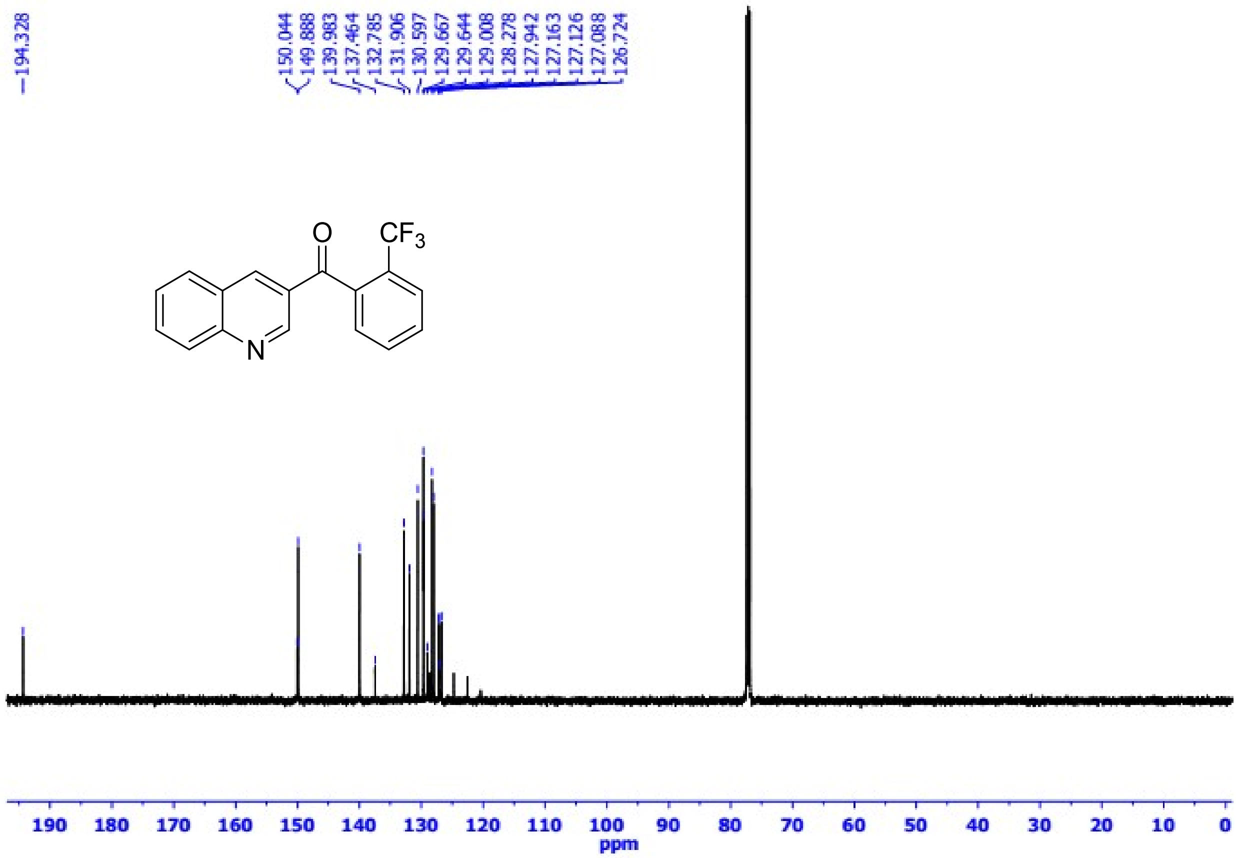


Fig. S19. $^{13}$C-NMR spectra of quinolin-3-yl(2 (trifluoromethyl)phenyl) methanone.

**Characterization data for quinolin-3-yl(2-(trifluoromethyl)phenyl)methanone.**

Prepared as shown in the general experimental procedure and purified on silica gel (hexane/ ethyl acetate =3 /2): white solid, 92% yield. $^{1}$H NMR (500 MHz, $CDCl_3$) δ 9.34 (s, 1H), 8.43 (s, 1H), 8.18 (d, $J$ = 8.1 Hz, 1H), 7.86 (t, $J$ = 7.6 Hz, 3H), 7.72 – 7.68 (m, 2H), 7.64 – 7.60 (m, 1H), 7.47 (d, $J$ = 3.1 Hz, 1H). $^{13}$C NMR (126 MHz, $CDCl_3$) δ 194.3, 150.0, 149.9, 140.0, 137.5, 132.8, 131.9, 130.6, 129.7, 129.6, 129.0, 128.3, 127.9, 127.2, 127.1, 127.1, 126.7.

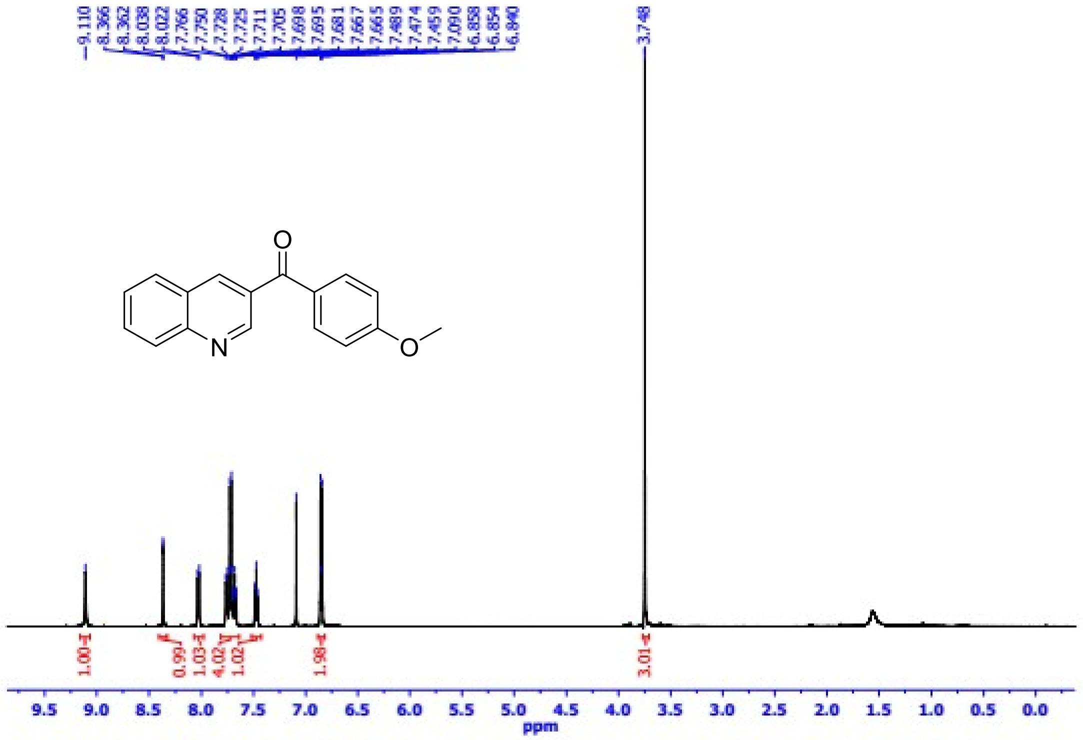


Fig. S20. $^{1}$H-NMR spectra of (4-methoxyphenyl)(quinolin-3-yl)methanone.

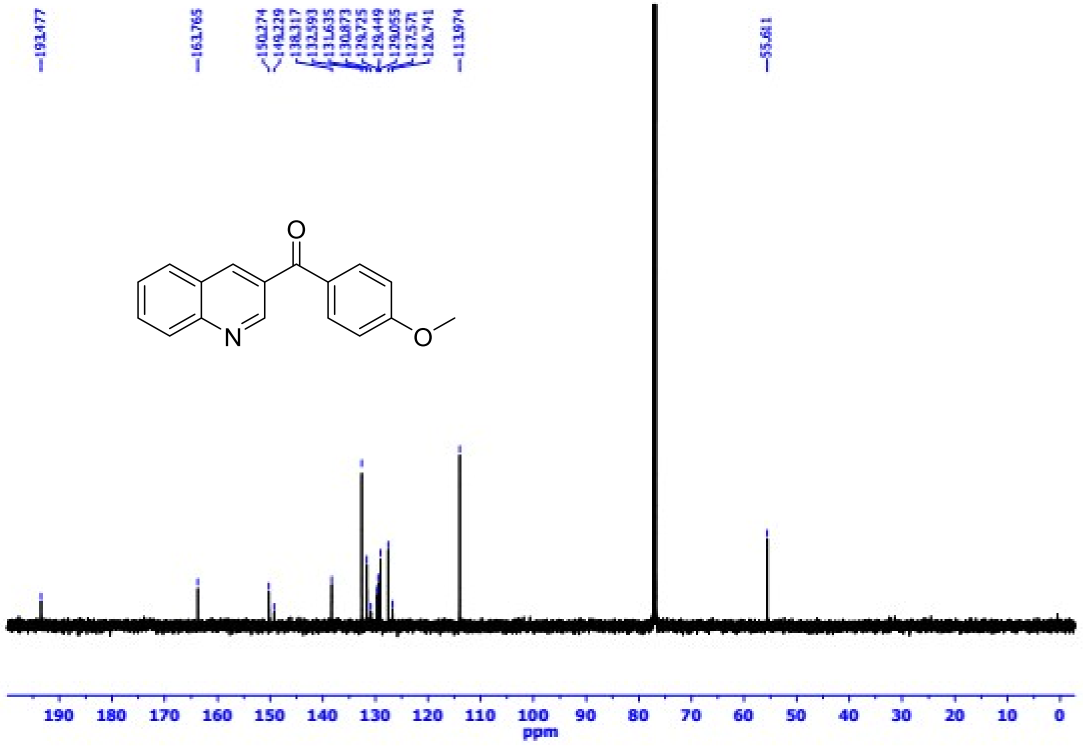


Fig. S21. $^{13}$C-NMR spectra of (4-methoxyphenyl)(quinolin-3-yl)methanone.

**Characterization data for (4-methoxyphenyl)(quinolin-3-yl)methanone**

Prepared as shown in the general experimental procedure and purified on silica gel (hexane/ ethyl acetate =3 /2): white solid, 90% yield. $^{1}$H NMR (500 MHz, $CDCl_3$) δ 9.11 (s, 1H), 8.36 (d, *J* = 1.7 Hz, 1H), 8.03 (d, *J* = 8.5 Hz, 1H), 7.81 – 7.64 (m, 4H), 7.47 (t, *J* = 7.5 Hz, 1H), 6.88 – 6.82 (m, 2H), 3.75 (s, 3H). $^{13}$C NMR (126 MHz, $CDCl_3$) δ 193.5, 163.8, 150.3, 149.2, 138.3, 132.6, 131.6, 130.9, 129.7, 129.4, 129.1, 127.6, 126.7, 114.0, 55.6.

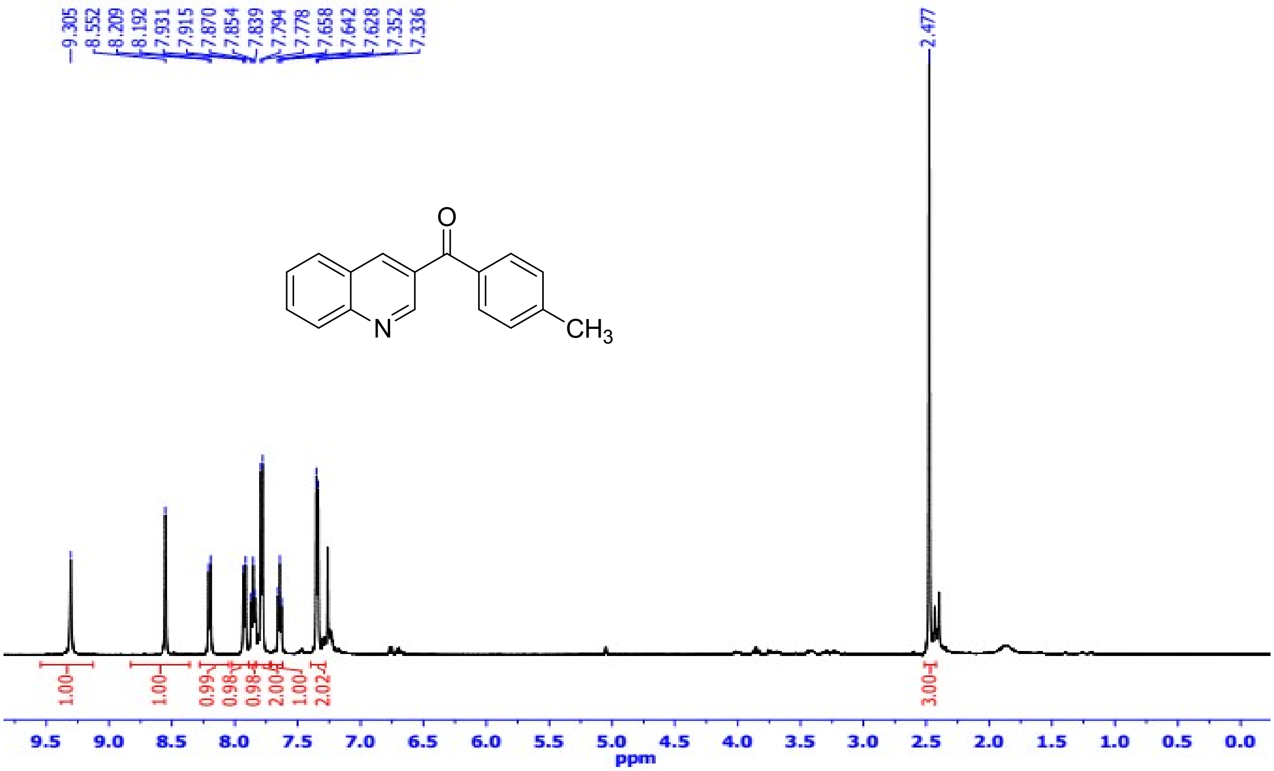


Fig. S22. $^{1}$H-NMR spectra of quinolin-3-yl(p-tolyl)methanone.

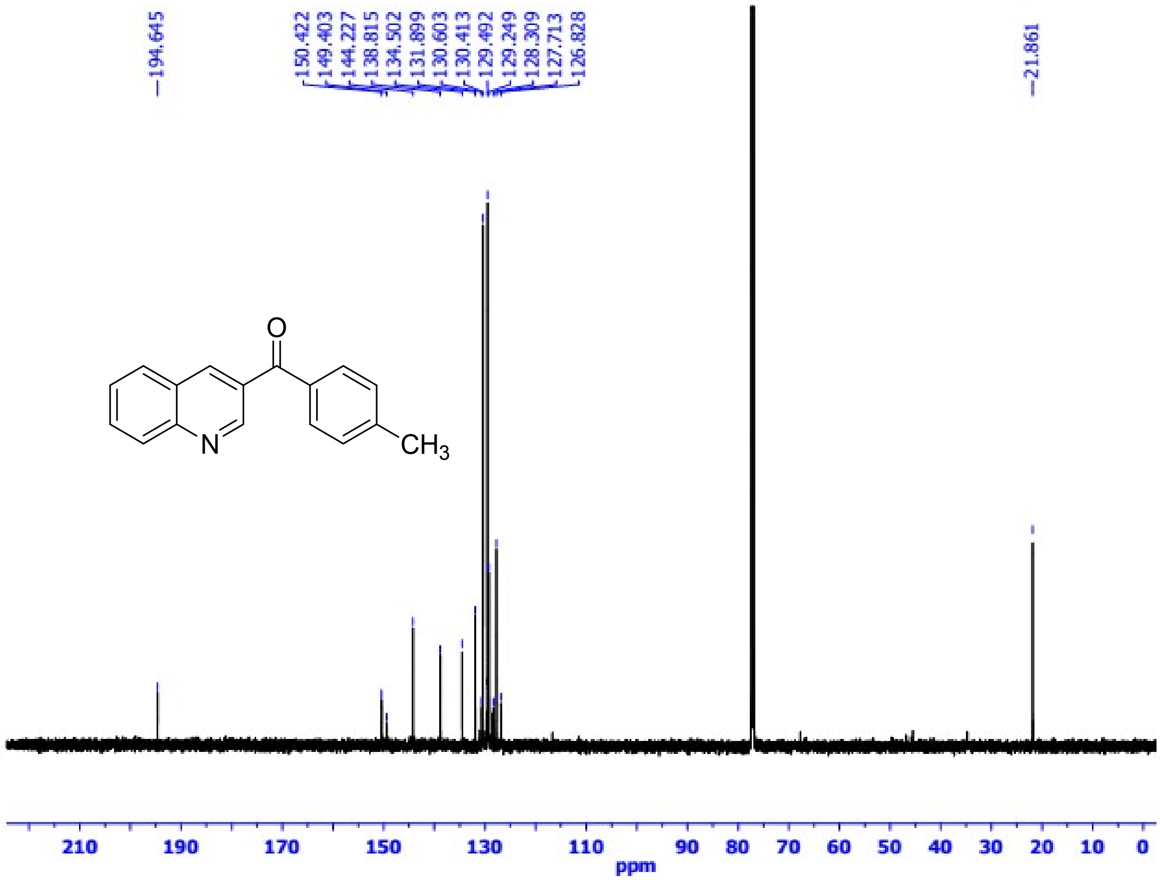


Fig. S23. $^{13}$C-NMR spectra of quinolin-3-yl(p-tolyl)methanone.

**Characterization data for quinolin-3-yl(p-tolyl)methanone**

Prepared as shown in the general experimental procedure and purified on silica gel (hexane/ ethyl acetate =3 /2): white solid, 85% yield. $^1$H NMR (500 MHz, $CDCl_3$) δ 9.31 (s, 1H), 8.55 (s, 1H), 8.20 (d, *J* = 8.4 Hz, 1H), 7.92 (d, *J* = 8.1 Hz, 1H), 7.85 (t, *J* = 7.7 Hz, 1H), 7.79 (d, *J* = 8.0 Hz, 2H), 7.64 (t, *J* = 7.5 Hz, 1H), 7.34 (d, *J* = 7.9 Hz, 2H), 2.48 (s, 3H). $^{13}$C NMR (126 MHz, CDCl3) δ 194.6, 150.4, 149.4, 144.2, 138.8, 134.5, 131.9, 130.6, 130.4, 129.5, 129.2, 128.3, 127.7, 126.8, 21.9.

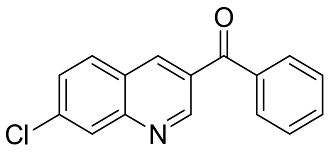


Fig. S24. $^{1}$H-NMR spectra of (7-chloroquinolin-3-yl)(phenyl)methanone.

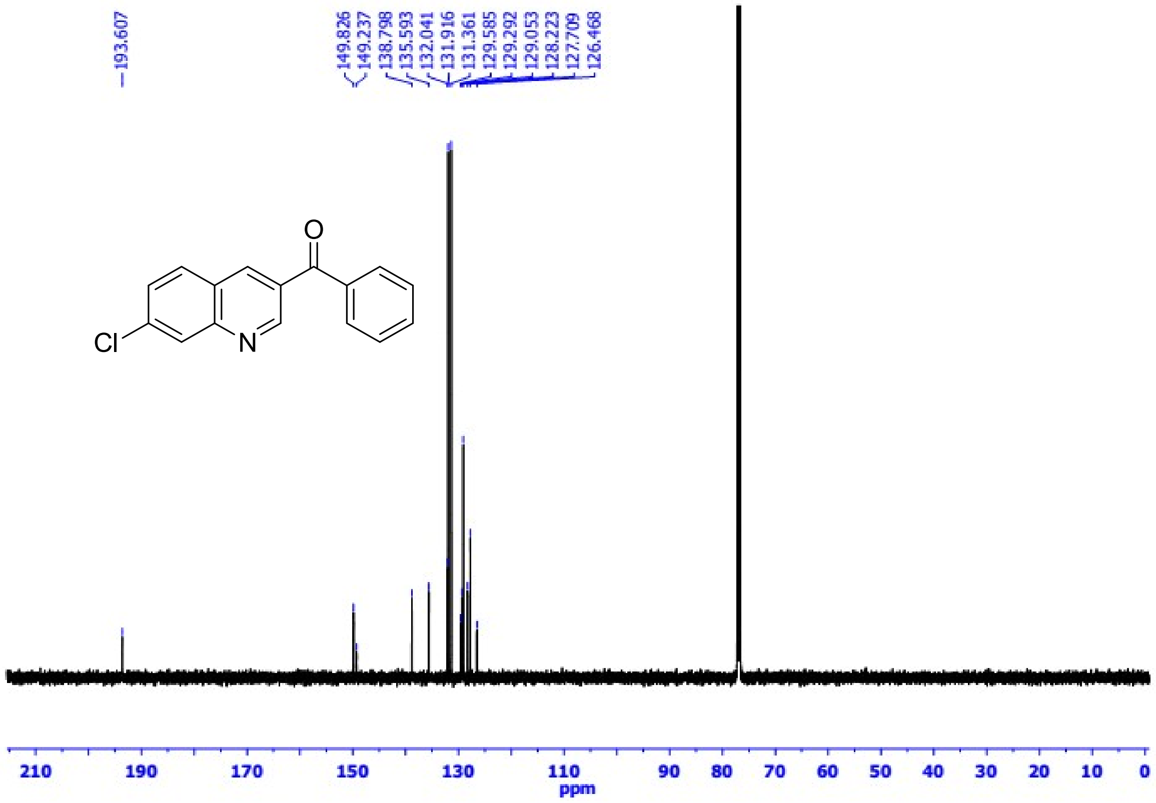


Fig. S25. $^{13}$C-NMR spectra of (7-chloroquinolin-3-yl)(phenyl)methanone.

**Characterization data for (7-chloroquinolin-3-yl)(phenyl)methanone**

Prepared as shown in the general experimental procedure and purified on silica gel (hexane/ ethyl acetate =3 /2): white solid, 81% yield. $^{1}$H NMR (500 MHz, $CDCl_3$) δ 9.30 (s, 1H), 8.54 (s, 1H), 8.21 (d, $J$ = 8.4 Hz, 1H), 7.93 (d, $J$ = 8.1 Hz, 1H), 7.88 (t, $J$ = 7.7 Hz, 1H), 7.75 (d, $J$ = 8.4 Hz, 2H), 7.72 – 7.65 (m, 3H). $^{13}$C NMR (126 MHz, CDCl3) δ 193.6, 149.8, 149.2, 138.8, 135.6, 132.0, 131.9, 131.4, 129.6, 129.3, 129.1, 128.2, 127.7, 126.5.

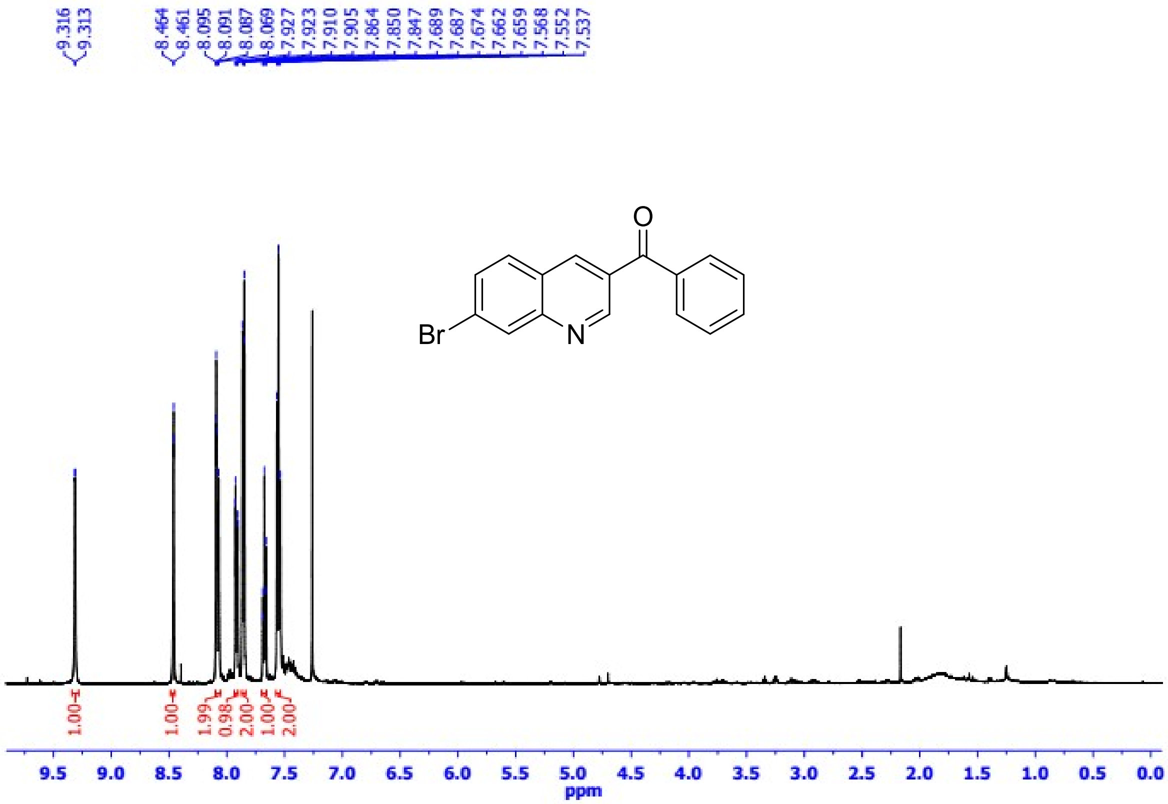


Fig. S26. $^{1}$H-NMR spectra of (7-bromoquinolin-3-yl)(phenyl)methanone.

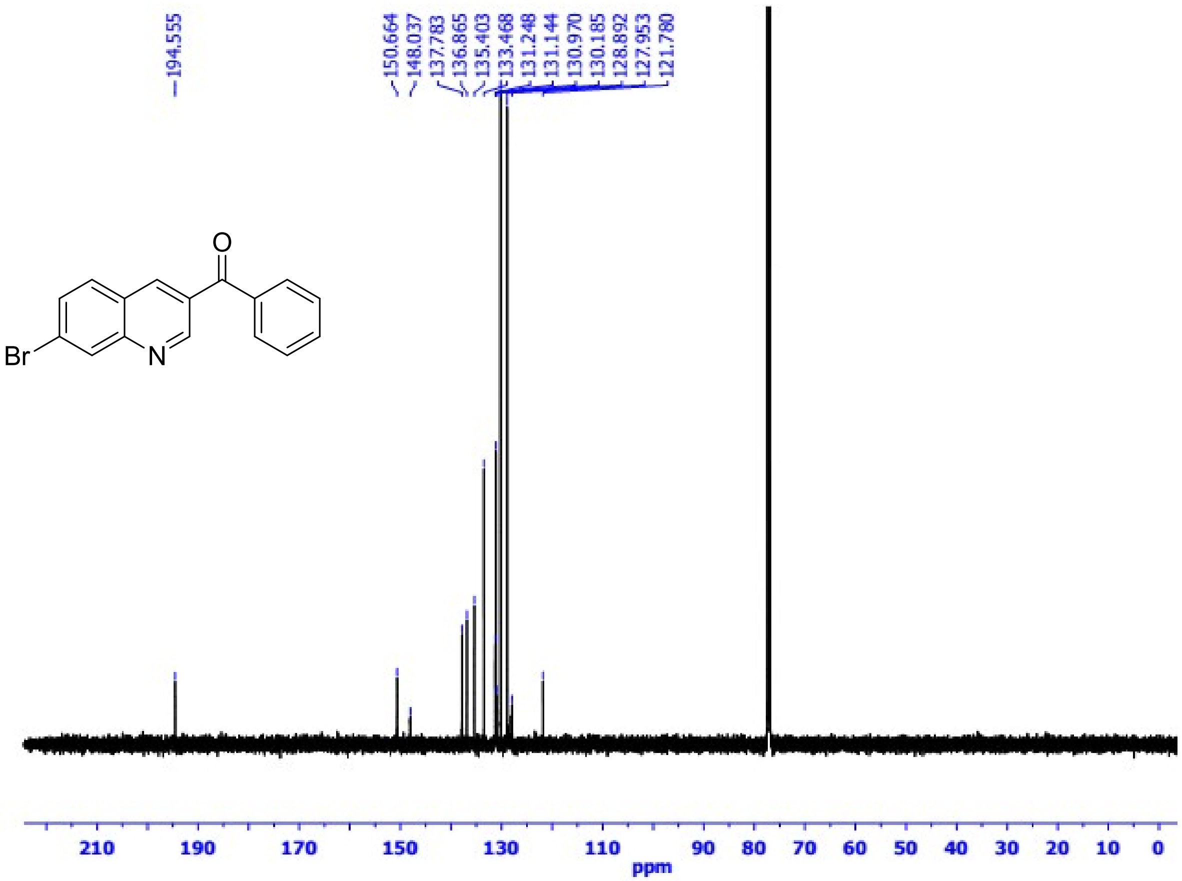


Fig. S27. $^{13}$C-NMR spectra of (7-bromoquinolin-3-yl)(phenyl)methanone.

**Characterization data for (7-bromoquinolin-3-yl)(phenyl)methanone.**

Prepared as shown in the general experimental procedure and purified on silica gel (hexane/ ethyl acetate =7/ 3): yellow solid, 87% yield. $^{1}$H NMR (500 MHz, $CDCl_3$) δ 9.31 (d, $J$ = 1.3 Hz, 1H), 8.46 (d, $J$ = 1.8 Hz, 1H), 8.09 (dd, $J$ = 7.5, 5.7 Hz, 2H), 7.92 (dd, $J$ = 9.0, 2.1 Hz, 1H), 7.87 – 7.84 (m, 2H), 7.70 – 7.66 (m, 1H), 7.55 (t, $J$ = 7.7 Hz, 2H). $^{13}$C NMR (126 MHz, $CDCl_3$) δ 194.6, 150.7, 148.0, 137.8, 136.9, 135.4, 133.5, 131.3, 131.1, 131.0, 130.2, 128.9, 127.9, 121.8.

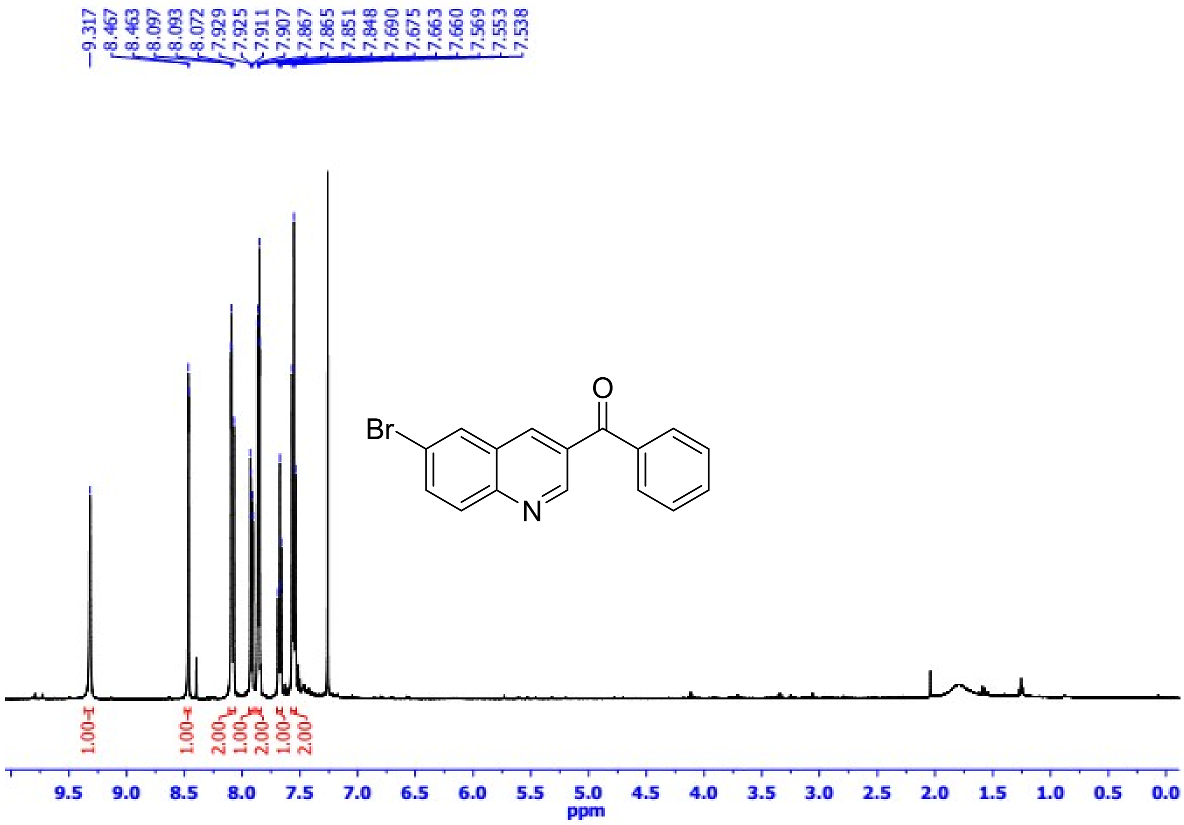


Fig. S28. $^{1}$H-NMR spectra of (6-bromoquinolin-3-yl)(phenyl)methanone.

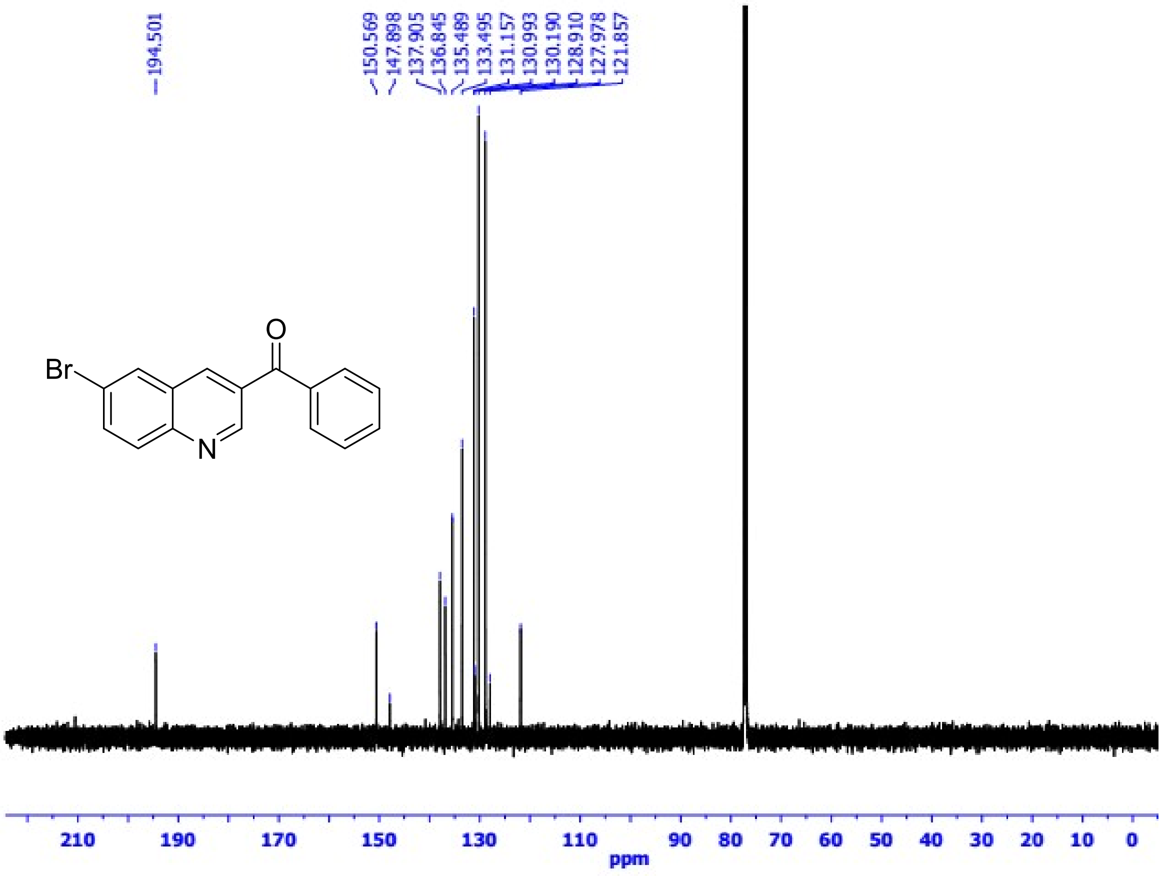


Fig. S29. $^{13}C$-NMR spectra of (6-bromoquinolin-3-yl)(phenyl)methanone.

**Characterization data for (6-bromoquinolin-3-yl)(phenyl)methanone.**

Prepared as shown in the general experimental procedure and purified on silica gel (hexane/ ethyl acetate =7/ 3): yellow solid, 85% yield. $^{1}H$ NMR (500 MHz, $CDCl_3$) δ 9.32 (s, 1H), 8.47 (d, $J$ = 1.9 Hz, 1H), 8.11 – 8.06 (m, 2H), 7.92 (dd, $J$ = 9.0, 2.2 Hz, 1H), 7.86 (dd, $J$ = 8.2, 1.2 Hz, 2H), 7.67 (dd, $J$ = 10.6, 4.3 Hz, 1H), 7.55 (t, $J$ = 7.8 Hz, 2H). $^{13}C$ NMR (126 MHz, $CDCl_3$) δ 194.5, 150.6, 147.9, 137.9, 136.8, 135.5, 133.5, 131.2, 131.0, 130.2, 128.9, 128.0, 121.9.

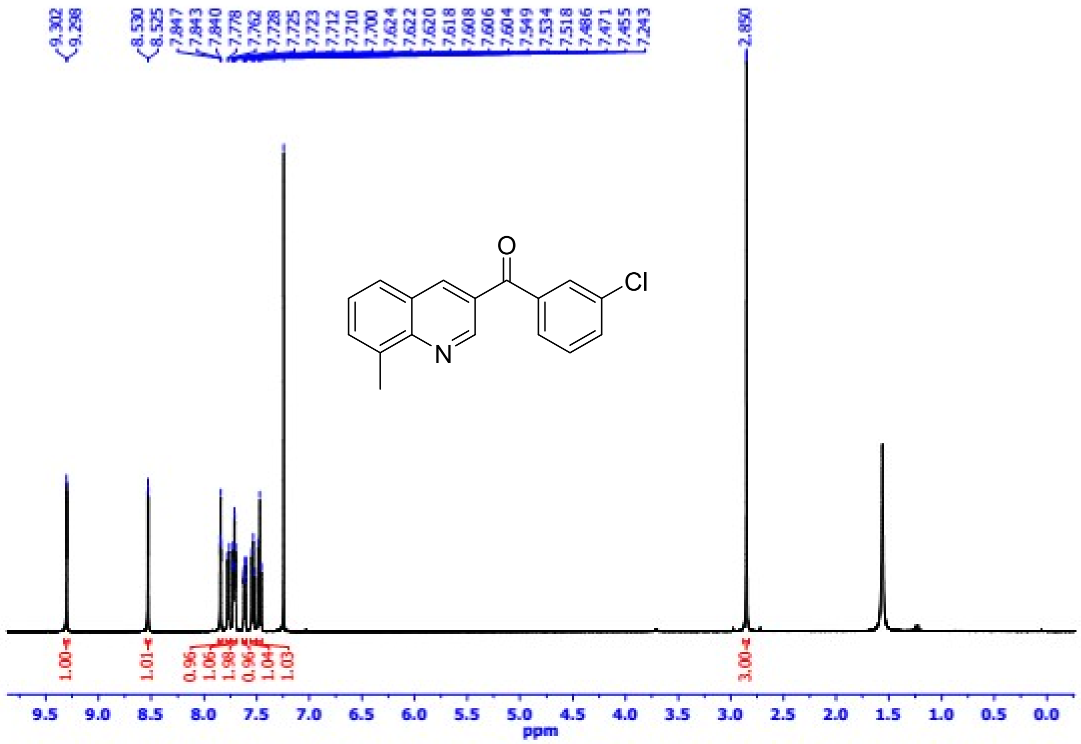


Fig. 30. $^{1}$H-NMR spectra of (3-chlorophenyl)(8-methylquinolin-3-yl)methanone.

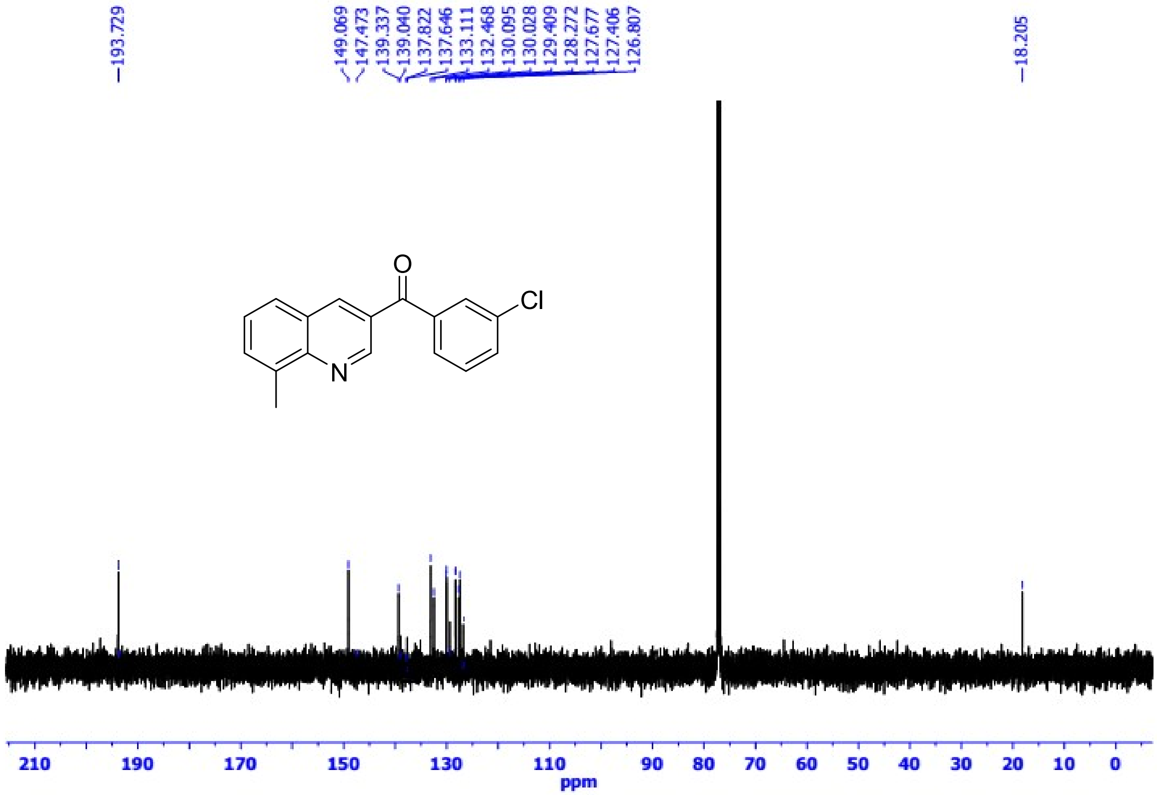


Fig. S31. $^{13}$C-NMR spectra of (3-chlorophenyl)(8-methylquinolin-3-yl)methanone.

**Characterization data for (3-chlorophenyl)(8-methylquinolin-3-yl)methanone**

Prepared as shown in the general experimental procedure and purified on silica gel (hexane/ ethyl acetate = 10/1): light yellow solid, 65% yield. $^{1}$H NMR (500 MHz, $CDCl_3$) δ 9.30 (d, $J$ = 2.2 Hz, 1H), 8.53 (d, $J$ = 2.2 Hz, 1H), 7.84 (t, $J$ = 1.8 Hz, 1H), 7.77 (d, $J$ = 8.1 Hz, 1H), 7.74 – 7.69 (m, 2H), 7.64 – 7.60 (m, 1H), 7.56 – 7.51 (m, 1H), 7.47 (t, $J$ = 7.8 Hz, 1H), 2.85 (s, 3H). $^{13}$C NMR (126 MHz, $CDCl_3$) δ 193.7, 149.1, 147.5, 139.3, 139.0, 137.8, 137.6, 133.1, 132.5, 130.1, 130.0, 129.4, 128.3, 127.7, 127.4, 126.8, 18.3.

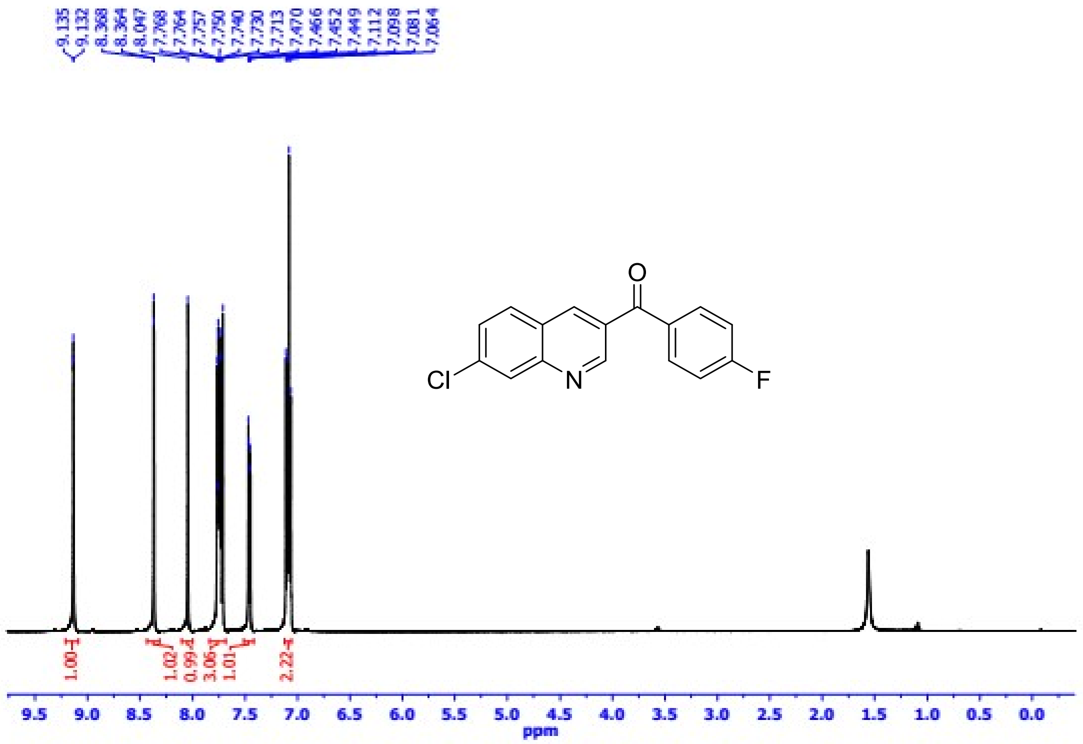


Fig. S32. $^{1}$H-NMR spectra of (7-chloroquinolin-3-yl)(4-fluorophenyl)methanone.

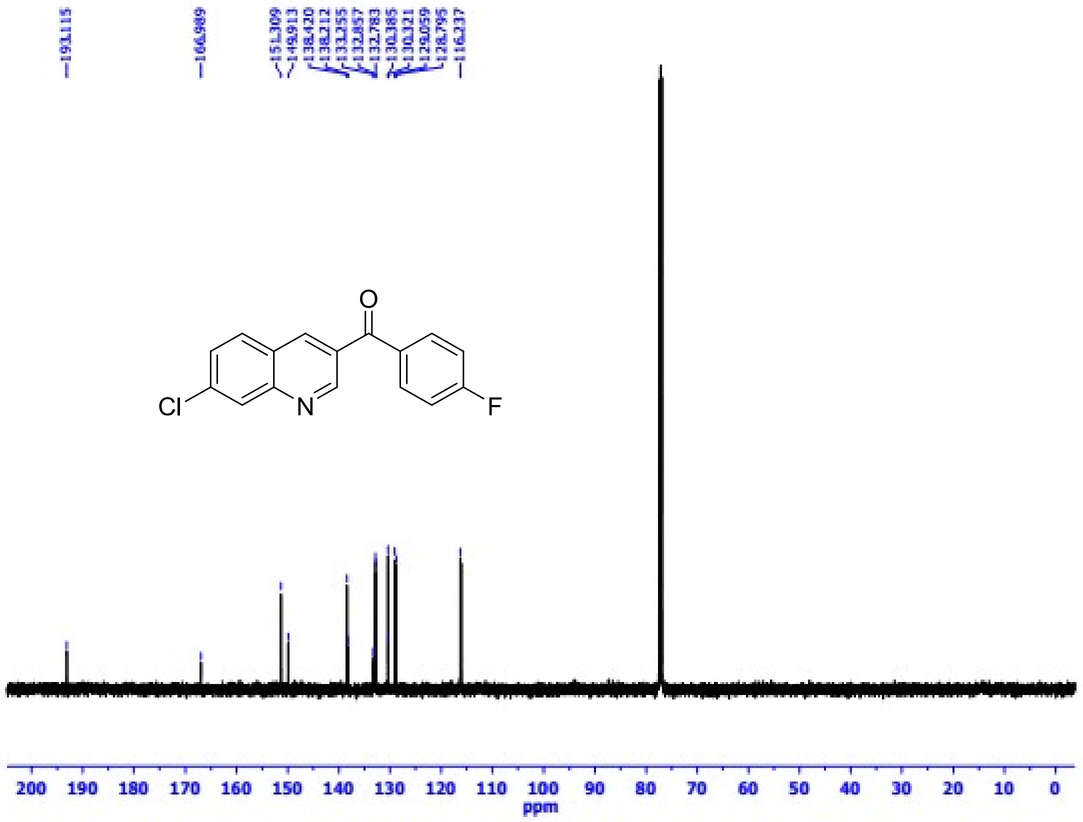


Fig. S33. $^{13}$C-NMR spectra of (7-chloroquinolin-3-yl)(4-fluorophenyl)methanone.

**Characterization data for (7-chloroquinolin-3-yl)(4-fluorophenyl)methanone**

Prepared as shown in the general experimental procedure and purified on silica gel (hexane/ ethyl acetate = 4/1): yellow solid, 92% yield. $^1$H NMR (500 MHz, $CDCl_3$) δ 9.13 (d, $J$ = 1.7 Hz, 1H), 8.37 (d, $J$ = 1.7 Hz, 1H), 8.05 (s, 1H), 7.84 – 7.69 (m, 3H), 7.46 (dd, $J$ = 8.7, 1.9 Hz, 1H), 7.06 – 7.11 (m, 2H). $^{13}$C NMR (126 MHz, $CDCl_3$) δ 193.1, 167.0, 151.3, 149.9, 138.4, 138.2, 133.3, 132.9, 132.8, 130.4, 130.3, 129.1, 128.8, 116.2.

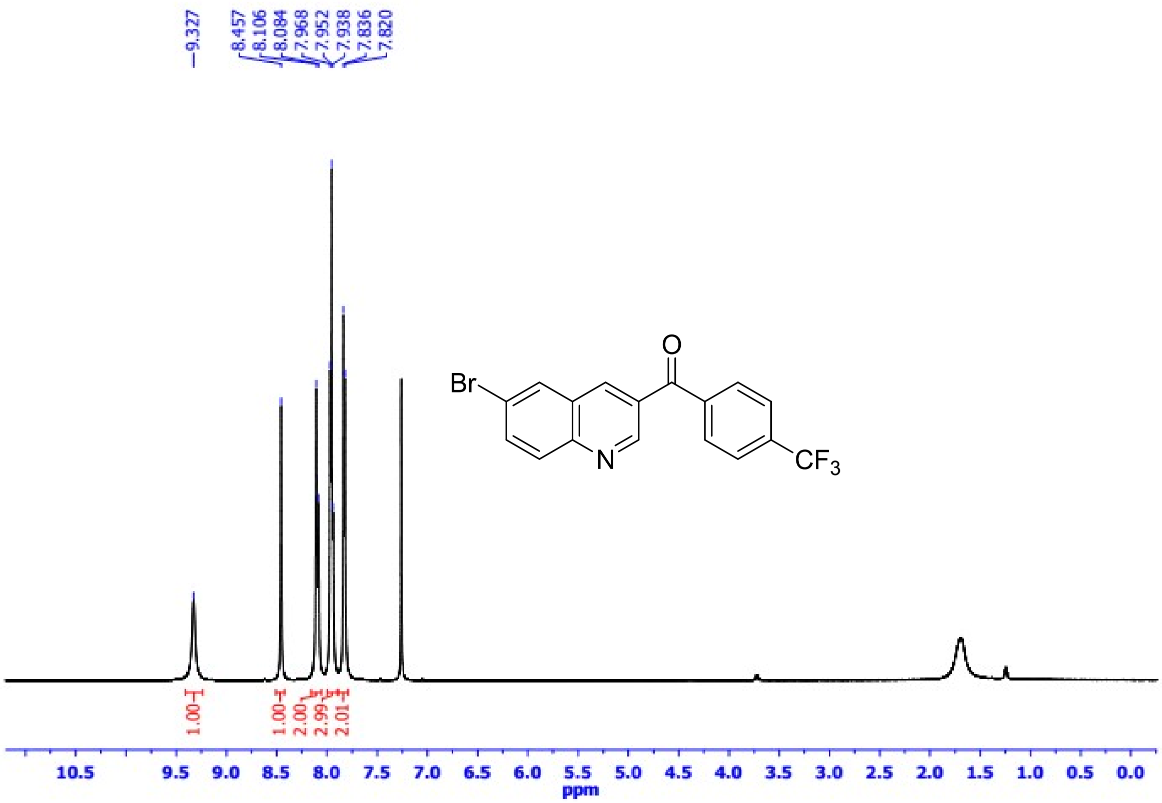


Fig. S34. $^1$H-NMR spectra of (6-bromoquinolin-3-yl)(4-(trifluoromethyl)phenyl)methanone.

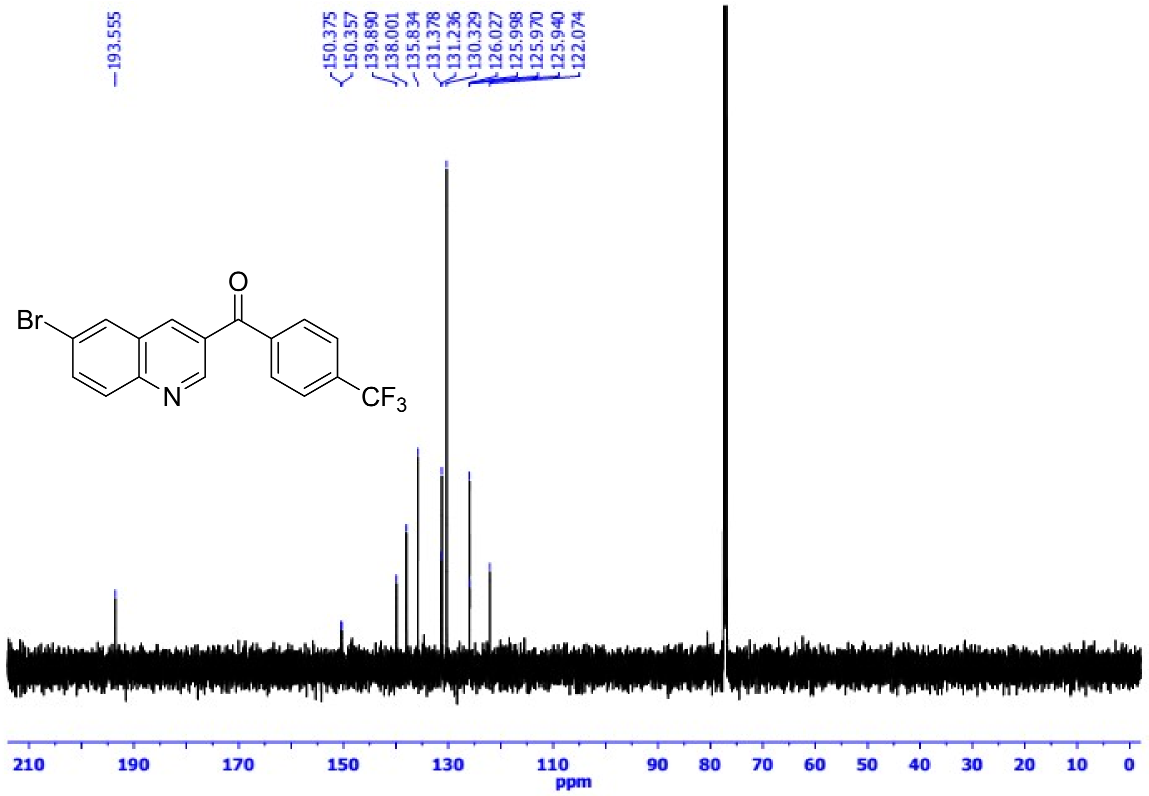


Fig. S35. $^{13}$C-NMR spectra of (6-bromoquinolin-3-yl)(4-(trifluoromethyl)phenyl)methanone.

**Characterization data for (6-bromoquinolin-3-yl)(4-(trifluoromethyl)phenyl)methanone**

Prepared as shown in the general experimental procedure and purified on silica gel (hexane/ ethyl acetate = 4/1): yellow solid, 83% yield. $^{1}$H NMR (500 MHz, CDCl3) δ 9.33 (s, 1H), 8.46 (s, 1H), 8.08 – 8.10(m, 2H), 7.95 (t, *J* = 7.4 Hz, 3H), 7.83 (d, *J* = 8.0 Hz, 2H). $^{13}$C NMR (126 MHz, CDCl3) δ 193.6, 150.4, 150.4, 139.9, 138.0, 135.8, 131.4, 131.2, 130.3, 126.0, 126.0, 126.0, 125.9, 122.1.

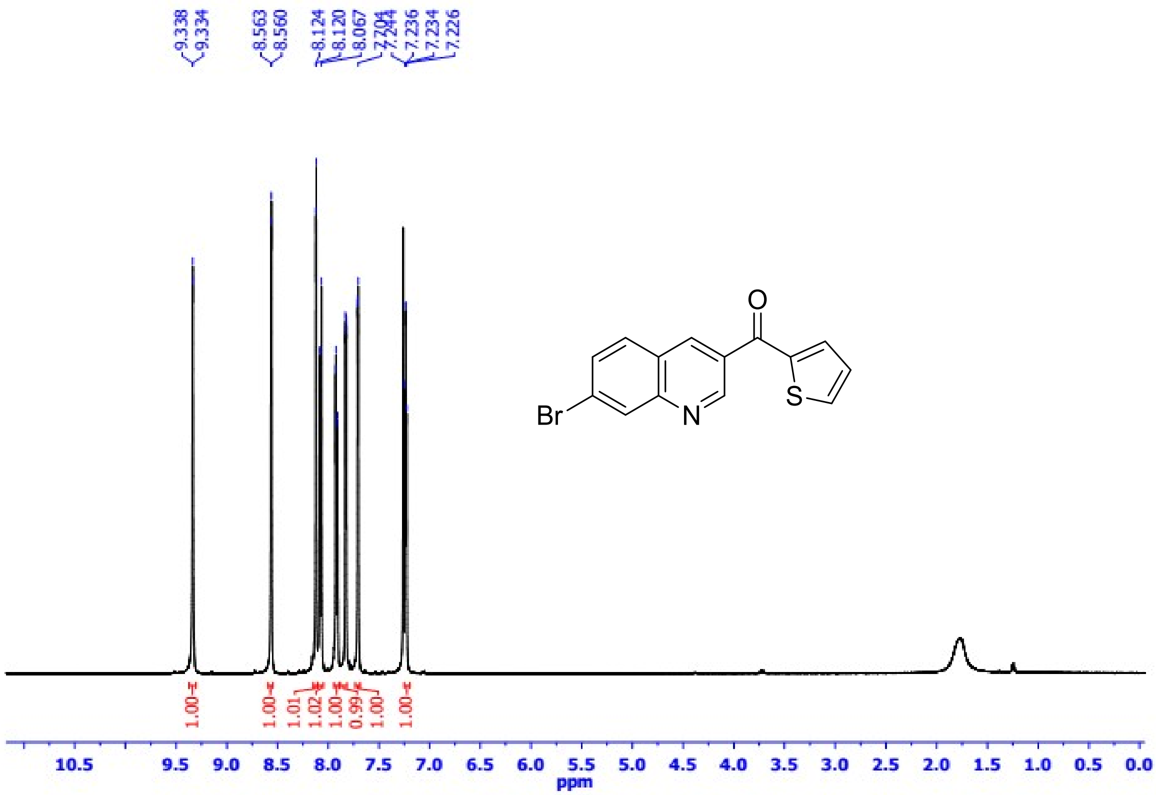


Fig. S36. $^{1}$H-NMR spectra of (7-bromoquinolin-3-yl)(thiophen-2-yl)methanone.

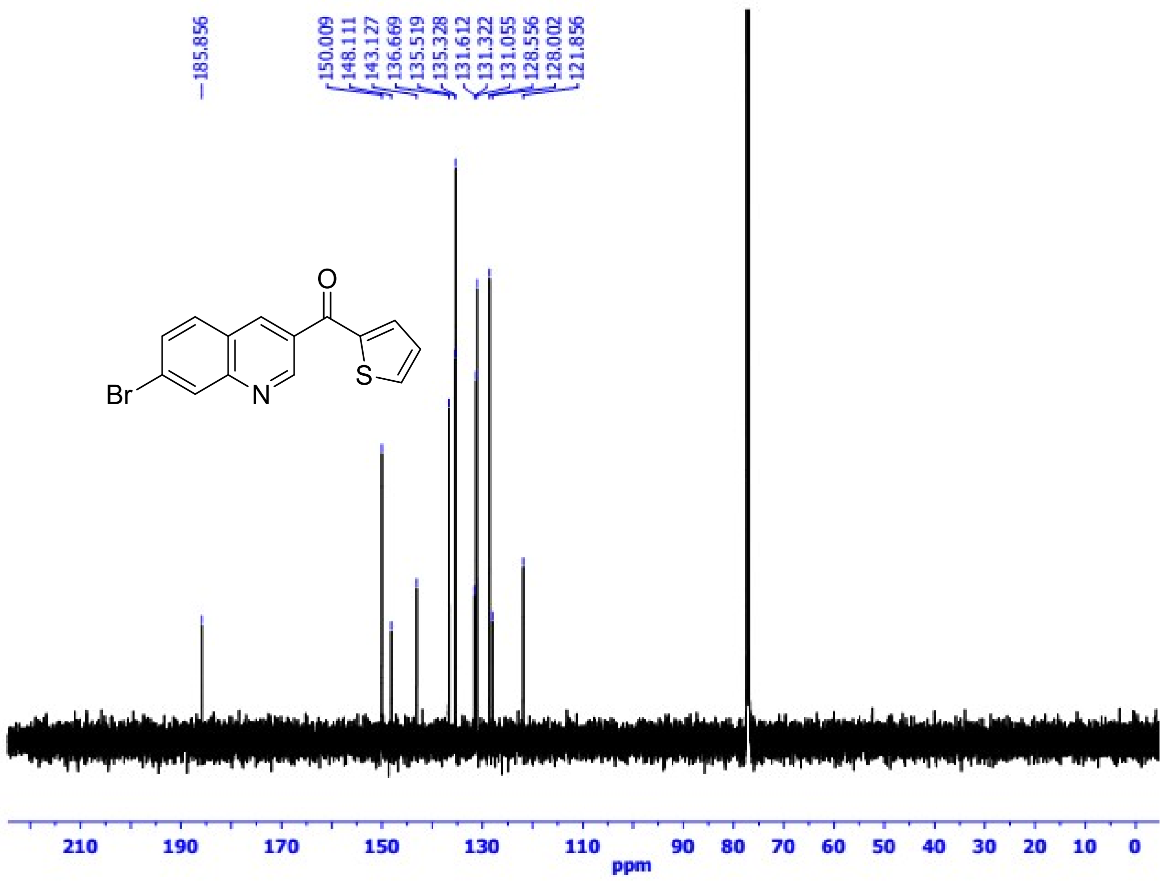


Fig. S37. $^{13}$C-NMR spectra of (7-bromoquinolin-3-yl)(thiophen-2-yl)methanone.

**Characterization data for (7-bromoquinolin-3-yl)(thiophen-2-yl)methanone.**

Prepared as shown in the general experimental procedure and purified on silica gel (hexane/ ethyl acetate = 4/1): yellow solid, 87% yield. $^1$H NMR (500 MHz, CDCl3) δ 9.34 (d, $J$ = 1.8 Hz, 1H), 8.56 (d, $J$ = 1.6 Hz, 1H), 8.12 (d, $J$ = 2.0 Hz, 1H), 8.08 (d, $J$ = 9.0 Hz, 1H), 7.92 (dd, $J$ = 9.0, 2.1 Hz, 1H), 7.83 (dd, $J$ = 4.9, 0.9 Hz, 1H), 7.73 – 7.68 (m, 1H), 7.23 (dd, $J$ = 4.8, 3.9 Hz, 1H). $^{13}$C NMR (126 MHz, CDCl3) δ 185.9, 150.0, 148.1, 143.1, 136.7, 135.5, 135.3, 131.6, 131.3, 131.1, 128.6, 128.0, 121.9.

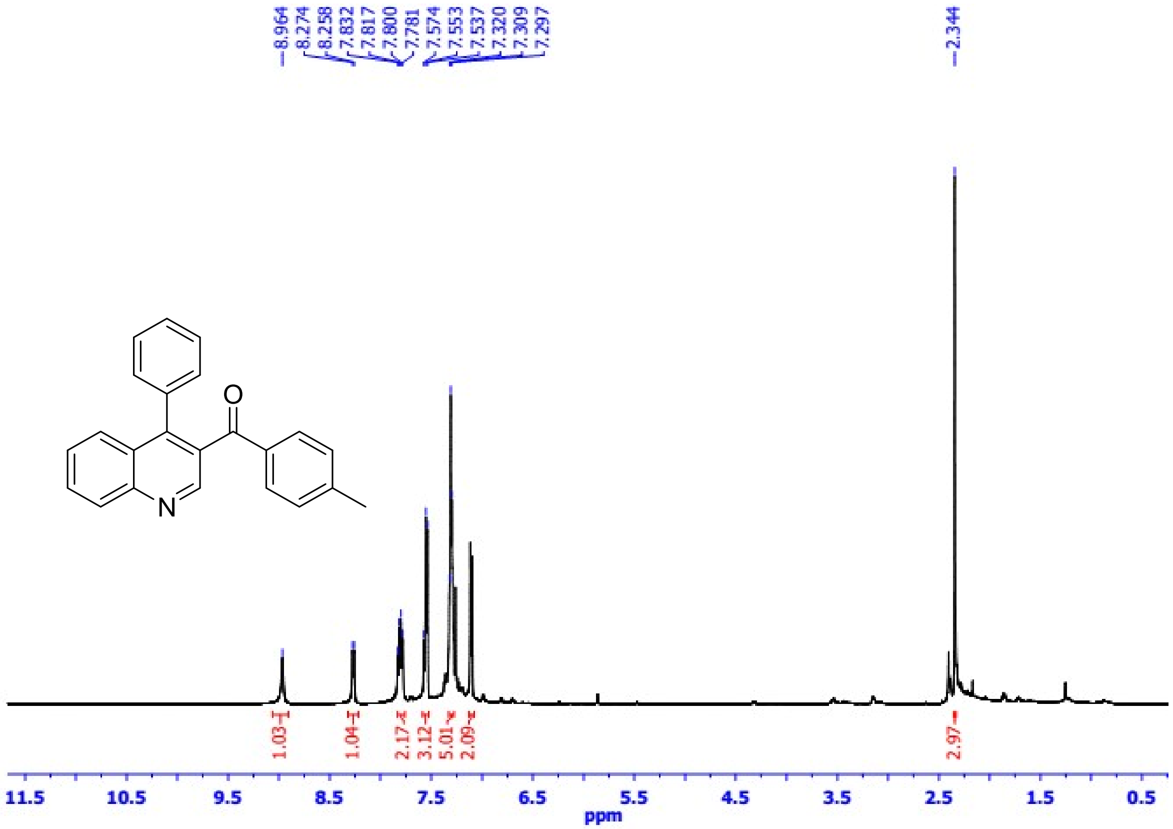


Fig. S38. $^{1}$H-NMR spectra of (4-phenylquinolin-3-yl)(p-tolyl)methanone.

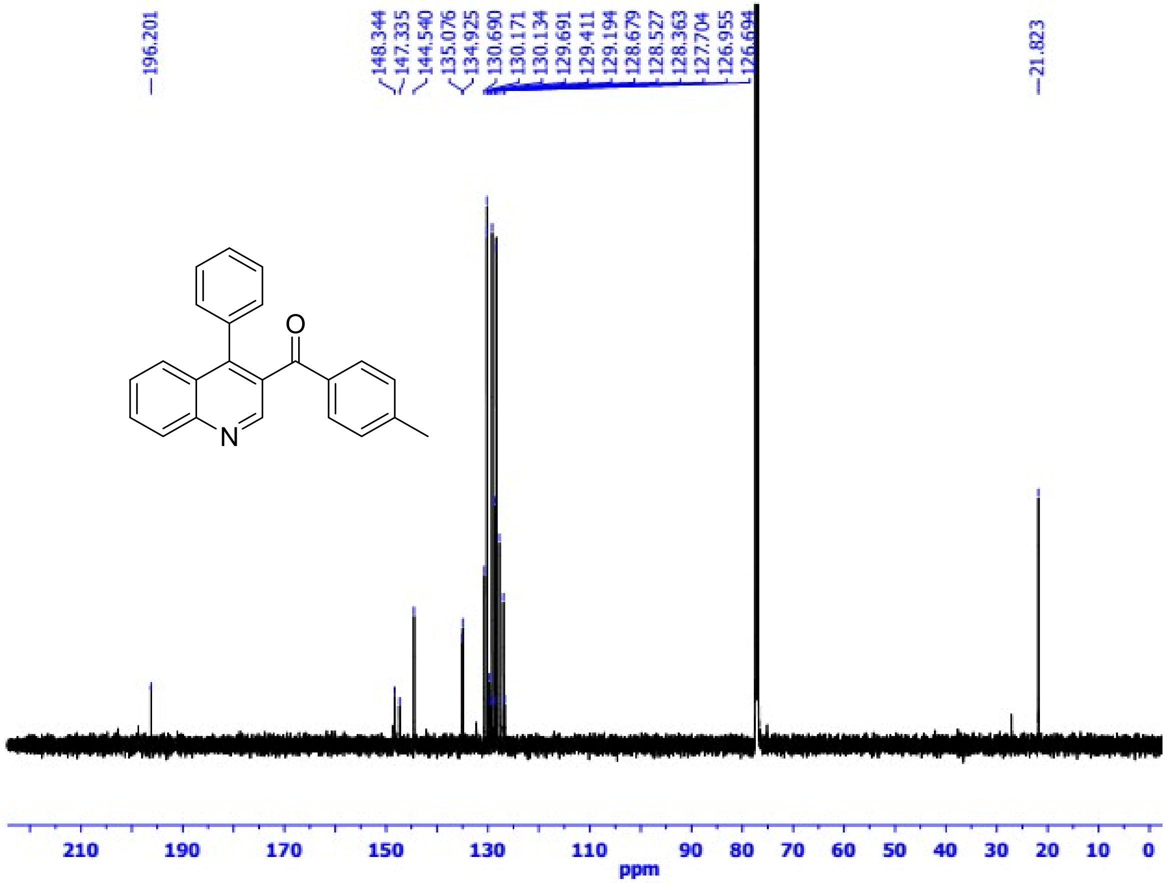


Fig. S39. $^{13}$C-NMR spectra of (4-phenylquinolin-3-yl)(p-tolyl)methanone.

**Characterization data for (4-phenylquinolin-3-yl)(p-tolyl)methanone.**

Prepared as shown in the general experimental procedure and purified on silica gel (hexane/ ethyl acetate = 4/1): yellow liquid, 63% yield. $^{1}$H NMR (500 MHz, CDCl3) δ 8.96 (s, 1H), 8.27 (d, *J* = 8.3 Hz, 1H), 7.18 – 7.83 (m, 2H), 7.54 – 7.57 (m, 3H), 7.30 – 7.32 (m, 5H), 7.11 (d, *J* = 7.9 Hz, 2H), 2.34 (s, 3H). $^{13}$C NMR (126 MHz, CDCl3) δ 196.2, 148.3, 147.3, 144.5, 135.1, 134.9, 130.7, 130.2, 130.1, 129.7, 129.4, 129.2, 128.7, 128.5, 128.4, 127.7, 126.9, 126.7, 21.8.

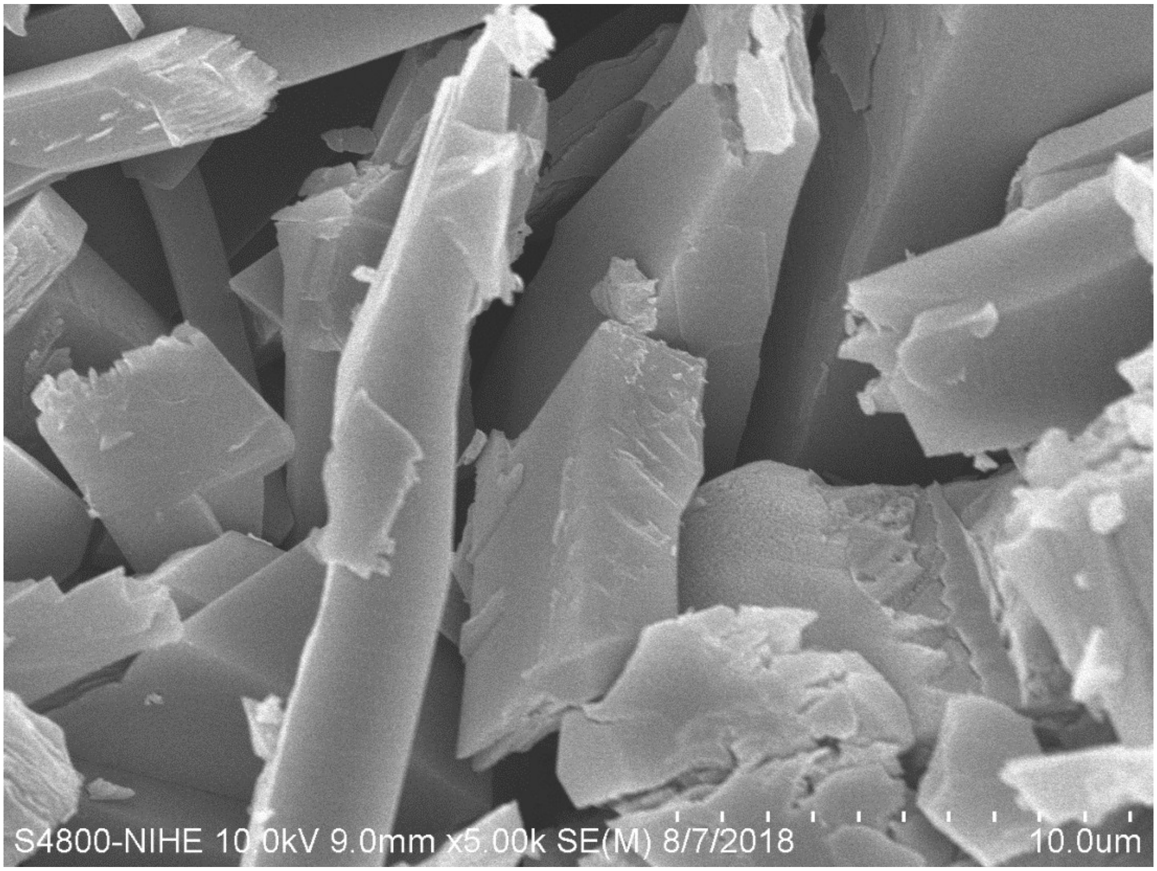


Fig. S40. SEM micrograph of recovered $Cu_2(OBA)_2(BPY)$.

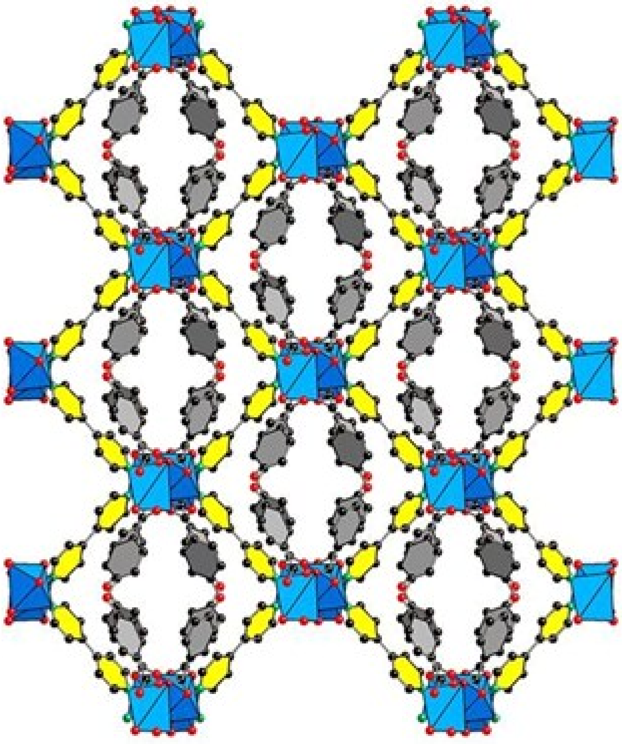

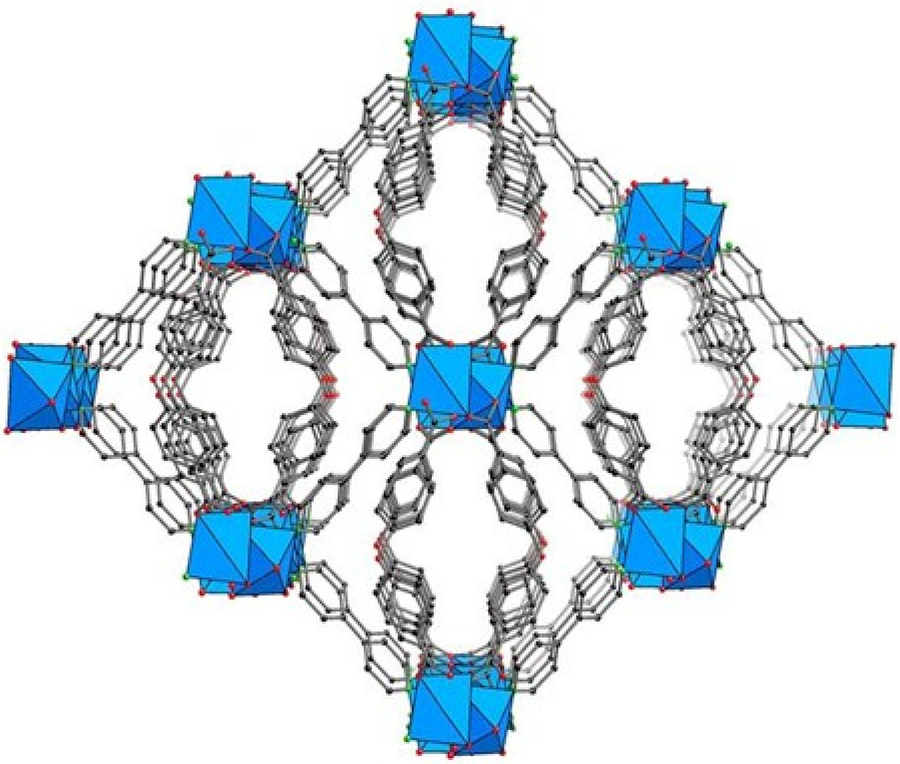

Fig. S41. Structure of the $Cu_2(OBA)_2(BPY)$. Copper sites in the Cu-MOF are connected by the $COO^-$ groups in the 4,4′-oxybis(benzoate) ligands to generate an eight-membered ring chains. The connectivity between the corner-shared eight-membered ring chains is additionally strengthen by the bent 4,4′-oxybis(benzoate) ligands to form 2D helical layer including the right-handed helical chains. Moreover, 4,4′-bipyridine functions as pillars between adjacent helical layers, producing the framework. (Reference: L. Tang, D. Li, F. Fu, Y. Wu, Y. Wang, H. Hu and E. Wang, *J. Mol. Struct.*, 2008, **888**, 344-353)

*Literature yields of the 3-acylquinolines synthesis:*

1.

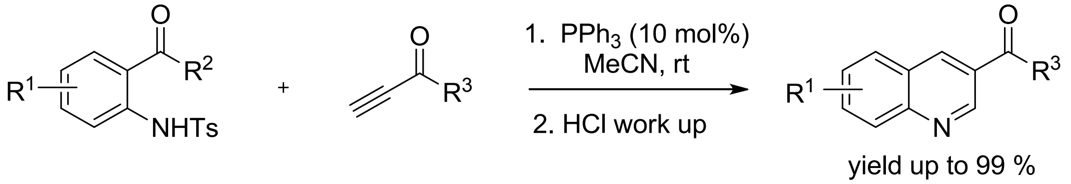


(Reference: Khong and Kwon, "One-Pot Phosphine-Catalyzed Syntheses of Quinolines", *The Journal of Organic Chemistry*, 2012, **77**, 8257-8267)

2.

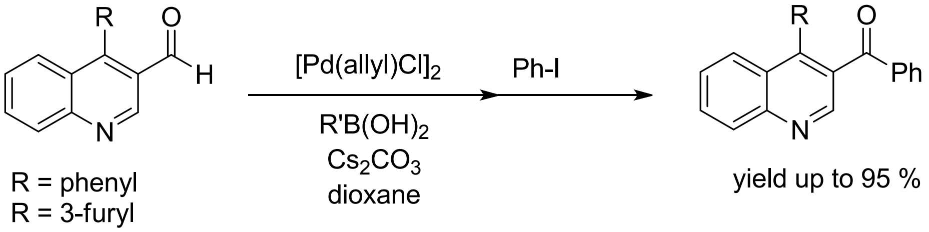


(Reference: Kuriyama et al. "One-Pot Synthesis of Heteroaryl and Diheteroaryl Ketones through Palladium-Catalyzed 1,2-Addition and Oxidation", *European Journal of Organic Chemistry,* 2013, **16**, 3378-3385)

3.

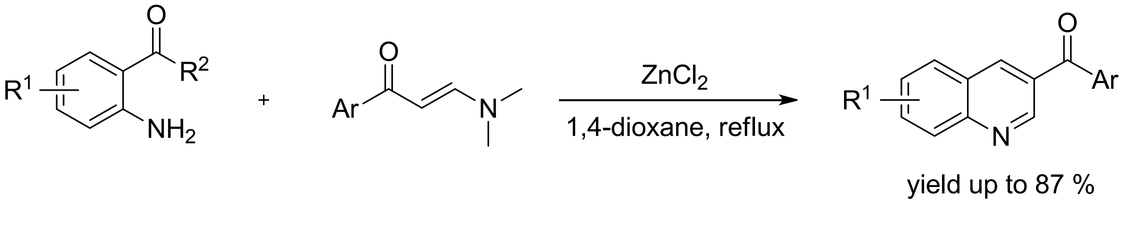


(Reference: Luo et al. "$ZnCl_2$-Promoted Friedländer-type Synthesis of 4-Substituted 3-Aroyl Quinolines from o-Aminoaryl Ketones and Enaminones", *Tetrahedron Letters*, 2016, **57**, 4987-4990)

4.

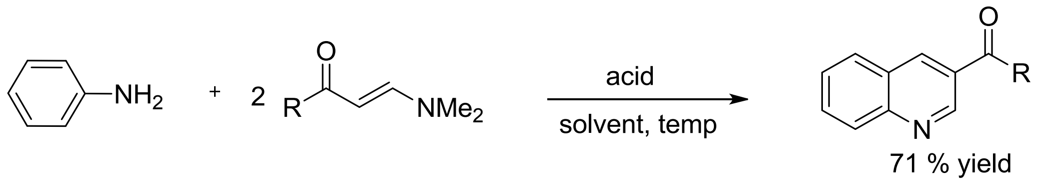


(Reference: Wan et al. "Branched C=C and C-N Bond Cleavage on Enaminones toward the Synthesis of 3-Acyl Quinolines" *Asian Journal of Organic Chemistry*, 2017, **6**, 666-668)

5.

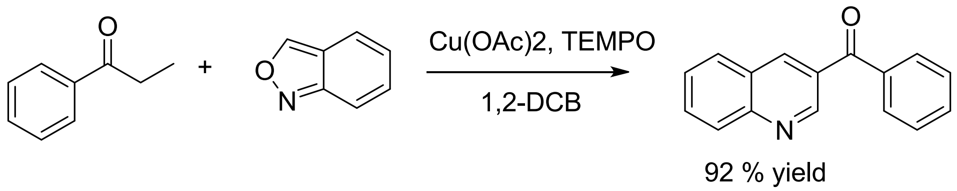


(Reference: Tiwari et al. "α, β-Functionalization of Saturated Ketones with Anthranils via Cu-Catalyzed Sequential Dehydrogenation/aza-Michael Addition/Annulation Cascade Reactions in One-Pot", *Chemical Communications*, 2017, **53**, 5302-5305)

6.

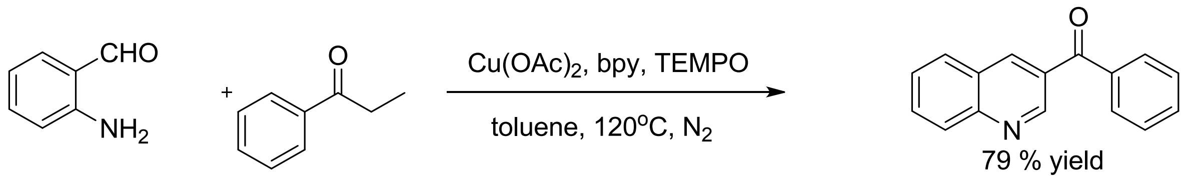


(Reference: Wang et al. "Synthesis of 3-Acylquinolines through Cu-Catalyzed Double C ($sp^3$)-H Bond Functionalization of Saturated Ketones", *Organic Chemistry Frontiers*, 2017, **4**, 612-616)
